# Quaternary Phosphonium Salts Outperformed Vemurafenib (PLX) and Etoposide Against BRAF^V600D,V600E^ PLX-Resistant Melanoma and MDR Neuroblastoma, Exhibiting No/Low Toxicity on 3T3/HaCaT Cells

**DOI:** 10.3390/ijms27073170

**Published:** 2026-03-31

**Authors:** Silvana Alfei, Maria Grazia Signorello, Sara Tirendi, Elaheh Khaledizadeh, Paolo Giordani, Caterina Reggio, Barbara Marengo, Cinzia Domenicotti

**Affiliations:** 1 Department of Pharmacy, University of Genoa, Viale Cembrano, 16148 Genoa, Italy; mariagrazia.signorello@unige.it (M.G.S.); paolo.giordani@unige.it (P.G.); 2 Biochemistry Laboratory, Department of Pharmacy, University of Genoa, Viale Benedetto XV 3, 16132 Genoa, Italy; 3 Department of Experimental Medicine (DIMES), University of Genova, Via Alberti L.B., 16132 Genoa, Italy; sara.tirendi@edu.unige.it (S.T.); elaheh.khaledizadeh@edu.unige.it (E.K.); cinzia.domenicotti@unige.it (C.D.); 4 Inter-University Center for the Promotion of the 3Rs Principles in Teaching & Research (Centro 3R), 56122 Pisa, Italy; 5 Laboratory of Experimental Therapies in Oncology, IRCCS Istituto Giannina Gaslini, Via G. Gaslini 5, 16147 Genoa, Italy; caterinareggio@gaslini.org; 6 IRCCS Azienda Ospedaliera Metropolitana, 16132 Genoa, Italy

**Keywords:** metastatic cutaneous melanoma (MCM), high-risk neuroblastoma (HR-NB), quaternized phosphonium salts (QPSs), micro vesicles, cell viability (%), haemolytic effects, cytotoxicity on HaCaT and 3T3 cells

## Abstract

Late-stage metastatic cutaneous melanoma (MCM) and neuroblastoma (NB) are the most aggressive skin and childhood cancers with survival rates of <50%, mainly due to the emergence of resistance to available drugs, thus requiring an urgent solution. Quaternary phosphonium salts (QPSs) can exhibit strong anticancer effects, regardless of the developed resistance. Triphenyl (**1**) and diphenyl (**3** and **4**) phosphonium salts were synthesized, treating commercial triphenyl phosphine and synthesizing 11-diphenylphosphanyl-undecan-1-ol (**2**), respectively, with benzyl bromide. Upon full characterization, they were tested, for the first time, on MeTRAV (BRAF^V600D^) and MeOV (BRAF^V600E^) vemurafenib (PLX)-resistant MCM cells, etoposide (ETO)-sensitive (HTLA 230) and multidrug resistant (MDR) (HTLA ER) NB cells, non-tumorigenic human keratinocytes (HaCaT), and mouse embryonic fibroblasts (3T3), as well as red blood cells (RBCs). Viability of MeTRAV cells was decreased to 44.8% by administration of **1** (100 µM), in intermediate-time (48 h) treatments, while short-time exposure (24 h) to **3** (≥75 µM) and **4** (≥50 µM) was sufficient to reduce their viability to 33.6 and 32.2%. Viability of MeOV was decreased under 50% with 5 µM concentrations of **1** and 25 µM of **3** and **4**, While cells were exterminated (26.9, 20.6, and 21.8%) with higher concentrations after 48 h exposure. Collectively, **1** was the better performing compound (IC_50_ = 6.4 µM, 48 h). Viability of HTLA ER cells was decreased under 50% upon 72 h administrations of **1** at concentrations ≥ 50 µM, 48 h (≥75 µM) and 72 h (≥50 µM) of **3**, and after 72 h (≥75 µM) of **4**, but 72 h exposure and high concentrations of all compounds were necessary for their extermination (31.2, 28.7, and 29.7%). Viability of HTLA 230 cells was not <50% when **1** and **4** were administered for only 24 h, while their viability was <50% after administration of **3** at all times of exposure. At high concentrations, all compounds exterminated cells (33.6, 25.3%, **1**, 48–72 h; 38.6, 30.2, and 24.7%, **3**, 24–72 h; 33.2%, **4**, 72 h). The best-performing compounds were **1** (IC_50_ = 4.0 µM, HTLA 230) and **3** (IC_50_ = 27.8 µM, HTLA ER) at 72 h exposure. The cytotoxic effects of compound **4** on MeTRAV cells, when exposed to 24/48 h treatments, were comparable to those of PLX on the same cells in 72 h treatments. Compound **1**, in shorter 48 h treatments of PLX-R MeOV, was 2.5-fold more cytotoxic than PLX in 72 h ones. All compounds were not cytotoxic to 3T3 cells at all times of exposure; they had low cytotoxicity to HaCaT cells in 24 and 48 h treatments and were slightly cytotoxic to RBCs in 24 h ones. Compound **1** could be a promising platform to develop new intermediate-time therapies for PLX-R MeOV cells, while **4** could be used to develop 24 and 48 h treatments for PLX-R MeTRAV cells. Also, all compounds could be developed as new treatment options for both ETO-sensitive and MDR late-stage HR-NB cells, being even more effective than ETO by 1.2, 2.0, and 1.3 times (HTLA 230) and 3.2, 4.7, and 3.2 times (HTLA ER). All compounds have the potential to be developed as adjuvants in already existing anticancer cocktails to treat MCM and/or NB.

## 1. Introduction

Cutaneous melanoma (CM) is a malignant tumor that accounts for more than 90% of melanoma diagnoses, especially in young, white populations. It originates from melanocytes, which are cells responsible for the synthesis of melanin, and they are found mostly in the skin [1], but are also present in other tissues and organs [2]. Collectively, melanoma accounts for about 25, 30, and 60 new cases per population of 100,000 in Europe, in the USA, and in Australia and New Zealand [3]. In Italy, CM is currently the third most frequent tumor in both sexes under the age of 50, with recorded diagnoses that reached the number of 17,000 in 2024 [3]. Metastatic cutaneous melanoma (MCM) is triggered by a complex interaction of UVR-mediated oncogenic aberrations such as BRAF, NRAS, or KIT mutations with phenotypic risk factors, including lighter skin tones, sun sensitivity, or naevus count and type [1,4,5]. Mutations in the BRAF human proto-oncogene have been identified in 50% of malignant melanomas [6], and approximately 40–70% of the cases show a missense mutation, with a substitution of valine with glutamic acid at codon 600, named V^600E^ [7]. The identification of oncogenic driver mutations, such as KRAS and BRAF, has led to the development of small-molecule inhibitors along the RAS-RAF-MEK-MAPK signaling pathway [8].

MCM management comprises different treatments, including surgery, chemotherapy, immune checkpoint modulator therapy, and/or radiation therapy. Unfortunately, such methods are frequently not effective in the treatment of metastatic and advanced melanoma [9]. In fact, various features, including brain metastases, PD-L1 expression, BRAF mutation, performance status, and prior adjuvant therapy, significantly impact the direction of advanced melanoma treatment [10]. Recent approaches to treat late-stage MCM have focused on biomarkers that play significant roles in cell growth, proliferation, migration, and survival [9]. In this context, tyrosine kinase inhibitors (TKIs) are FDA-approved drugs able to act on BRAF, MEK, RAS, c-KIT, VEGFR, c-MET, and PI3K, whose overexpression is implicated in tumorigenesis [9]. The use of targeted systemic therapies as adjuvants to surgery when possible, or as an alternative or supplement to immunotherapy has revolutionized the management of metastatic melanoma [11].

Unfortunately, although the new treatment strategies are more efficacious and less toxic in comparison to traditional therapies, targeted therapies are less effective after prolonged treatment due to the acquisition of resistance [9]. Therefore, it is crucial to understand the mechanisms underlying the acquisition of resistance, as this condition occurs in most malignancies. For example, chemoresistance is often observed in neuroblastoma (NB), one of the most common extra-cranial pediatric solid tumors [12,13,14], which, like CM, originates from neural crest cells [15]. Indeed, although CM and NB arise at different ages, involve different tissues, and have different metastatic spread, in both cases, the prognosis for high-risk patients is still poor.

**Figure 1:**
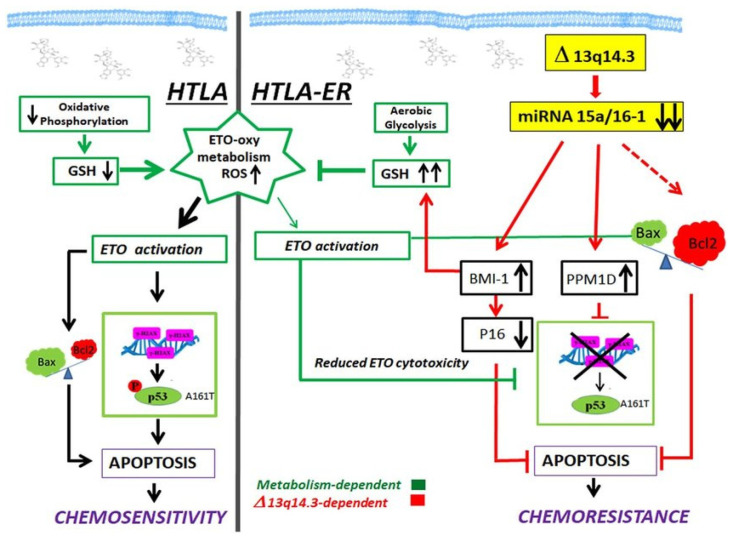
Molecular mechanisms underlying the chemoresistance of HTLA-ER cells. This Figure illustrates the observed molecular mechanisms underlying the chemoresistance of HTLA-ER cells and the events leading to apoptosis in ETO-sensitive HTLA parental cells. **Left panel:** Short-term treatment with ETO of HTLA-230 cells reduces oxidative phosphorylation and decreases glutathione (GSH) levels, inducing reactive oxygen species (ROS) overproduction, thus leading to DNA damage (H2AX). Consequently, ETO-induced genotoxic stress increases pro-apoptotic Bax, reduces anti-apoptotic Bcl2, and stimulates P53-Ser15 phosphorylation, all events leading to apoptosis and chemosensitivity. **Right panel:** HTLA-ER cells can efficiently counteract ETO-induced ROS production by maintaining an efficient aerobic metabolism and increasing GSH levels. Long-term treatment with ETO causes a deletion of the 13q14.3 locus and the consequent downregulation of miRNAs 15a/16-1, stimulating several pro-survival signals that contribute to inducing chemoresistance. This image was reproduced from an open-access article licenced under a Creative Commons Attribution 4.0 International Licence (http://creativecommons.org/licenses/by/4.0/, accessed on 24 March 2026), which permits use, sharing, adaptation, distribution, and reproduction in any medium or format, for any piece of content.

To shed light on the mechanisms involved in the acquisition of chemoresistance, we have previously selected and characterized both NB and CM cell lines resistant to commonly used drugs [16]. Regarding high-risk NB, it has been reported that its aggressive phenotype is strongly associated with the amplification of the MYCN proto-oncogene [17]. High-risk NB patients are currently treated with combined therapies including etoposide together with doxorubicin, cisplatin, vincristine, etc. However, although patients initially respond well to etoposide (ETO)-based therapy, which induces DNA damage and apoptosis through the inhibition of topoisomerase II and ROS production [16], most tumors relapse and acquire resistance. In this context, chronic ETO exposure has been shown to promote the development of multidrug resistance (MDR) in MYCN-amplified NB cells, enabling efficient DNA repair and escape from apoptosis. Mechanistically, prolonged ETO treatment induces genetic and epigenetic alterations, including deletion of the 13q14.3 locus, downregulation of miR-15a/16-1, and subsequent upregulation of BMI-1, which supports redox homeostasis and metabolic adaptation in drug-resistant cells, as in Figure 1 [12]. Similarly, also in CM cells, the chronic exposure to PLX, a drug specifically used in CM patients with BRAF^V600E^ mutation, generated a chemoresistant population that adapts well to the drug by maintaining an efficient oxidative metabolism and upregulating antioxidant cell defences [18].

Overall, despite initial chemosensitivity, both high-risk NB and MCM invariably evolve toward a chemo-resistant phenotype that compromises the efficacy of current therapies. Therefore, there is an urgent clinical need for novel therapeutic strategies capable of overcoming resistance mechanisms, potentially through extra-genomic approaches that limit the adaptive evolution of cancer cells.

### The Rationale of the Study

Recently, we demonstrated for the first time the potent and, per se, quite selective anticancer effects of a bola-amphiphilic (BA) molecule, containing two triphenyl phosphonium (TPP) groups linked by a 12-C alkyl chain, namely BPPB [19,20,21,22]. Although BA compounds were previously applied for several other uses [21,22], other than as anticancer devices [23], the anticancer effects of QPSs containing the TPP group have been extensively demonstrated. Nevertheless, despite these promising properties, the development of new QPSs with enhanced anticancer activity and reduced toxicity to normal healthy cells, including erythrocytes, remains an active and unmet research need, which should be further developed [24,25,26,27]. These compounds attract scientists because the TPP ion bears a positive charge delocalized over three phenyl rings and stabilized by resonance [21,28,29], which promotes the first step of their mechanism of action. In fact, the currently recognized mechanism of action of QPSs consists of an initially selective electrostatic interaction with the highly negatively charged constituents of tumor cells’ cytoplasmic membrane [21,28,29], which is facilitated by the characteristics of the TPP ion. Following the adhesion of TPP compounds to cancer cells, CPSs also possess the ability to penetrate the hydrophobic layers of the membrane, due to the overall lipophilic character of the cation. Not only can they enter the cancer cells, but they can also highly accumulate in their mitochondria, whose membrane potential is even more negative (−220 mV) than that of the mitochondria of normal ones (−160 mV), thus strongly attracting TPP molecules [30,31]. Once in the mitochondria, TPP molecules can interfere with mitochondrial functions, which represent the central point of the cell, inhibiting glycolysis, depolarizing the membrane potential, and inhibiting the mitochondrial permeability transition pore, thus leading to mitochondrial disruption in cancer. These events kill cancer cells mainly by apoptotic death, thus making QPSs and engineering mitochondrial-targeting therapeutic agents [31]. Mitochondria are, in fact, one of the most significant organelles in the intracellular environment because of their crucial role in energy metabolism and in the regulation of apoptosis processes [32]. The high affinity of TPP-containing QPSs for mitochondria has been confirmed by studies that have demonstrated their high affinity also for Gram-positive and Gram-negative bacterial superbugs [28,29], which, as is known, are their ancient parents, from which mitochondria have evolved [33]. In this context, after having demonstrated the potent antibacterial effects of a mono-TPP-containing compound on Gram-positive MDR isolates and those of BPPB vesicles cited previously on both Gram-positive and Gram-negative superbugs [28,29], a potent anticancer activity, perhaps mediated by interactions with mitochondria, was also demonstrated for BPPB. Specifically, an early and late apoptotic mechanism of action was observed for BPPB against neuroblastoma (NB) cells sensitive to ETO and multidrug-resistant (MDR) NB cells [20,22]. Moreover, BPPB anticancer effects were demonstrated against both BRAF mutant MeOV (BRAF^V600E^) and MeTRAV (BRAF^V600D^) metastatic cutaneous melanoma (MCM) cell lines sensitive to vemurafenib (PLX4032), and their PLX-resistant counterpart [19,21]. Practically, BPPB was active on different cancer cells regardless of their resistance, thus confirming the extra-genomic mechanism of action of TPP compounds, based on mitochondria function impairment, which overcomes the cells’ mutational mechanisms, thus overpowering the developed resistance and limiting its new emergence. No less important, our TPP-containing compounds demonstrated low levels of toxicity vs. different mammalian cell lines, primary neurons and astrocytes, and low haemolysis in red blood cells (RBCs), thus confirming their selectivity for both bacteria and tumor cells. With this evidence, we decided that one of the compounds to synthesize (compound **1**) should contain the TPP group. Also, despite the tri-tert-butyl groups used by Ermolaev et al. [34], which had exceptional antibacterial outcomes like those of TPP compounds previously reported, the TPP group was preferred because it was easier to achieve. Anyway, no alkyl chain was wanted, to differentiate **1** from the mostly already reported TPP compounds, and the benzyl group was used in place of the alkyl chain, to achieve a quaternized phosphorous salt (QPS). For the same reason, the benzyl group should have characterized the other compounds, which, anyway, should have contained the alkyl chains, but not the TPP group, to investigate if also diphenyl compounds could possess anticancer activity. In fact, compounds **3** and **4** should have contained different alkyl chains encompassing the same number of carbon atoms, but with different hydrophilic–hydrophobic balances. The chain of **3** was terminated with a primary alcohol group, thus being more hydrophilic than that of **4**, which terminated with a vinyl group. The TPP group was replaced by two phenyl rings plus the benzyl group, thus having the three benzyl derivatives wanted. Collectively, no designed compound should have been contained simultaneously in the TPP group and the alkyl chain, as for the most active compounds previously reported [28,29,35], to verify if the simultaneous presence of the TPP group and of the alkyl chain is strictly necessary for having consistent biological effects. The first choice of having the benzyl group in the structure of desired phosphonium salts depended on its high reactivity in nucleophilic substitutions S_N_1 with weak bases, such as the phosphorous atoms of phosphine precursors used here, thus assuring success also in the case of strong steric hindrance. Anyway, the maintenance of this choice was due to the antibacterial effects of benzyl phosphonium salts previously demonstrated by other authors [27,36,37,38], and to the consciousness that, in general, compounds that function as antibacterials are also effective as anticancer agents [39]. Moreover, Terekhova et al. reported that a series of benzyl phosphonium salts demonstrated mediocre-to-potent anticancer effects against several cancer cell lines [27].

## 2. Results and Discussion

### 2.1. Synthesis of Phosphine Compound ***2*** and Benzyl Phosphonium Salts ***1***, ***3*** and ***4***

**Chart 1:**
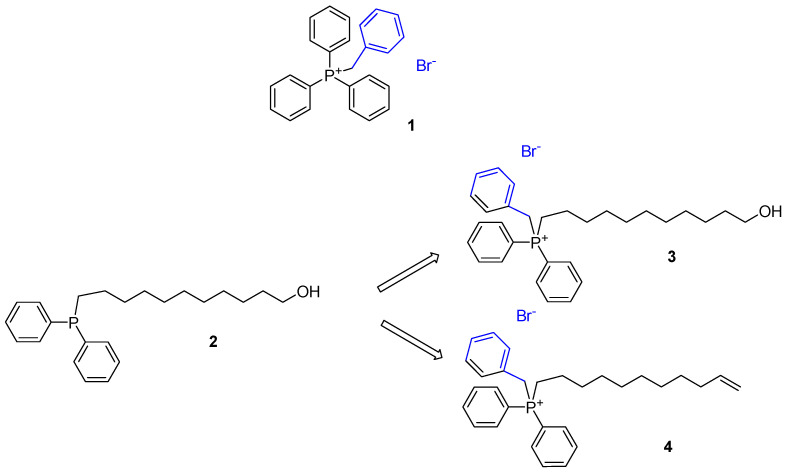
Chemical structure of compounds **1**–**4**. Non-quaternized compound **2** was prepared as precursor for achieving compounds **3** and **4**.

The aim of this study was to find novel compounds possessing cytotoxic effects against late-stage metastatic melanoma and neuroblastoma, as well as against tumor cells that have acquired resistance to currently available drugs such as vemurafenib (PLX) [18] and several other anticancer drugs (paclitaxel, doxorubicin, and etoposide (ETO)) [16]. In fact, as reported, such tumors initially well controlled with therapies based on PLX and ETO, due to prolonged high concentration and aggressive treatments, develop resistance to such drugs, inactivating the therapy, and thus limiting the patient’s life expectancy [16,18]. To this end, three quaternized phosphonium salts have been designed, following the rationale reported in the section “The Rationale of The Study”. Chart 1, reported below, shows their structures.

The non-quaternized uncharged phosphine derivative **2**, having a diphenyl-alkyl (11-undecanol chain) structure, was synthesized, since this is necessary to achieve compounds **3** and **4** (Chart 1).

#### 2.1.1. Benzyl Triphenyl Phosphonium Bromide **1**

**Scheme 1:**
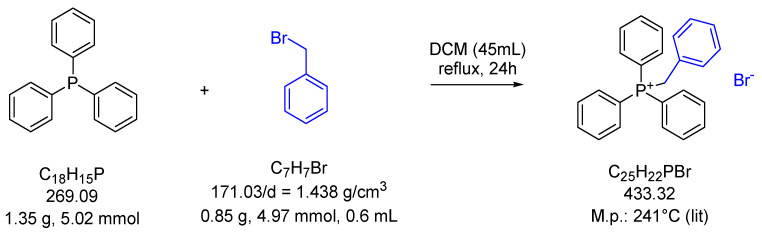
Synthetic procedure to prepare compound **1** [40]. DCM = dichloromethane.

Compound **1** is known [40] and was synthesized according to the procedure previously described by Cui et al. [40], with slight modifications in terms of solvent and times, according to Scheme 1.

Specifically, 1.01 equivalent excess of triphenyl phosphine (Ph_3_P) was reacted with benzyl bromide in DCM in place of toluene used by Cui et al., refluxing for 24 h instead of only 6 h [40]. The formation of a white precipitate was observed during the reaction, which increased on cooling. Diethyl ether (Et_2_O) was added to bring precipitation to completion. Compound **1** was isolated by filtration, obtaining a white solid with a high level of purity and no need for further purification, in 97% yield, outperforming the result by Cui et al. [40]. The melting point of **1** was very different from that reported by Cui et al., but like those reported by Cabiddu et al. and in an old patent published in 1959 [41,42]. The structure of **1** and its high level of purity were confirmed by ATR-FTIR, ^1^H, ^13^C, and ^31^P NMR, as well as by GC-MS and elemental analysis. Copies of the spectra of **1** are available in the section “The Rationale of the Study” (Supplementary Materials) as Figures S1.1–S1.4, while the related discussion and detailed peaks attribution are available under the images of each spectrum.

#### 2.1.2. 11-(Di-phenyl-phosphine)-1-undecanol (**2**) [43]

**Scheme 2:**
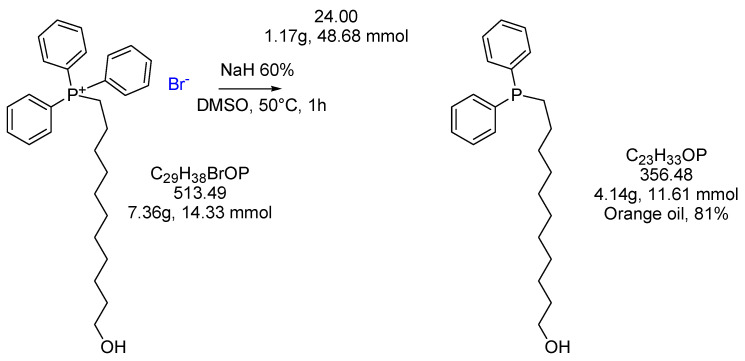
Synthetic procedure followed to prepare **2**.

Compound **2** is known. It can be prepared according to the procedure described by Petrucci and Kakkar via a three-step synthesis [44]. Specifically, authors prepared diphenyl alkanol derivatives with differently long alkyl chains (*n* = 10, 11, and 12), in very good yields, from the commercially available bromo alcohols. First, the alcohol groups were protected with a Me_3_Si group, and then the resulting Me_3_SiO-(CH_2_) n bromide compounds were reacted with KPPh_2_, followed by desilylation with citric acid [44]. In the case of **2**, the crude product was only washed, thus achieving the desired compound as a colorless oil in 96% yield. Anyway, Hands et al., in their previous study on the reactions occurring when sodium hydride is mixed with ω-hydroxyalkyl-phosphonium salts, reported that upon intimate mixing in a melt of 11-hydroxyundecyltriphenylphosphonium bromide and sodium hydride under heating at 120–140 °C (bath) in a stream of nitrogen for 45 min, a mixture of four compounds formed after hydrolysis with ammonium chloride, including 11-hydroxyundecyldiphenylphosphine **2** [43]. The compounds were separated by column chromatography on alumina, and the fraction eluted with benzene contained compound **2**, which was recrystallized from ether in low-melting prisms (52 °C) in 15% yield [43]. Despite the low yield, the reaction was carried out on a very large scale (30–60 g) and in a unique step, starting from the proper triphenyl phosphonium salt, which, in our case, was preparable in high yield from the proper commercial bromo alcohol without the need for sylanization [28]. On these considerations, **2** was here prepared following a procedure like that proposed by Hands et al., with substantial modifications in terms of reaction conditions and isolation work-up (Scheme 2).

**Scheme 3:**
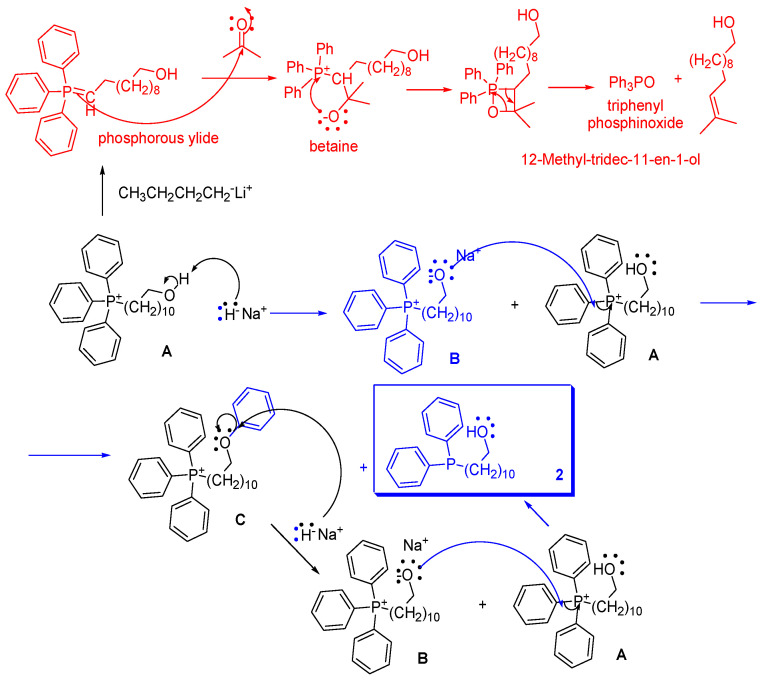
Mechanism proposed by Hands et al. and slightly modified to adapt it to new conditions and results for the formation of 11-DPPOH (**2**) via use of NaH (black/blue route) [43], and conventional employment of triphenyl phosphonium salts in Wittig reactions to prepare alkene derivatives via α-deprotonation with BuLi (red route).

In place of melting reagents and heat at 120–140 °C for 45 min, reagents were dissolved in DMSO and gently heated at 50 °C under stirring for 1 h. The presence of solvent and lower temperature promoted the formation of the desired product in place of that of the other products found by Hands et al., thus improving the overall yield. Then, silica in place of alumina was used for chromatography, eluting 11-DPPOH with DCM/MeOH 9/1. These changes allowed us to achieve 11-DPPOH with a high yield of 81%. In our conditions, the formation of 6-DPPOH (**2**) can be rationalized by assuming the initial deprotonation of the hydroxyl group by sodium hydride, providing a 13-atom betaine derivative (C-11 chain) (Scheme 3, black/blue route).

Note that this step occurs only if sodium hydride is used. Conversely, when butyl lithium is used as a deprotonation agent, the α-hydrogen to phosphorous atom is subtracted, providing a phosphorus ylide derivative usable in Wittig reactions with carbonyl compounds. Alkene derivatives, via betaine intermediates of the formation of four atoms and their cyclization, usually form (Scheme 3, red route). On the contrary, using sodium hydride, the 13-atom betaine formed, which, unlike cyclizing, made an aromatic nucleophile substitution on one of the phenyl rings of the starting still-unreacted compound A, as in an aldolic condensation reaction, thus providing 11-DPPOH (**2**), via nucleophilic aromatic substitution (Scheme 3, black/blue route). These events gave two products, including aromatic ether C and compound **2**. Following this, C entered a cyclic process, where, by subsequent nucleophilic aromatic substitution, C was re-transformed into betaine B, which, in turn, attacked unreacted A, thus providing further **2** and C, until total consumption of compound A. The structure of **2** and its high level of purity were confirmed by ATR-FTIR, ^1^H, ^13^C, and ^31^P NMR, as well as by GC-MS and elemental analysis. Copies of the spectra of **2** are available in the section “The Rationale of the Study” (Supplementary Materials) as Figures S1.5–S1.8, while a detailed discussion of spectra and peak attribution is available under the image of each spectrum.

#### 2.1.3. Benzyl-(11-hydroxy-undecyl)-diphenyl-phosphonium Bromide **3** and Benzyl-diphenyl-undec-10-enyl-phosphonium Bromide **4**

**Scheme 4:**
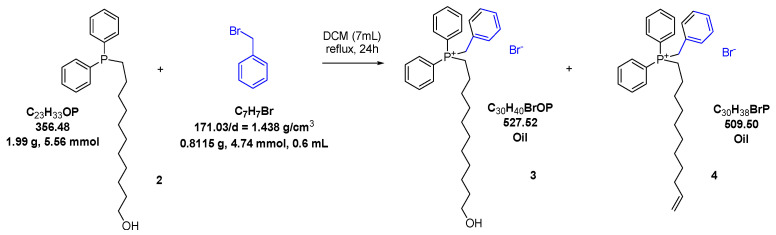
Synthetic procedure to achieve compounds **3** and **4**. DCM = dichloromethane.

Compound **3** was unexpectedly obtained as 1/1 mixture with compound **4**, following the procedure used to prepare compound **1**, but using a different phosphine reagent in a different excess (1:1.2) (Scheme 4).

**Scheme 5:**
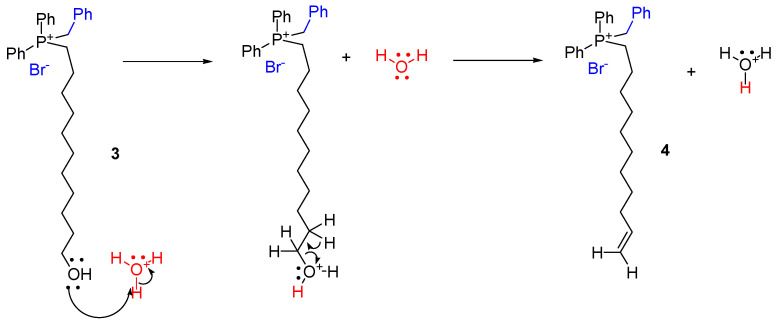
Mechanism of the E2 reaction by compound **3** to provide compound **4**.

Compounds **3** and **4** are unknown, and their structures were assigned based on the results from ^1^H and ^31^P NMR spectroscopy, chemometric-assisted ATR-FTIR, UV-Vis, and ^13^C NMR analyses, as well as from GC-MS and elemental analysis, which evidenced their high level of purity. Compounds **3** and **4** were separated thanks to their different solubility in Et_2_O, which was used to treat the crude oil under 24 h stirring, hoping for its solidification. Oil crystallization did not occur, but two phases were separated, containing two different compounds. The upper ether phase contained the dissolved compound later identified as **4**, while the oil that was not soluble in ether was then identified as the initially desired compound **3**. Anyway, the solubility of the first compound in ether, its TLC Rf (0.2581, MeOH/Petrol 1/1) being higher than that of a compound not soluble in ether (0.1828, MeOH/Petrol 1/1), and the different results obtained by the Schiff test, which was negative for the first soluble compound (later named **4**) and positive for the second insoluble one (later named **3**), could mean that the absence of the hydroxyl that was initially present is linked to the C-11 of the alkyl chain of **2** in the first, which was instead present in the second. The rational explanation of this early assumption consists of a partial acid-catalyzed thermal elimination of the OH group as water (E2 reaction) by the forming product **3**, to provide the final two compounds (**3** and **4**). Partial E2 is provided, no longer bearing the hydroxyl group compound **4**, having instead the C-10 double bond, resulting in a negative Schiff test, and compound **3**. Scheme 5 describes the mechanism of this E2 reaction when it occurs when forming product **3**.

Copies of the spectra of **3** are available in the section “The Rationale of the Study” (Supplementary Materials) as Figures S1.9–S1.12, while a detailed discussion of spectra and peak attribution is available under the image of each spectrum.

^1^H and ^13^C NMR spectra of **4** were like those of **3** with some substantial differences, due to the presence of a vinyl group. It gave peculiar signals in the NMR spectra. Copies of spectra of **4** are available in the section “The Rationale of the Study” (Supplementary Materials) as Figures S1.13–S1.16, while a detailed discussion of spectra and peak attribution is available under the image of each spectrum.

### 2.2. ATR-FTIR Spectra of Compounds ***1***–***4***

ATR-FTIR spectra of all samples were acquired as detailed in Section 3, and the lists of the most relevant bands have been reported in the characterization data of each compound. Copies of ATR-FTIR spectra of **1**–**4** are available in Section S2 of the Supplementary Materials in Figures S2.1–S2.4, while a detailed discussion of spectra and band attribution for compounds **2**, **3**, and **4** is available under the image of each related spectrum (Figures S2.2–S2.4). Bands observed in the ATR-FTIR spectra of each compound have been discussed, and band assignments to functional groups of **1**–**4** were made according to the Infrared Spectroscopy Absorption Table available online at https://chem.libretexts.org/Ancillary_Materials/Reference/Reference_Tables/Spectroscopic_Reference_Tables/Infrared_Spectroscopy_Absorption_Table (accessed on 22 November 2025) and literature reports [45,46,47]. In the following part of this Section, the particular and interesting case of compound **1** has been discussed. In the ATR-FTIR spectrum of compound **1**, weak bands at 3085, 3052, and 3010 cm^−1^ were due to the =C-H stretching of aromatic rings, while the aliphatic C-H stretching band of benzyl methylene, typically found at 2968 cm^−1^ in the form of a single band, was found as a double band, a band at 2859 cm^−1^, and a second band at lower wavenumber values (2773 cm^−1^). Ammer et al. reported that both bands were due to the so-called redshift, which caused the shifting of C-H stretching vibration to lower wavenumbers, giving two bands in the place of one [45]. The redshift is mostly the consequence of the hydrogen bond formation between a C-H proton atom acting as donor and an acceptor, and in the present case, the hydrogen bond between the C(α)-H of benzyl methylene and the ion pair of the bromide anion, as documented also by Arunan [46]. Note that the C(α)-H protons of benzyl triphenyl phosphonium salts are particularly acidic thanks to the stabilizing effects of the triphenyl phosphonium system and of the phenyl ring. Therefore, the formation of C(α)-H⋯X^−^ hydrogen bonds between the phosphonium ion and its counterion X^−^ is favoured [45]. Anyway, the hydrogen bond formation is more favourable in C(α)-H protons of benzyl derivatives, since they are more mobile, and its formation with the consequent redshift of the C(α)-H stretching bands has been reported even for alkyl triphenyl phosphonium halides, thus illustrating that hydrogen bonding also plays a role for substrates of lower C-H acidity. Particularly, a redshift was observed as a single band in the FTIR spectrum of *trans*-2-pentenyl triphenyl phosphonium bromide (2777 cm^−1^) reported by Bergerl’son [48] and in that of alkyl *bis*-triphenyl phosphonium compound (BPPB), where the C-H stretching band redshifted at 2794 cm^−1^ [29]. The redshift is explained considering that, when a hydrogen bond forms, promoted both by the acidity of C-H proton atoms and by the nature of H acceptor X^−^, strong acceptors such as Cl^−^ or Br^−^ promote strong attraction between H and X^−^, and the attractive interaction between the positive H and the negative X^−^ shortens the distance ⋯X^−^ and lengthens the C-H bond, thus reducing the force constant and leading to classical red-shifting [45]. Anyway, according to Ammer, in the spectra of benzyl derivatives used in their study, two redshifted bands were detected, which were the bands at 2859, and 2773 cm^−1^ found in **1**. Conversely, only one shifted band was visible in the spectra of alkyl triphenyl phosphonium derivatives. Ammer et al. attributed the occurrence of two bands in benzyl derivatives to possible couplings with lower-frequency modes, or with the existence of two conformations, but the authors were hindered by making assumptions that were too risky [45]. In our opinion, the two bands present at 2859, and 2773 cm^−1^ were due only to an incomplete redshift of the traditional C-H stretching band at 2868 cm^−1^, probably due to the steric hindrance of the phenyl ring, which partially impeded the hydrogen bond formation. In addition, as reported by Ammar et al., the acceptor Br^−^ can also form hydrogen bonds with the proton atom in *ortho* position in the phenyl rings of both the triphenyl phosphonium group and benzyl, thus reducing the C(α)-H⋯Br^−^ attraction interactions outside systems where only the phenyl rings of the TPP group exist. For the rest, the spectrum of **1** exhibited bands at 1456 and 1436 cm^−1^, as well as at 747, 718, and 689 cm^−1^, which were assigned to the C-H banding and the C-P stretching, respectively.

### 2.3. UV-Vis Spectra

The UV-Vis spectra of all compounds exhibited profiles like those of *bis*-triphenyl alkyl phosphonium compounds described in the literature [29,49]. Anyway, while diphenyl alkyl phosphine **2** and diphenyl benzyl alkyl phosphonium salts **3** and **4** presented three absorption peaks at 259, 265, and 271/72 nm and a shoulder peak at 253 nm, a bathochromic shift (redshift) to longer wavelengths (nm) was observed in the spectrum of **1**. Particularly, in the UV-Vis spectrum of **1**, the shoulder peak was no longer visible while the previous three peaks were observed at 261, 268, and 275 nm, as reported for BPPB [29] and by Ceccacci et al. [49] for similar compounds, which all showed the presence of the TPP group. This phenomenon is often caused by increased conjugation, solvent polarity effects, or the presence of auxochromes. In the case of TPP compounds, **1** conjugation is remarkably higher than in diphenyl compounds **2**, **3**, and **4**, thus justifying this difference in UV spectra. Copies of UV-Vis curves of compounds **1**–**4** are available in Section S3 of the Supplementary Materials as Figure S3.1a,b.

### 2.4. Principal Component Analysis (PCA) of ATR-FTIR UV-Vis and NMR Spectral Data

PCA is a multivariate chemometric technique frequently used to process datasets collecting very numerous correlated data (variables), most of which contain trivial information, to reduce them to a small number of non-correlated orthogonal variables (principal components), providing the most important information [50,51]. To carry out PCA, data must be arranged in a matrix of *n* columns × *n* rows as described in the Materials and Methods section [52]. In the following sections, we have reported and discussed the results presented as score plots, obtained by processing the matrices containing the ATR-FTIR, UV-Vis, and ^13^C NMR spectroscopic data of the considered samples by PCA. Since both ATR-FTIR and UV-Vis spectroscopic data regarded values of intensity (transmittance or absorbance), it was possible to merge them in an overall matrix containing data from both analytical techniques, which was processed by PCA too. In the score plots, scores are the new coordinates of the processed samples in the new space of the non-correlated PCs, where each sample assumes a position (score), depending on its chemical composition and structure [53]. Samples located close to each other share similar physicochemical characteristics, while those placed far apart could differ for different functional groups [53]. Note that, in PCA, principal components (PC1, PC2, PC3, etc.) do not have a fixed meaning valid for all datasets. PCs take on different meanings depending on the data type and structure (in our case, FTIR, NMR, or UV-Vis data), variables, and correlations. PCs are directions of maximum variance (intended as explained variance %, as shown in the following Figures), constructed specifically for that dataset. PCA works by finding new non-correlated coordinates (PCs), represented as x and y axes in the score plot. Considering PC1 (x axes) vs. PC2 (y axes), PC1 represents the direction along which the data variance is maximum, and PC2 represents that with maximum variance orthogonal to PC1, and so on. This means that each PC is a linear combination of the original variables specific to that dataset. Since each dataset has different correlations, different scales, different variances, and different structures, its PCs will be different and will have different meanings, and PCs will not represent the same “phenomenon” in different datasets.

#### 2.4.1. PCA of ATR-FTIR and UV-Vis Spectral Data

**Figure 2:**
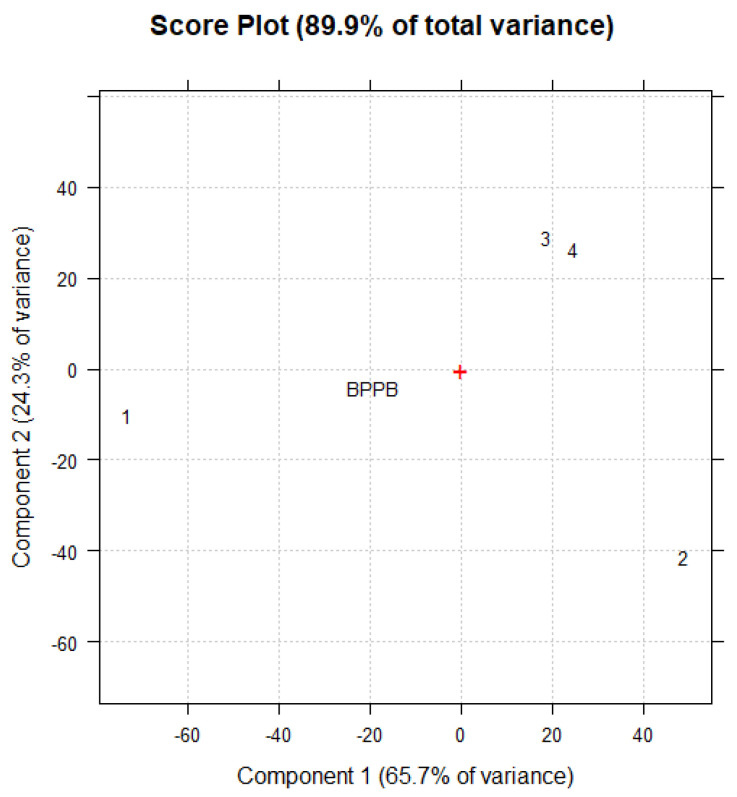
Score plot of PC1 vs. PC2 obtained by PCA, matrix E, made of the specific dataset containing variables related to UV-Vis and ATR-FTIR spectra of compounds **1**, **2**, **3**, **4**, and BPPB. PC1 vs. PC2 were selected for this dataset, since their orthogonal combination explained the max of total variance (89.9 vs. 100%), and they also provided the best separation and most useful information on the structures and chemical characteristics of the analyzed samples. The red cross indicates the PC1 intersection with PC2 at score zero.

Matrix A of 13,604 variables and matrix B of 17,005 variables, as well as matrix C of 364 variables and matrix D of 455 variables, where variables were the ATR-FTIR transmittance (%) values (matrix A and B) and the UV-Vis absorbance values (matric C and D) of each considered sample (compounds **1**, **2**, **3** and **4** in matrix A and C, while compounds **1**, **2**, **3** and **4** plus BPPB in matrix B and D), were constructed as described in the experimental section. Additionally, an overall matrix E of 17,460 variables, where variables were both the ATR-FTIR transmittance (%) values and the UV-Vis absorbance values of each considered sample, was also constructed. All matrices were subjected to PCA. The results from the PCA on all matrices, A, B, C, D, and E, have been reported as score plots explaining a total variance of 100%, 99.2%, 100%, 99.7%, and 98.99%, respectively. More specifically, PCA results obtained by processing all matrices were reported as score plots of PC1 vs. PC2. When matrix A was analyzed, PC1 explained 70.2% of total variance vs. PC2, which explained 28.3% (Figure S4.1a, in Section S4 of the Supplementary Materials), while in the case of matrix B, PC1 explained 65.8% of total variance vs. PC2, which explained 24.3% (Figure S4.1b, in Section S4 of the Supplementary Materials). Conversely, when matrix C and D were analyzed, PC1 vs. PC2 explained 78.0% vs. 21.7 and 77.3% vs. 20.21%, respectively, of total variance (Figure S4.1c,d in Section S4 of the Supplementary Materials). Concerning score plots obtained by PCAs on matrices containing either ATR_FTIR data and UV-Vis ones of compounds **1**–**4** (Figure S4.1a,c in Section S4 of Supplementary Materials), samples were separated on PC1, where compounds possessing the diphenyl phosphonium group and the C11 alkyl chain were all located in the right side of the plot at positive scores, while compound **1**, having the triphenyl phosphonium group and not the alkyl chain, was positioned on the extreme left side of the plot at negative scores. On the other hand, inside the diphenyl alkyl family, compounds were well separated also on PC2. Compound **2**, not having the benzyl group, was located distant from the compounds **3** and **4** bearing it, which appeared very close to each other. When FTIR data were processed, **2** was found at a negative score, whilst **3** and **4** were at similar positive scores, while when UV-Vis data were analyzed, it was the contrary. In this latter case, compound **3** was closer to compound **2** than **4** since they shared the presence of the CH_2_OH group that was no longer present in compound **4**, where a C=C double bond substituted the primary alcohol. When BPPB data were included in matrices B, D, locations of compounds **1**–**4** in score plots remained the same of those observed in the correspondent score plots without BPPB, while BPPB appeared at negative scores as **1** for chemical affinity (Figure S4.1b,d) When either ATR-FTIR and UV-Vis spectral data of compounds **1**–**4** and BPPB were processed (matrix E), locations identical to those observed in Figure S4.1b were observed for both compounds **1**–**4** and BPPB, which was positioned on the left side of the plot as **1** because BPPB shears with **1**, the triphenyl phosphonium group. Anyway, as already observed, BPPB positioned at less negative scores and closer to compounds **2**, **3**, and **4** because, differently from **1**, it contained a C12 alkyl chain like the C11 of diphenyl derivatives. To be noted, that in this case (matrix E), the scores observed in the score plot by PCA on ATR-FTIR data were maintained for **2**, **3** and **4**, thus evidencing that, when both ATR-FTIR and UV-Vis data were present simultaneously and analysed by PCA, the ATR-FTIR data had more weight in determining location of compounds in the score plot. The score plot of PC1 (65.7% of total variance explained) vs. PC2 (24.3% of total variance explained) obtained processing matrix E, is observable in Figure 2.

#### 2.4.2. PCA of NMR Spectral Data

**Figure 3:**
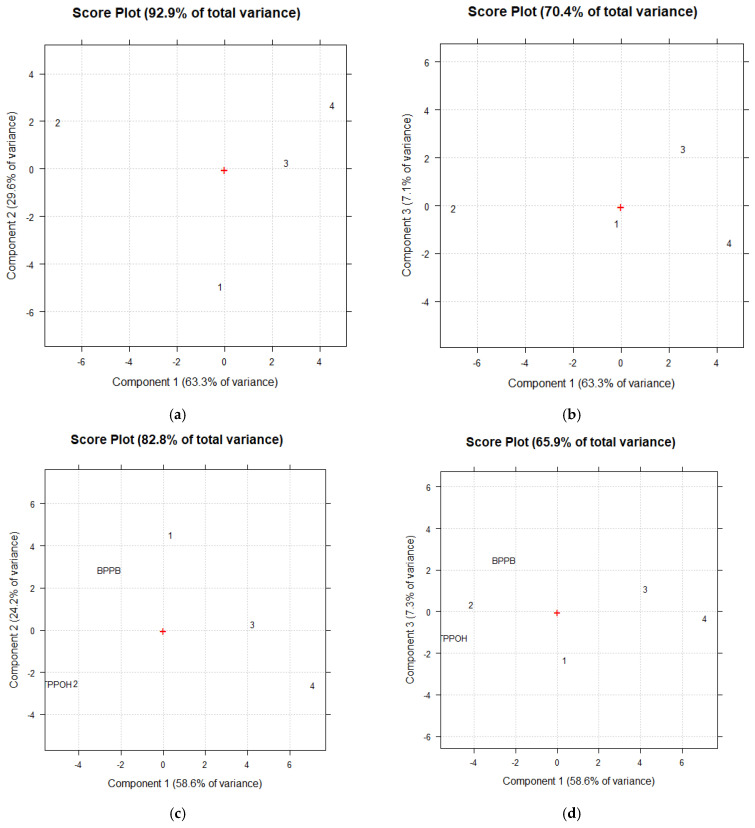
Score plots of PCA carried out on the matrix of 164 variables, consisting of the specific dataset containing variables related to the values of δ (ppm) of the NMR spectral data of compounds **1**–**4** expressed as PC1 vs. PC2 (**a**) and as PC1 vs. PC3 (**b**). Score plots of PCA carried out on the matrix of 246 variables consisting of the specific dataset containing variables related to the values of δ (ppm) of the NMR spectral data of compounds **1**–**4**, added with those of TPPOH and BPPB, expressed as PC1 vs. PC2 (**c**) and as PC1 vs. PC3 (**d**). PC1 vs. PC2 were selected for these datasets, since their orthogonal combination explained the max of total variance (92.9 and 82.8 vs. 100%), and they also provided the best separation and most useful information on the structures and chemical characteristics of analyzed samples on both PCs. Additionally, PC1 vs. PC3 score plots were shown for these datasets, since the substitution of PC2 with PC3, despite not explaining the max of total variance (70.4 and 65.9 vs. 100%), provided different separation views and useful adjunctive information on the structures and chemical characteristics of the analyzed samples. Red crosses indicate the PC1 intersection with PC2 or PC3 at score zero.

The ^1^H, ^13^C, and ^31^P NMR data of the considered samples were organized in matrices of 164 (compounds **1**–**4**) and 246 variables (**1**–**4** plus TPPOH and BPPB), constructed as described in the experimental Section 2, and were named A and B, respectively. The results from PCA on A and B have been reported as score plots of PC1 vs. PC2 and of PC1 vs. PC3. In the case of matrix A, the score plot of PC1 vs. PC2 explained the 92.9% of total variance (Figure 3a), where PC1 and PC2 explained the 63.3 and 29.6%, respectively. Conversely, the score plot of PC1 vs. PC3 explained the 70.4% of total variance (Figure 3b), subdivided in 63.3% (PC1) and 7.1% (PC2). In the case of matrix B, the score plot of PC1 vs. PC2 explained the 82.8% of total variance (Figure 3c), where PC1 and PC2 explained the 58.6% and 24.2%, respectively. On the contrary, the score plot of PC1 vs. PC3 explained the 65.9% of total variance (Figure 3d), subdivided in 58.6% (PC1) and 7.3% (PC3).

When NMR data of only compounds **1**–**4** were used, in the score plot of PC1 vs. PC2, the structural similarities and differences were well evidenced both on PC1 and PC2. On PC1, compounds with the benzyl group (**1**, **3**, and **4**) were on the right of the square plot, located from about zero to positive scores > 4, while compound **2**, which did not have this group, appeared on the left side at negative scores > −6. Conversely, PC2 separated compounds based on the presence or absence of the alkyl chain and TPP group. Compound **1**, lacking the alkyl chain and having the TPP group, was positioned in the lower part of the score plot at negative scores (about −5), while compounds without the TPP group and having the alkyl chain (**2**, **3**, and **4**) appeared in the upper part of the plot at scores in the range 0–3. When the score plot of PC1 vs. PC3 was observed, the structural similarities and differences were well evidenced only on PC1, where compounds appeared located as observed in the score plot previously considered, as expected.

When the NMR data of compounds **1**–**4**, added with those of TPPOH and BPPB, were used, in the score plot of PC1 vs. PC2, the structural similarities and differences were well evidenced both on PC1 and PC2. On PC1, compounds with the benzyl group (**1**, **3**, and **4**) were on the right of the square plot, located from about zero to positive scores > 6, while compound **2**, TPPOH, and BPPB without this group appeared on the left side at negative scores in the range 2–6 (absolute values). Conversely, despite separation based on structural characteristics making detection more difficult on PC2, a certain rationale can be noticed. Compound **2** and TPPOH were located at the same negative score on PC2 (about −2.5) because their structures differed only for one phenyl ring, while BPPB and **1** were located at positive scores based on the weight that aromatic components had in the structure. In these terms, compounds **1** and BPPB were the most aromatic ones. Only the separation of compounds **3** and **4** was curious and misleading, since **4**, in the place of **3**, was located at the same negative score as **2** and TPPOH, even though it is **3** that has the same undecanol chain as the two compounds. When the score plot of PC1 vs. PC3 was observed, the structural similarities and differences were well evidenced only on PC1, where compounds appeared located as observed in the score plot that was previously considered, as expected. On the contrary, the separation observable on PC3 was not rational.

### 2.5. Optical Microscopy Analyses

Bis-triphenyl phosphonium (TPP) compounds linked by a hydrophobic chain of carbon atoms, defined as bola-amphiphiles, when dispersed in aqueous solution at proper concentration, spontaneously self-assemble into spherical vesicles [29,49]. Such vesicles appeared micro-dimensional under the optical microscope and nano-dimensional at the DLS analysis [28]. In this case, although compounds **2**, **3**, and **4** do not possess the TPP groups, all compounds **1**, **2**, **3**, and **4** were dispersed in water and dissolved in methanol (MeOH) according to the procedure detailed in the experimental section and investigated using optical microscopy. The solutions were examined with a 40× and 100× objective, observing spherical polydisperse vesicles, for all samples except for compound **1**, which showed a tendency to crystallize on the glass for optical analysis when dissolved in MeOH and form macroscopic crystals. On the other hand, when suspended in water, compound **1** also demonstrated micro-dimensional irregular vesicles. Regardless of the objective used (40× or 100×), particles of **2**, **3**, and **4** appeared as polydisperse larger spherical vesicles or as smaller spheres of 1–14 µm. Sometimes larger spheres appeared, made of the smaller vesicles, better visible at 100× objective as already observed [29,49]. These findings evidenced that the capability to form vesicles in water spontaneously is not particular to bola-amphiphilic compounds but of amphiphilic compounds encompassing a cationic or non-cationic head not specifically made of a TPP group and an alkyl chain [49]. Figure S5.1 in Section S5 of the Supplementary Materials shows the spherical vesicles provided by **2**, **3**, and **4** as they appear when observed with the 40× and 100× objectives, as well as crystals (MeOH) or vesicles formed by **1**, as detailed in the Figure S5.1. caption. These findings confirmed that both compounds bearing cationic TPP heads and uncharged diphenyl (DP) ones, linked to hydrophobic chains, can form spherical vesicles of different sizes in water and MeOH, which can aggregate in larger ones depending on the solution concentration and coexist with them [29]. As reported, **2**, **3**, and **4** were probably able to self-assemble in spherical vesicles, due to their planar headgroups, because self-assembly properties are strongly dependent on the complex interplay of non-covalent interactions (ionic, hydrophobic, and π-π) inside the aggregate [29]. In this regard, the π-π stacking between the aromatic rings on the heads of compounds was crucial for the final aggregate morphology.

### 2.6. Biological Effects of Compounds ***1***, ***3***, and ***4*** on Tumoral and Non-Tumoral Human Cell Models

The acquisition of drug resistance is the major limitation of current anticancer therapies in both MCM and NB, severely compromising long-term treatment efficiency [54,55]. Currently, vemurafenib (PLX4032) is approved for the treatment of patients with BRAF^V600E^-mutated MCM [56,57] and, although with less effect, of those with BRAF^V600K^, BRAF^V600R^ and BRAF^V600D^ mutations [58].

However, despite the encouraging results obtained with the first treatments, chronic exposure to PLX4032 frequently leads to the development of resistant tumor cell populations, ultimately resulting in cancer relapse [59].

Similarly, in high-risk MYCN-amplified NB, prolonged treatment d with etoposide (ETO) promotes the selection of MDR cells characterized by an efficient aerobic metabolism and reinforced antioxidant defences [16].

Chasing the successful example of BPPB [19,20,21,22], three different QPSs, never tested as anticancer options, were here essayed against PLX-resistant MeOV and MeTRAV MCM cells, ETO-sensitive and MDR NB cells, human keratinocytes (HaCaT), and embryonic murine fibroblast (3T3) cells, as well as red blood cells (RBCs).

#### 2.6.1. Concentration- and Time-Dependent Anticancer Effects of **1**, **3**, and **4** on PLX-R MeOV, and MeTRAV Cell Viability

**Figure 4:**
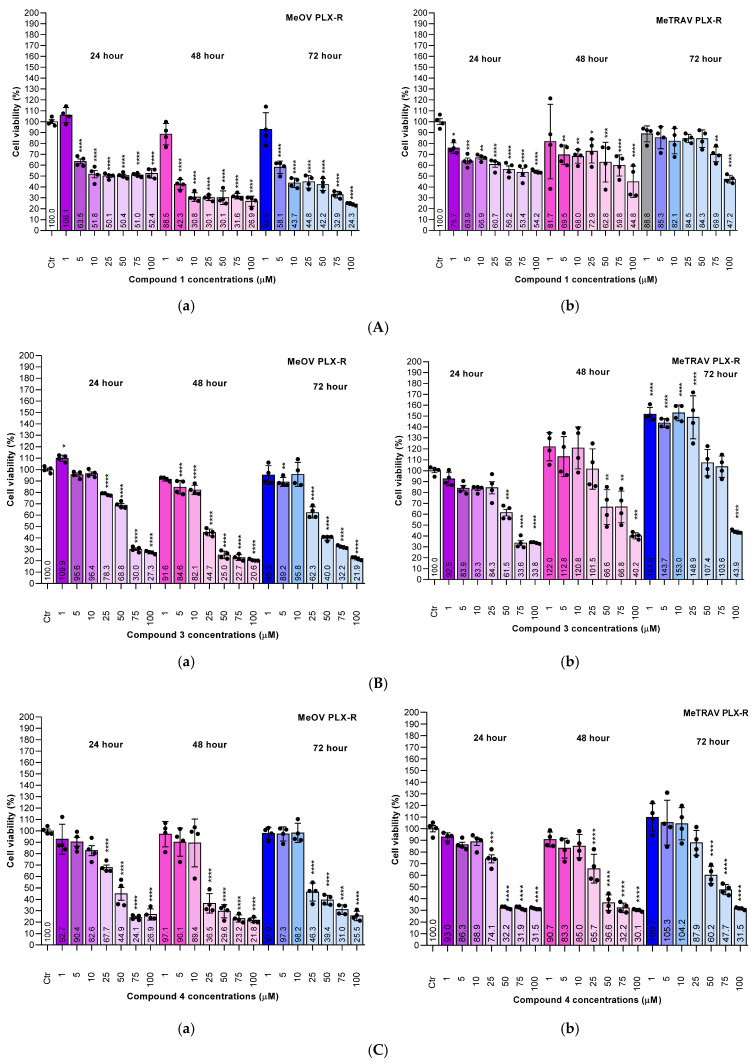
Cell viability was evaluated in PLX-R MeOV (**a**) and MeTRAV (**b**) MCM cells exposed to increasing concentrations of **1** (**A**), **3** (**B**), and **4** (**C**) (1–100 µM) for 24, 48, and 72 h. Bar graphs summarize quantitative data of the means ± S.D. of four independent experiments (black spheres) run in triplicate. Significance refers exclusively to control (Ctr) using the * symbol. Specifically, *p* > 0.05: no symbols; ****: *p* < 0.0001; ***: *p* < 0.001; **: *p* < 0.01; *: *p* < 0.05 (one-way ANOVA followed by Dunnett’s multi-comparisons test). Numbers within bars at the bottom indicate cell viability (%).

As shown in Figure 4 (images a, panels A, B, and C), all three tested compounds strongly reduced the viability of MeOV cells and, although MeTRAV was more refractory to treatments, its viability was also markedly reduced following administration of all compounds [19,21]. Compound **1** was the most effective in MeOV cells, especially at 48 h of exposure, where the cell viability rate was 42.3% and 30.8% already at 5 and 10 µM, respectively. In contrast, under the same experimental conditions (48 h; 5 and 10 µM), MeTRAV cells exhibited a viability rate of approximately 70%, indicating a less-pronounced cytotoxic effect compared to MeOV cells (Figure 4A(b)).

With respect to 24 h administrations, the tolerability of MeOV cells in longer treatments, with compounds **1** and **3**, remains mainly unchanged or slightly decreased, especially at the higher concentrations tested. Specifically, MeOV cell viability decreased from 52.4 to 26.9 to 24.3%, and from 27.3 to 20.6 and to 21.9%, going from 24 to 48 to 72 h treatments, when they were treated with **1** and **3**, respectively. Conversely, the tolerability of MeOV cells to compound **4** increased in the concentration range 1–10 µM, in 48 h treatments, and increased further in 72 h treatments up to 100 µM, with respect to 48 h treatments. Anyway, for concentrations ≥ 50 µM (24 h) and ≥ 25 (48/72 h), cell viability in MeOV cells decreased under 50% to only 26.9, 21.8, and 25.5%. As previously anticipated, despite being more tolerant than MeOV, MeTRAV cells’ viability was also remarkably reduced by the administration of all compounds, and especially **3** and **4** [19,21]. All compounds induced comparable cytotoxic effects in both cell populations at 24 h of treatment, although MeOV cells were slightly more sensitive (Figure 4). Here, the tolerability of MeTRAV cells to all compounds tended to augment under increased exposure times, and to a major extent in long-time 72 h treatments. Specifically, the tolerability of MeTRAV to **1** augmented up to a concentration of 75 µM, passing from 24 to 48 to 72 h exposure. MeTRAV tolerability to **3** augmented up to 100 µM concentrations, while tolerance to **4** decreased in 48 h treatments and increased in 72 h ones at all concentrations tested. Collectively, the phenomenon of increasing cell viability during longer treatments was less evident for **1** and more evident for **3**. In fact, despite cell viability (%) at the max concentration tested of **1** (54.2, 44.8, and 47.2%) being comparable, it increased up to concentrations of 75 µM when passing from 24 to 48 and to 72 h administrations (Figure 4B(a)). With **3** (Figure 4B(b)), all concentrations induced a significant increase in cell proliferation in 48 h treatment, and a cell viability under 50% (40%) was observed only at the max concentration tested (Figure 4B(b)). This trend became even more pronounced at 72 h, when cell viability at 75 µM concentration was still >100%, and it decreased to under 50% (44%) only at 100 µM. Although higher cytotoxic effects were exerted by **4** on MeTRAV, in 48 h treatments, a time-dependent behaviour like that of **3** was observed for this compound (Figure 4B(b)) in 72 h treatments. Specifically, in 48 h treatments, reduction in cell viability was like that observed in 24 h ones, indicating no substantial change in cytotoxic efficacy over time (Figure 4C(b)). At 72 h, like compound **3**, compound **4**, although less markedly, induced a partial recovery of cell proliferation in both cell lines and a parallel reduction in the cytotoxic effect (Figure 4C(a,b)).

The marked increase in PLX-R MeTRAV cell number also at sub-cytotoxic concentrations in longer-time administrations and the necessity to reach the max concentration tested to have the same viability (%) observed at lower concentrations in 24 h treatments suggest a biphasic dose–response pattern. This behaviour may be consistent with a hormetic-like effect, characterized by major low-dose stimulation and only high-dose inhibition in long-time treatments, a phenomenon described for several anticancer agents [60,61,62]. It is widely recognized that, in drug-resistant tumor cells (such as MeOV and MeTRAV), the initial apparent sensitivity observed after lower-time treatments (24 h) can significantly decrease within 48–72 h, especially at low concentrations [60,61,63]. This behaviour reflects the ability of resistant cells to progressively activate adaptive mechanisms—such as metabolic remodelling, drug persistence programs, and pro-survival signals—that typically emerge only after prolonged exposure [60,61,63,64]. Since this effect was not observed in PLX-R MeOV cells, intrinsic molecular features of PLX-R MeTRAV cells—possibly related to their phenotype, different from that of MeOV—may underlie this differential response. Studies demonstrated that MeTRAV, with respect to MeOV cells, exhibited significantly higher initial tolerance and activates more compensatory mechanisms during prolonged treatments [19,65]. Also, other findings indicate that MeTRAV has a tumor population that is more difficult to eradicate and that is characterized by more robust adaptive resistance than MeOV. In fact, although both MeOV (BRAF^V600E^) and MeTRAV (BRAF^V600D^) are PLX-resistant, independent studies report that melanomas bearing non-BRAF^V600E^ variants (such as MeTRAV) display poorer response to targeted inhibitors, enhanced tumor cell plasticity, and more complex adaptive resistance programs, making such tumor populations intrinsically more difficult to eradicate [66,67,68,69,70].

Anyway, further mechanistic investigations will be necessary to better clarify the pathways involved and to better define the therapeutic window of these compounds.

Note that the higher and significant anticancer activity of compound **1** against MeOV and of compounds **3** and **4** against MeTRAV cells was already evident after short-time treatments. Despite this event appearing as an adverse effect, in a hypothetical future in vivo administration, such rapid efficacy could reduce the need for long-time exposure to these compounds for each administration, thereby limiting the emergence of side effects, drug resistance, secondary tumorigenesis, and impact costs, while improving patients’ compliance and their quality of life.

**Figure 5:**
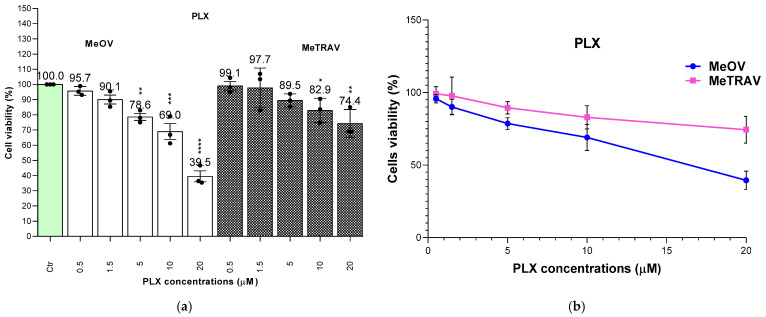
Cell viability (%) was evaluated in MeOV (white bars without pattern (**a**) and blue line (**b**)) and MeTRAV (white bars with squared pattern (**a**) and pink line (**b**)) PLX-R MCM cells exposed to increasing concentrations of PLX (0.5–20 µM) for 72 h. Bar graphs summarize quantitative data of the means ± S.D. of four independent experiments (black spheres) run in triplicate. Significance refers exclusively to control (Ctr, green bar) and is indicated with the * symbol. Specifically, *p* > 0.05: no symbols; ****: *p* < 0.0001; ***: *p* < 0.001; **: *p* < 0.01; *: *p* < 0.05 (one-way ANOVA followed by Dunnett’s multi-comparisons test). Numbers above bars indicate viable cells (%).

Notably, the max concentrations of our compounds **1**, **3**, and **4** used in the present study, expressed in µg/mL for comparison, were up to 5.8-, 4.3-, and 4.9-times lower, respectively, than those used by Li et al. against non-resistant metastatic B16F10 melanoma cells (250 µg/mL) [71]. Moreover, in the case of QPSs **1**, **3**, and **4**, NIH/3T3 viable cells (%) observed after administration of a 100 µM concentration of Sm^III^-EGCG for 24 h would correspond to 81.6, 85.96, and 85.1% of viable cells. In this regard, our compounds **1** and **4** were less cytotoxic than the compound by Li et al. against the same normal cells (3T3) by 2-fold, while the cytotoxicity of compound **3** was comparable (see later for details). IC_50_ values of compounds **1**, **3**, and **4** were calculated for both cell lines, based on cell viability data. For comparative purposes, cytotoxicity data of PLX4032, the first BRAF inhibitor to be tested in a phase III trial [72] against PLX-resistant MeOV and MetRAV cells, as previously determined [18], are reported in Figure 5, while the related IC_50_ values are reported in Table 1 together with those of all the QPSs tested against all MCM cells, as detailed in Section 3.

Collectively, the cytotoxic effects (IC_50_) of compounds **1** and **3** against PLX-R MeTRAV cells at 24 h exposure were comparable to those exerted by PLX after 72 h. In contrast, compound **4** at 24–48 h displayed higher cytotoxicity by 1.3–1.4 times than that exerted by PLX under 72 h-treatment [18]. Notably, in PLX-R MeOV cells, compound **1** was even more cytotoxic than PLX, showing 2.6- and 1.1-fold higher cytotoxicity after 48 and 72 h of treatment, respectively [18].

**Figure 6:**
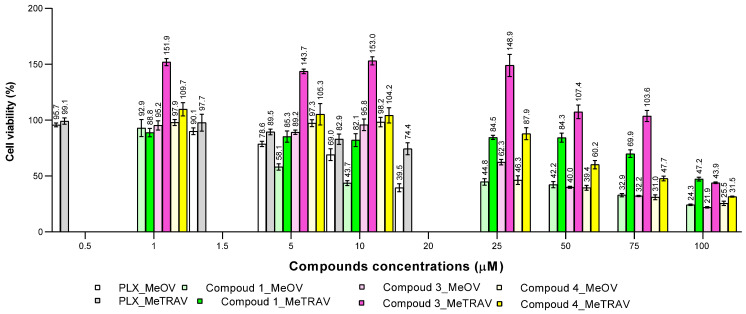
Cell viability of PLX-R MeOV and MeTRAV cells following treatment with compounds **1**, **3**, **4**, and PLX. MeOV (white bars) and MeTRAV (grey bars) cells were exposed for 72 h to PLX (0.5, 1.5, 5, 10, and 20 µM, white bars) or to compounds **1** (green bars), **3** (pink bars), and **4** (yellow bars) at concentrations of 1, 5, 10, 25, 50, 75, and 100 µM. Bar graphs summarize quantitative data of the means ± S.D. of four independent experiments (black spheres) run in triplicate. Numbers above bars indicate viable cells (%).

A comparison between PLX activity and that of our compounds after 72 h of exposure (due to the data available for PLX, [18]), in terms of residual viable cells (%), is shown in Figure 6.

Despite being administered at a lower concentration (1 µM versus 1.5 µM), compound **1** induced a greater reduction in MeTRAV cell viability than PLX. Similarly, at 5 and 10 µM (72 h), compound **1** showed higher cytotoxic efficacy than PLX in MeOV cells. Specifically, MeOV cell viability (%), following treatment with compound **1**, was 1.4 and 1.6-fold lower, respectively, compared to PLX-treated cells.

#### 2.6.2. Concentration- and Time-Dependent Effects of **1**, **3**, and **4** on HTLA 230 and MDR HTLA ER NB Cell Viability

**Figure 7:**
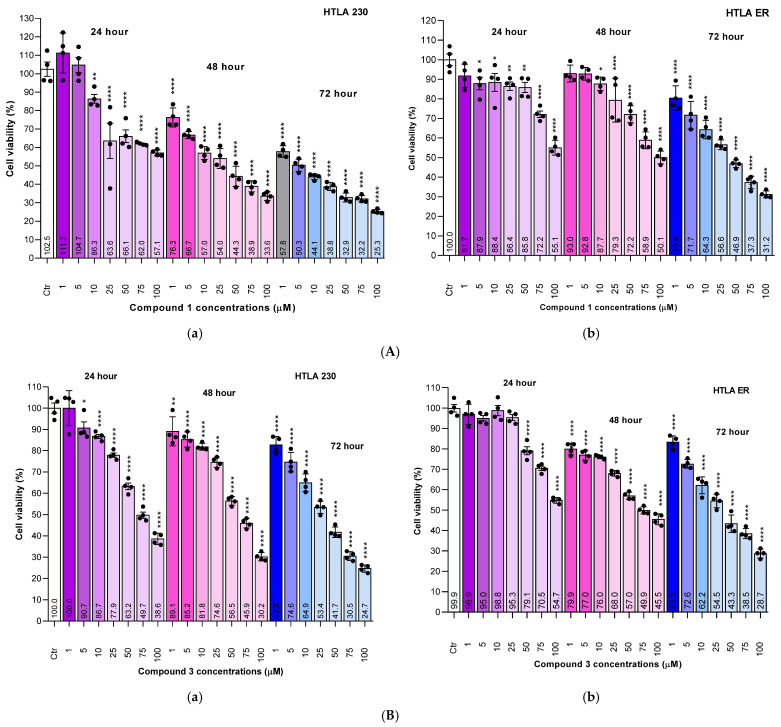

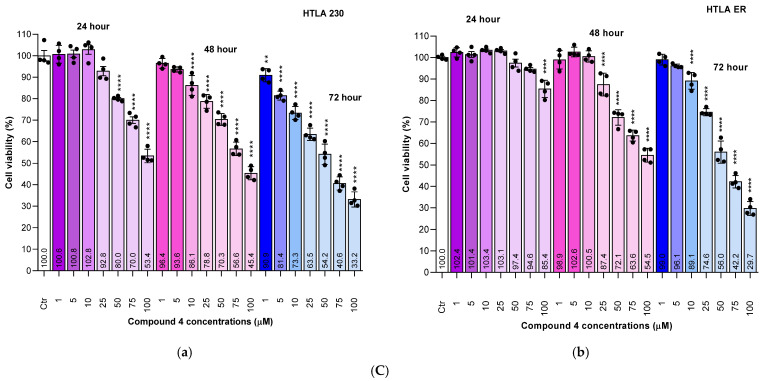
Cell viability was evaluated in HTLA 230 (**a**) and HTLA ER (**b**) neuroblastoma cells exposed for 24, 48, and 72 h to increasing concentrations (1–100 µM) of **1** (**A**), **3** (**B**), and **4** (**C**). Bar graphs summarize quantitative data of the means ± S.D. of four independent experiments (black spheres) run in triplicate. Significance refers exclusively to control (Ctr, white bars) and has been indicated using the * symbol. Specifically, *p* > 0.05: no symbols; ****: *p* < 0.0001; **: *p* < 0.01; *: *p* < 0.05 (one-way ANOVA followed by Dunnett’s multi-comparisons test). Numbers within bars at the bottom indicate viable cells (%).

As shown in Figure 7, all compounds induced concentration- and time-dependent effects in both ETO-sensitive (HTLA 230) and MDR (HTLA ER) NB cells. In general, 48 h treatments were more efficacious than 24 h, while 72 h treatments produced a further, albeit slight, increase in cytotoxicity compared with 48 h. Moreover, HTLA ER cells were more refractory to the treatments than HTLA 230 cells. Specifically, in HTLA 230 cells, compound **1** did not reduce cell viability below 50% at 24 h at any tested concentration. However, longer exposures significantly enhanced its cytotoxic effect, with viability decreasing to 44.3% and 44.1% after 48 and 72 h, respectively (50 and 10 µM). Conversely, in HTLA ER cells, compound **1** showed limited activity at 24 h, whereas after 72 h, viability was reduced to 46.9, 37.3, and 31.2% at 50, 75, and 100 µM, respectively. Compound **3** showed cytotoxic activity partially overlapping with, and in some conditions exceeding, that of compound **1,** depending on cell line and exposure time. In HTLA 230 cells, viability was reduced below 50% already after 24 (49.7%) and 48 h (45.9%) at 75 µM, and after 72 h at 50 µM (41.7%), indicating greater short-term efficacy compared to compound **1**. In HTLA ER, compound **3** did not reduce viability below 50% at 24 h at any tested concentration, similarly to compound **1**. After 48 h, cell viability decreased to 49.9 and 45.5%, at 75 and 100 µM, respectively. At 72 h exposure, viability further decreased to 43.3, 38.5, and 28.7%, at 50, 75, and 100 µM, respectively, indicating only a modestly higher efficacy than compound **1** in the MDR cell population. Compound **4** showed a cytotoxic effect broadly comparable to that of compound **1** but generally lower than that of compound **3**. In HTLA 230, compound **4** did not reduce cell viability under 50% after 24 h at any tested concentration; however, longer exposure reduced viability to 45.4% (48 h, 100 µM) and to 40.6 and 33.2% (72 h, 75 and 100 µM, respectively). In HTLA ER cells, compound **4** did not reduce viability below 50% at 24 and 48 h. Conversely, after 72 h, viability decreased to 42.2 and 29.7% at 75 and 100 µM, respectively. Collectively, compound **3** demonstrated the highest cytotoxic activity in both NB cell populations, whereas compounds **1** and **4** showed comparable but overall less-pronounced effects, particularly in the HTLA ER cells.

**Figure 8:**
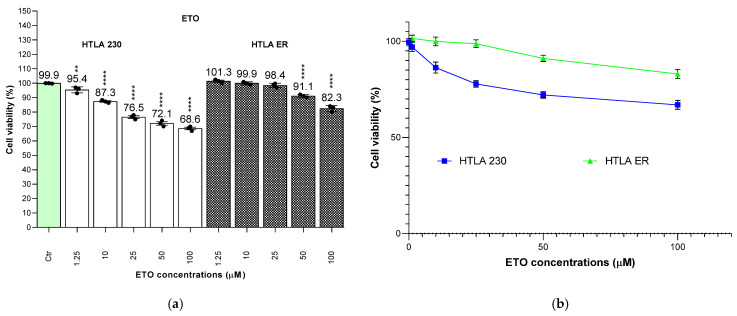
Cell viability of HTLA 230 (white bars without pattern (**a**) and blue line (**b**)) and HTLA ER (white bars with squared pattern (**a**) and green line (**b**)) after 24 h exposure to increasing concentrations of ETO (1.25–100 µM). Cell viability of cells observed at 0 µM concentration was assumed as the control (Ctr, green bar). Bar graphs summarize quantitative data of the means ± S.D. of four independent experiments (black spheres) run in triplicate. Significance refers exclusively to control (Ctr) and was reported using the * symbol. Specifically, *p* > 0.05: no symbols; ****: *p* < 0.0001; **: *p* < 0.01 (one-way ANOVA followed by Dunnett’s multi-comparisons test). Numbers above bars indicate viable cells (%).

Following the same procedure described for MCM cells, IC_50_ values of compounds **1**, **3**, and **4** were calculated for HTLA 230 and HTLA ER cells after 24, 48, and 72 h of treatment. For comparison with a clinically relevant therapy [72], previously published cytotoxicity data for etoposide (ETO) [72] in HTLA 230 and HTLA ER cells after 24 h of exposure [18] have been reported in Figure 8. Figure 8a shows the bar graph of cell viability (%) vs. increasing ETO concentrations (1.25–100 µM), while Figure 8b shows the corresponding dispersion graphs. IC_50_ values of ETO were calculated following the same method applied to our compounds and summarized in Table 2.

Although the activity of compounds **1**, **3**, and **4** against MDR HTLA ER may appear moderate when considered alone, it becomes highly relevant when compared with that of ETO, one of the most widely used drugs for the treatment of high-risk NB. Based on IC_50_ values, QPSs **1**, **3**, and **4** were more effective than ETO in both NB cell populations. At 24 h of exposure, compound **1** was 1.2-times more cytotoxic than ETO in HTLA 230 and 3.2-times more cytotoxic in HTLA ER cells. Compounds **3** and **4** showed even higher potency, being 2.0- and 1.3-fold more effective than ETO in HTLA 230 and 4.7- and 3.2-fold in ER, respectively.

**Figure 9:**
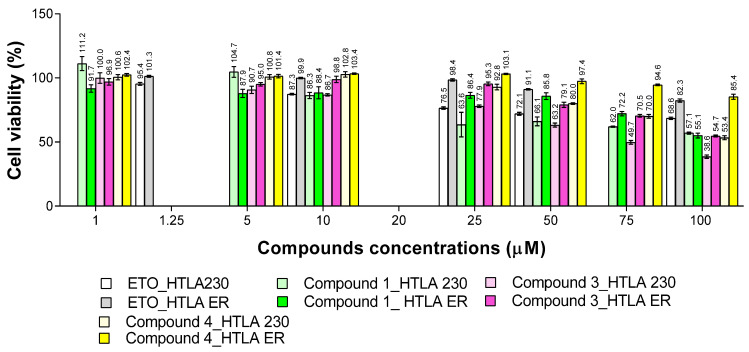
Cell viability was evaluated in HTLA 230 (bars in softer colors or white) and HTLA ER (bars in darker colors or grey) NB cells, at 24 h of exposure to 1.25–100.0 µM ETO and to 1.0–100.0 µM concentrations of **1** (green)**, 3** (pink), and **4** (yellow bars). Bar graphs summarize quantitative data of the means ± S.D. of four independent experiments, run in triplicate. Numbers above bars indicate viable cells (%).

A direct comparison of ETO (24 h exposure, the only data available for ETO) [18] with compounds **1**, **3**, and **4** at all concentrations tested (1–100 µM) has been shown in Figure 9.

**Figure 10:**
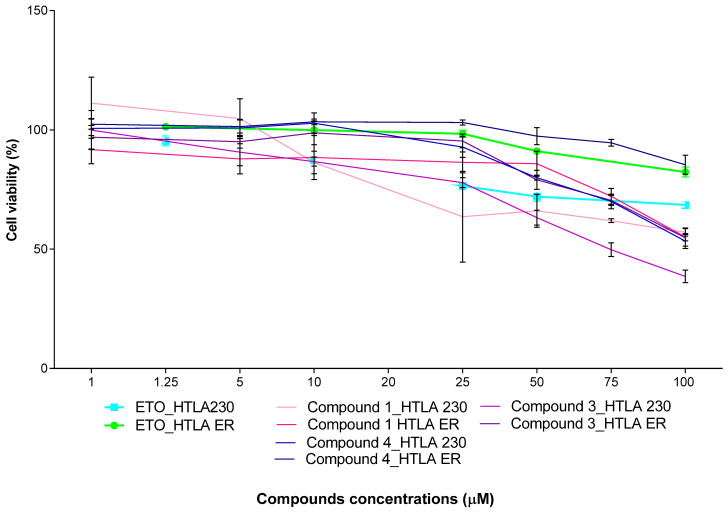
Dispersion graphs of cell viability of HTLA 230 and HTLA ER NB cells exposed to 1.25, 25, 50, and 100.0 µM concentrations of ETO (sky-blue and green lines with square and round indicators, respectively) and to 1, 5, 10, 25, 50, 75, and 100 µM concentrations of **1**, **3** and **4** (all other thinner lines without indicators) for 24 h.

At low concentrations, mainly compounds **1** and **3** exerted cytotoxic effects against HTLA ER cells. For example, at 1 µM, both compounds reduced cell viability to 91.7 (compound **1**) and 96.9% (compound **3**), whereas ETO at 1.25 µM slightly increased proliferation (101.3%). Similar effects were observed at 5 µM concentrations versus 10 µM of ETO, while both compounds administered at 10 µM were more potent than ETO administered at the same concentration against both HTLA 230 and ER. At higher concentrations (25- 100 µM), compound **1** was more efficient than ETO in both NB cells. In detail, compound **1** reduced HTLA 230 and HTLA ER cell viability to 63.6 and 86.4% at 25 µM, to 66.6 and 85.6% at 50 µM, and to 57.1 and 55.1% at 100 µM. Conversely, ETO reduced the viability of both NB populations to 76.5 and 98.4% at 25 µM, to 72.1 and 91.1% at 50 µM, and to 68.6 and 82.3% at 100 µM. Compound **3** showed a similar trend, being more effective than ETO at 50 and 100 µM, where it reduced HTLA 230 and HTLA ER viability to 63.8 and 79.9% (50 µM) and to 38.6 and 54.7% (100 µM), respectively, whereas ETO reduced cell viability to 72.9 and 91.9% at 50 µM and to 68.6 and 82.3% at 100 µM. Finally, compound **4** demonstrated superior efficacy than ETO at the highest concentration tested (100 µM), reducing HTLA 230 viability to 53.4% compared with the 68.6% observed for ETO, corresponding to an approximately 1.3-fold higher cytotoxic effect. Dispersion graphs illustrating cell viability (%) as a function of increasing concentrations of **1**, **3**, **4**, and ETO are reported in Figure 10.

At 100 µM, all compounds (**1**, **3**, and **4**) reduced the viability of both cell populations more effectively than ETO, demonstrating that the tested QPSs outperform ETO not only in MDR NB cells but also in HTLA230, which are considered sensitive to this clinically approved drug.

#### 2.6.3. In Vitro Hemolytic Toxicity of Compounds **1**, **3**, and **4** on Red Blood Cells (RBCs)

The hemolytic ratio percentage (%) caused by compounds **1**, **3**, and **4** was assessed as recently reported, but with slight changes [73]. Briefly, EDTA-blood samples from six healthy donors were exposed to increasing concentrations (0.5–100 µM) of each compound, while untreated blood (0.0 µM) was used as a control (CTR).

**Figure 11:**
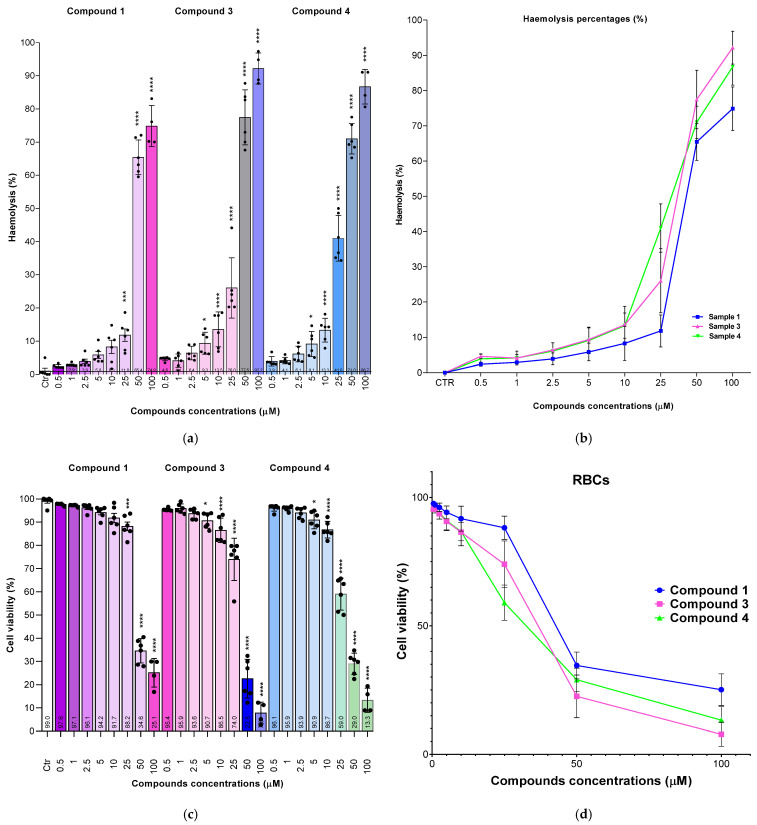
Hemolytic activity and red blood cell viability following treatment with compounds **1**, **3**, and **4**. Hemolytic ratio percentage (%) (**a**,**b**) and RBC viability (%) (**c**,**d**) are shown as a bar graph (**a**,**c**) and as corresponding dispersion graphs (**b**,**d**). Hemolysis (%) was determined by a recently reported protocol that was slightly modified [73]. Untreated RBCs were used as controls (Ctr, white bars). Cells were exposed to increasing concentrations (0.5–100.0 µM) of compounds **1**, **3**, and **4**. Experimental data are expressed as the mean ± S.D. of the data obtained from blood from six healthy donors. Significance is indicated exclusively vs. control (Ctr) as follows: no symbols: *p* > 0.05, and *: *p* < 0.1, ***: *p* < 0.001, and ****: *p* < 0.0001 (one-way ANOVA followed by Dunnett’s multi-comparisons test). In dispersion graphs, sample **1** is represented by blue lines, **3** by pink lines, and **4** by green lines.

Hemolysis (%) and cell viability (%) are shown in Figure 11a,c, while in Figure 11b,d, the corresponding dispersion graphs are reported.

**Figure 12:**
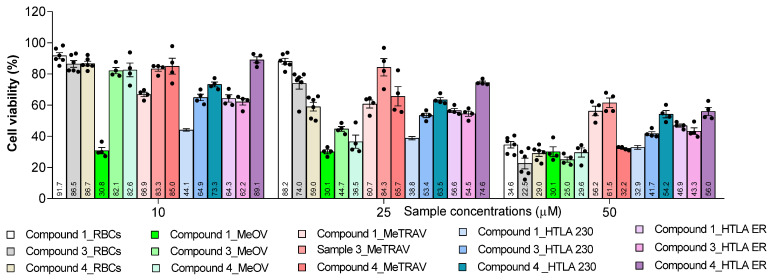
Cell viability (%) was evaluated in RBCs (white-to-light-grey bars group), MeOV (green bars group), and MeTRAV (pink bars group) MCM cells, as well as in HTLA 230 (soft blue bars group) and HTLA ER (violet bars group) NB cells exposed to 10, 25, and 50 µM concentrations of **1** (first bar in all groups), **3** (second bar in all groups), and **4** (third bar in all groups). Each bar summarizes quantitative data of the means ± S.D. of independent experiments (black spheres) run in triplicate. Numbers at the bottom within the bars indicate viable cells (%).

As shown in Figure 11a,c, hemolysis (%) and reduction in RBC viability (%) were statistically significant at concentrations ≥ 20 µM for compound **1** and ≥5 µM for compounds **3** and **4**. However, substantial hemolysis and marked loss of cell viability (below 50%) were observed only at concentrations ≥ 50 µM, corresponding to hemolytic ratios of 65.4 (compound **1**), 77.5 (compound **3**), and 71.0% (compound **4**). The comparison between the viability (%) of RBCs and that of all cancer cells after more efficient treatment, in terms of time and exerted cytotoxicity, when exposed to 10, 25, and 50 µM of **1**, **3**, and **4** is shown in Figure 12.

**Figure 13:**
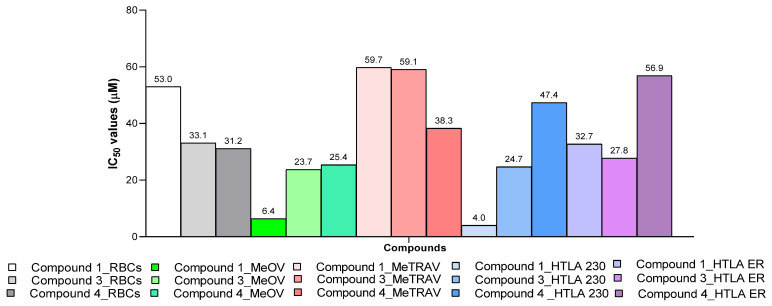
Comparison between the HC_50_ values of compounds **1**, **3**, and **4** vs. RBCs and their IC_50_ values vs. all cancer cells, obtained considering the more efficient treatment in terms of exposure time and cytotoxic effects. Numbers above bars indicate the IC_50_ values.

As shown in Figure 12, at 10 µM concentrations, all compounds were generally more cytotoxic towards cancer cells than hemolytic towards RBCs, except for compound **4** against HTLA ER, where RBC viability (86.7%) was slightly lower than that of the cancer cells (89.1%). At 25 µM, this trend was maintained for most compounds, except for samples **3** and **4** against MeTRAV and for compound **4** against both HTLA 230 and ER NB cells. At 50 µM, in most cases, except for sporadic circumstances on MeOV cells, hemolytic effects exceeded cytotoxicity. This behaviour is particularly relevant for a possible systemic clinical use of these QPSs, as it has indicated a therapeutic window in which cancer cells are preferentially targeted while minimizing hemolysis. However, for their hypothetical topical application in metastatic melanoma to treat skin lesions, this limitation is less critical. Skin metastases often represent the first sign of advanced disease or of recurrence [74] and, while sometimes asymptomatic, in advanced stages they can develop ulceration, bleeding, and superinfection, and can cause symptoms related to compression on nearby tissues, severely impacting patient quality of life [74], which could be ameliorated by the topical administration of QPSs **1**, **3**, and **4**. Anyway, as previously described for the other viability experiments, the dispersion graph of compounds **1**, **3**, and **4** (concentrations vs. RBCs viability; Figure 11d) was used to calculate their HC_50_ values, defined as the concentrations required to induce 50% hemolysis. HC_50_ values for RBCs and IC_50_ values for all cancer cells at each time of exposure were reported in Table 3, and a comparative analysis between HC_50_ and the best IC_50_ values observed for each compound was shown in Figure 13.

According to the results shown in Table 3, in several cases, the HC_50_ of the QPSs versus RBCs were lower than their IC_50_ against cancer cells (seven cases for **1**, nine for **3**, and 11 for **4**), indicating a considerable hemolytic toxicity. However, when HC_50_ values were compared with IC_50_ obtained under the most effective exposure times (Figure 13), all compounds were less hemolytic than cytotoxic against PLX-R MeOV cells. Compounds **1** and **3** were also more cytotoxic than hemolytic against most other cells, except for the PLX-R MeTRAV population. Conversely, compound **4**, already identified as the least active anticancer QPS in this study, was generally more hemolytic than cytotoxic, except for PLX-R MeOV cells, indicating a lower selectivity for tumor cells.

#### 2.6.4. Concentration- and Time-Dependent Effects of Samples **1**, **3**, and **4** on HaCaT and 3T3 Cell Viability

**Figure 14:**
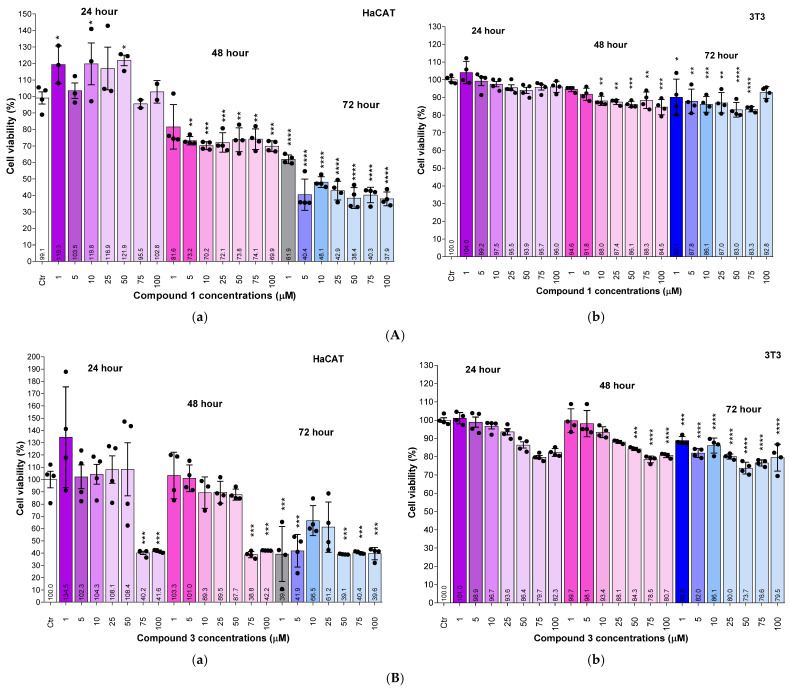

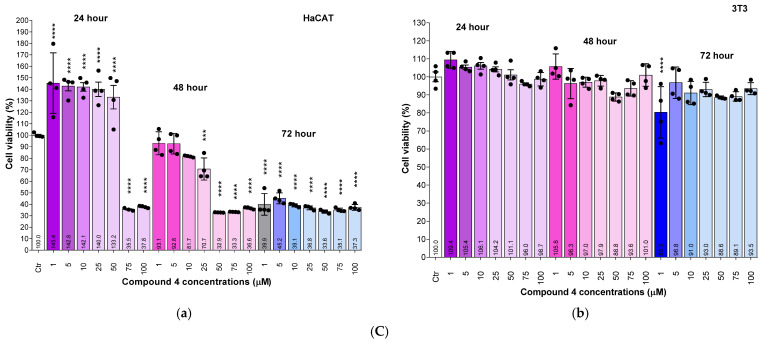
Cell viability was evaluated in HaCaT (**a**) and 3T3 (**b**) cells when exposed to increasing concentrations of **1** (**A**), **3** (**B**), and **4** (**C**) (1–100 µM) for 24, 48, and 72 h. Bar graphs summarize quantitative data of the means ± S.D. of four independent experiments (black spheres) run in triplicate. Significance refers exclusively to control (Ctr, white bars) and was indicated using the * symbol. Specifically, *p* > 0.05: no symbols; ****: *p* < 0.0001; ***: *p* < 0.001 (0.0002); **: *p* < 0.01; *: *p* < 0.05 (one-way ANOVA followed by Dunnett’s multi-comparisons test). Numbers within bars at the bottom indicate viable cells (%).

To further investigate the potential clinical development of compounds **1**, **3**, and **4**, either as a topical drug for the treatment of resistant melanoma skin lesions or as components of chemotherapeutic regimens targeting high-risk resistant NB, their effects were evaluated in non-cancerogenic cells. Specifically, HaCaT cells, an immortalized non-cancerous human keratinocyte cell line [75,76,77,78], and 3T3 cells, a fibroblast cell line derived from mouse embryonic tissue, were treated with compounds **1**, **3**, and **4** (1–100 µM) for 24, 48, and 72 h, and cell viability was assessed (Figure 14).

**Figure 15:**
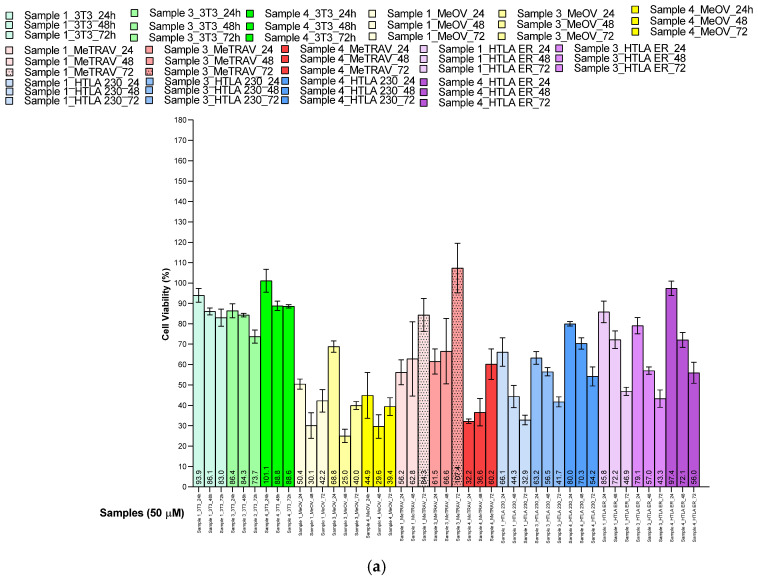

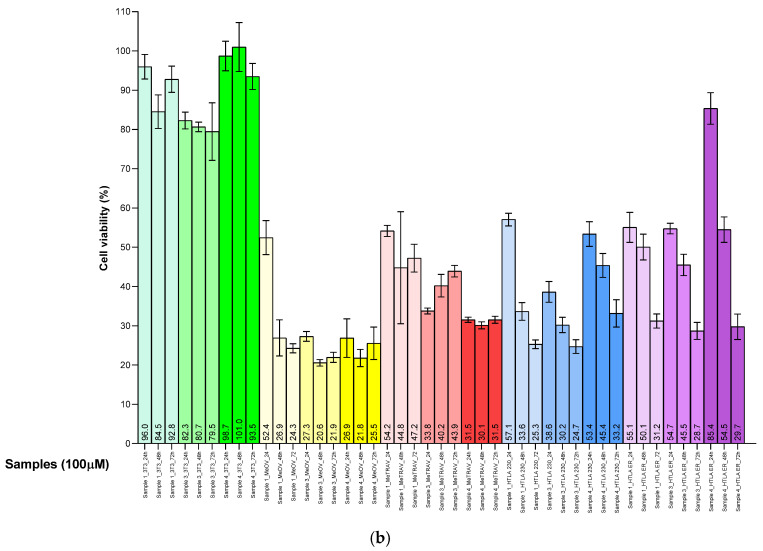
(**a**) Bar graph comparing the average cell viability (%) of 3T3 fibroblasts and that of MeOV, MeTRAV, HTLA 230, and HTLA ER cells after exposure to 50 µM (**a**) and 100 µM (**b**) of samples **1**, **3**, and **4** for 24, 48, and 72 h. Experimental data are expressed as the mean of four independent experiments ± S.D. made in triplicate. Numbers at the bottom within the bars indicate cell viability (%). The legend for Figure 15b is the same of that for Figure 15a. Collectively, in Figure 15, we can observe three bars for every sample and nine bars for every cell line.

The 3T3 cells were markedly more refractory to treatment than HaCaT cells, and all compounds displayed very low toxicity toward this fibroblast cell line. Among them, compound **3** showed slightly higher cytotoxicity; however, cell viability remained high (~81–91%), depending on exposure time (Figure 14). Even under the most aggressive conditions (72 h, 100 µM), cell viability exceeded 92% (compound **1**) and 93% (compound **4**), while treatment with compound **3** resulted in 79.5% viable cells. Conversely, HaCaT cells displayed greater sensitivity, especially at concentrations > 50 µM and after 48 and 72 h exposure. Compound **1** did not exert cytotoxic effects at 24 h and even induced a slight increase in proliferation (102.8% at 100 µM). After 48 h treatment, only a slight cytotoxicity was observed (69.9% viable cells), whereas a marked reduction in cell viability was observed at 72 h, when viability decreased to 37.9% at 100 µM. Compound **3** behaved similarly to compound **1** in 24 h treatment, whereas at 48 and 72 h, its effects were comparable to those of compound **4**. Specifically, at 48 h, compounds **3** and **4** reduced HaCaT viability to 38.8% (75 µM) and 32.9% (50 µM), respectively. After 72 h, viability decreased to 39.1% at 50 µM (compound **3**) and at 10 µM (compound **4**). A graphical comparison of the average cell viability (%) of 3T3 fibroblasts and cancer cell lines (MeOV, MeTRAV, HTLA 230, and HTLA ER) after exposure to 50 (Figure 15a) and 100 µM (Figure 15b) of compounds **1**, **3**, and **4** for 24, 48, and 72 h is shown in Figure 15.

**Figure 16:**
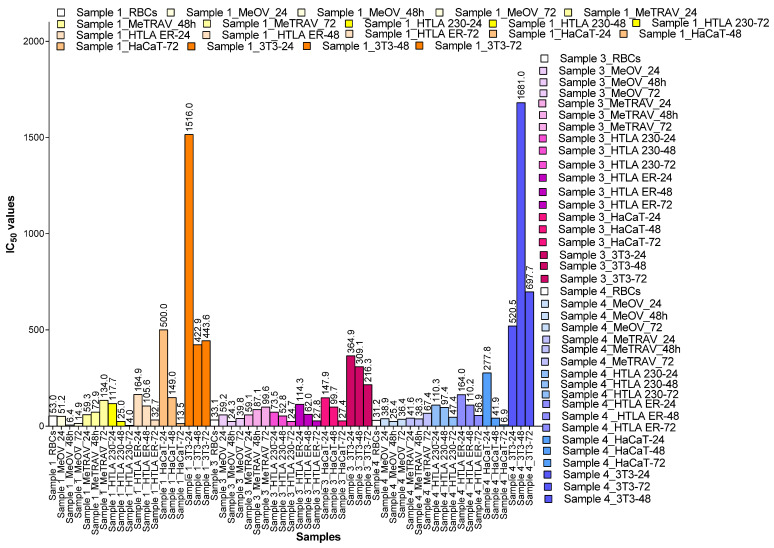
IC_50_ values of compound **1** (white-to-yellow–orange bars), **3** (white-to-pink–red bars), and **4** (white-to-soft blue bars up to dark-blue ones) against RBCs (white bars), HaCaT, 3T3 non-tumorigenic cells, and all MCM and NB cell lines (colored bars).

Curiously, the average viability of 3T3 cells (89.9%) treated with all compounds at 100 µM was slightly higher than that observed at 50 µM. Anyway, at 50 µM, only two cases out of 36 total experimental conditions, i.e., compounds **1** and **4** after 72 h exposure in PLX-R MeTRAV MCM cells, showed marginally greater cytotoxicity toward 3T3 cells than toward cancer cells. Conversely, in all other cases, 3T3 viability was consistently higher than that of all cancer cell lines under the same exposure conditions. When **1**, **3**, and **4** were administered at 100 µM, 3T3 cell viability was invariably higher than that observed in cancer cell lines. To enable a direct comparison between non-tumorigenic (HaCaT and 3T3) and cancer cells, IC_50_ values for HaCaT and 3T3 cells were calculated. For compound **4** in 3T3 cells at 24 h, the IC_50_ values were derived from the equation of the best-fitting curve, as the software flagged the standard calculation as unreliable. The IC_50_ values of all compounds for MCM and NB cell lines have been reported in Figure 16.

Figure 16 confirmed that, for compounds **1** and **3**, PLX-resistant MeTRAV cells were more refractory than MeOV. In NB models, sensitivity was exposure-time dependent, with HTLA ER cells generally less sensitive than HTLA 230 ones. On the contrary, compound **4** exhibited similar IC_50_ values across all tested cell lines (25.4 to 67.4 µM). HC_50_ values of all QPSs versus RBCs were generally high, indicating limited acute hemolysis. However, in several cases, HC_50_ values were lower than the corresponding IC_50_ values for cancer cells, indicating a certain level of time-dependent hemolytic toxicity. Importantly, IC_50_ values for 3T3 fibroblasts were significantly higher than those observed for cancer cells, especially for compounds **1** and **4**, confirming preferential cytotoxicity toward malignant cells. As previously reported, HaCaT cells were more sensitive than 3T3 cells, especially to compound **4**. QPSs-induced cytotoxicity in HaCat was clearly time-dependent: 24 h exposure was generally well-tolerated, whereas 72 h treatments produced a marked reduction in viability. Importantly, all newly developed QPSs were significantly less hemolytic than the previously reported BPPB, by factors of 3.4 (**1**), 2.1 (**3**), and 2.0 (**4**). Additionally, compound **1** was markedly less cytotoxic toward HaCaT than BPPB by 123.4- (24 h), 213.4- (48 h), and 14.6-fold (72 h). Similarly, compound **3** showed reduced cytotoxicity by 36.8- (24 h), 144.5- (48 h), and 29.75-fold (72 h), while **4** was less cytotoxic by 69.1- (24 h), 60.0- (48 h), and 7.5-fold (72 h).

Moreover, the cytotoxicity of all QPSs toward HaCaT cells was substantially lower than that reported for cationic dendrimer nanoparticles (PAMAM) of the fourth (G4), fifth (G5), and sixth (G6) generations, extensively reviewed for biomedical applications. According to the data reported by Mukherjee et al. after 24 h-exposure [78], and using the same MTT assay used in this study, compound **1** was 155.7-, 467.3-, and 490.2-fold less toxic than G4, G5, and G6 respectively, while compounds **3** and **4** by 46.07, 138.2, 145.0 and by 86.5, 259.6, and 272.4 times, respectively [78].

#### 2.6.5. Why Did HaCaT and 3T3 Cells Demonstrate a Substantial Different Sensitivity to QPSs **1**, **3**, and **4**?

Collectively, both non-tumorigenic cell lines tolerated QPSs **1**, **3**, and **4** reasonably, supporting the potential feasibility of both topical and systemic administration, particularly under short-time exposure conditions. However, the marked difference in sensitivity observed between HaCaT and 3T3 cells warrants an explanation. Keratinocytes are intrinsically more sensitive to xenobiotics and irritants than fibroblasts, as they represent the first cellular barrier against environmental insults and play an active role in cutaneous inflammatory and immune responses. Accordingly, they often show higher sensitivity to toxic chemicals compared with fibroblasts. A comparative toxicology study has shown that HaCaT keratinocytes generally respond more strongly to irritants than 3T3 fibroblasts, reflecting their physiological role as epidermal sentinel cells [79]. The differential sensitivity observed in the present study may also relate to the mechanism of action of QPSs. Many phosphonium-containing compounds, especially TPP-bearing QPSs such as compound **1,** are highly lipophilic cations that preferentially accumulate in the mitochondria due to the negative mitochondrial membrane potential. This accumulation can induce mitochondrial depolarization, enhance ROS generation, and trigger activation of apoptotic pathways [80,81]. This implies that cell types with higher mitochondrial activity or greater sensitivity to oxidative stress, such as keratinocytes, may therefore undergo cytotoxic responses earlier than fibroblasts. Moreover, keratinocytes have a stronger oxidative stress signaling profile than fibroblasts, which is part of their immunological sentinel role in the epidermis. Consequently, compounds capable of inducing mitochondrial dysfunction or ROS generation (such as QPSs) may produce more pronounced effects in epithelial cells than in fibroblast cells [79]. Collectively, it can be assumed that the higher sensitivity of HaCaT cells compared with 3T3 fibroblasts can be based on: (i) keratinocytes’ inherently higher sensitivity to toxic chemicals and their frontline role in responding to irritants [79]; (ii) mitochondrial targeting by QPSs leading to membrane polarization and oxidative stress [80,81]; and (iii) cell type-dependent susceptibility in stress-response signaling, which is generally higher in epithelial cells (like HaCaT) than fibroblasts, as in [79]. Importantly, despite this differential sensitivity, fibroblasts remained largely unaffected, even at high concentrations, supporting a degree of selectivity that may be advantageous for therapeutic development.

#### 2.6.6. Selectivity Index

The selectivity index (SI) of all QPSs for cancer cells in relation to RBCs, HaCaT, and 3T3 cells was calculated to predict their therapeutic potential. Generally, the SI values against tumoral cells (TCs) in relation to non-tumoral cells (NTCs) are calculated using Equation (1):

SI = IC_50_ for NTCs/IC_50_ for TCs (1)

**Figure 17:**
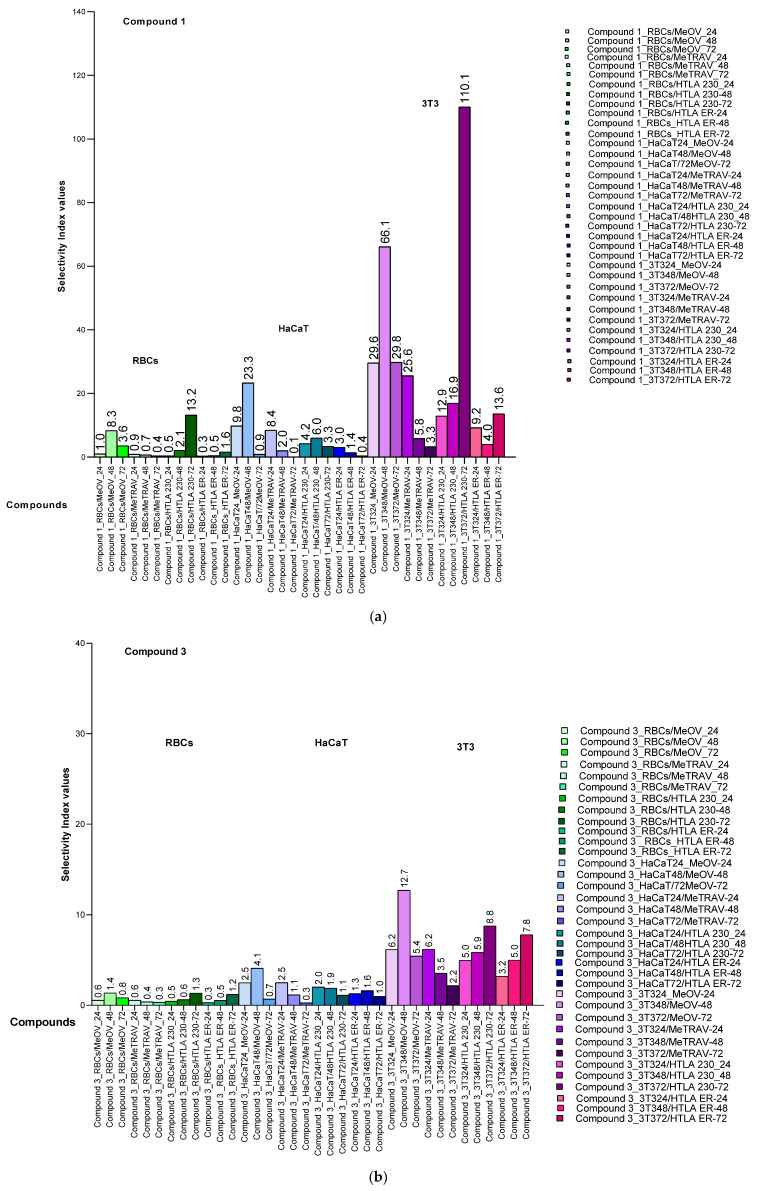

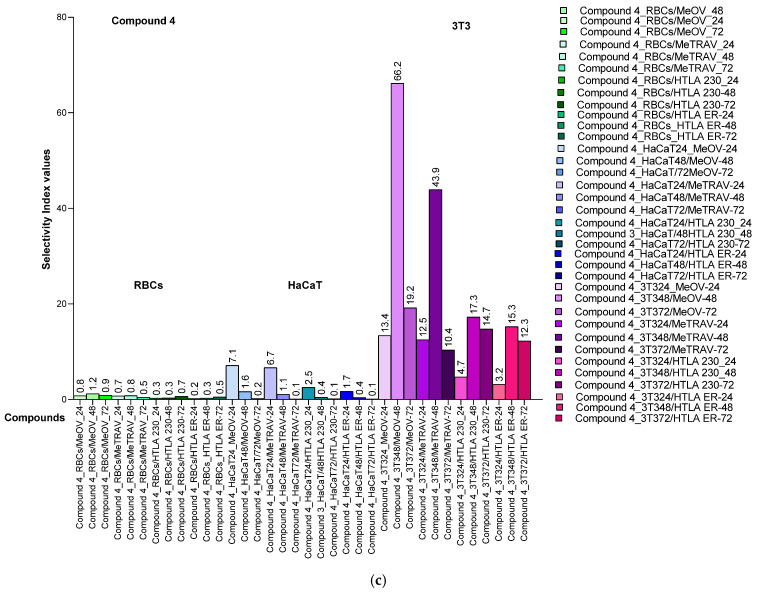
SI values of compound **1** (**a**), **3** (**b**), and **4** (**c**) for all MCM and NB cells in relation to their toxicity towards RBCs (green bars), HaCaT (blue bars), and 3T3 cells (purple–red bars).

The IC_50_ values determined after 24, 48, and 72 h of exposure of MeOV, MeTRAV, HTLA 230, HTLA ER, HaCaT, and 3T3 cells to QPSs **1**, **3**, and **4**, together with the HC_50_ values determined for RBC following exposure to QPSs for the time of experiments as per protocol [73], were used to calculate the related SIs according to Equation (1). SIs are graphically reported in Figure 17.

Concerning RBCs, the selectivity of all QPSs towards tumor cells was generally low. Specifically, compound **4** was the least selective, demonstrating SIs > 1 (1.2) only vs. MeOV in 48 h treatments. On the contrary, compound **1** was the most selective, exhibiting SIs equal to 1.0 (24 h), 8.3 (48 h), and 3.6 (72 h) vs. MeOV, 2.1 (48 h), and 13.2 (72 h) vs. HTLA 230 and 1.6 (72 h) vs. HTLA ER cells. In all remaining cases, including MeTRAV cells at all time exposure (SIs = 0.4, 0.7, and 0.9), HTLA 230 cells in 24 h treatments (SIs = 0.5), and HTLA ER cells in 24 and 48 h treatments (SIs = 0.3 and 0.5, respectively), SI values were <1, indicating limited selectivity versus cancer cells. Finally, compound **3** was sufficiently selective for MeOV (SIs = 1.4, 48 h treatment), HTLA 230 (SIs = 1.3, 72 h treatment), and HTLA ER cells (SIs = 1.2, 72 h treatment), whereas all remaining SIs values were <1. Notably, while positive SIs of compounds **3** and **4** in relation to their hemolytic toxicity versus RBCs were similar and close to 1 (1.2–1.4), those of compound **1** reached much higher values of 3.6 and 8.3 for MeOV and 13.2 for HTLA 230 cells. These findings suggest that compound **1** could be the safest candidate to minimize hemolytic toxicity in a potential future systemic clinical application. Concerning 3T3 cells, compound **1** was the least cytotoxic, with SI values from 3.3 to 110, whereas compound **3** was the most aggressive, although still maintaining acceptable selectivity index values in the range 2.2–12.8. Compound **4** exhibited an intermediate behaviour, with SIs values ranging from 3.2 to 66.2. In the cases of compounds **3** and **4**, the highest selectivity was observed against MeOV cells in 48 h treatments (SIs = 12.7 and 66.2, respectively), followed by that for HTLA 230 treated with compound **3** for 72 h treatments (SI = 8.8) and MeTRAV cells treated with compound **4** in 48 h treatments (SI = 43.9). Conversely, compound **1** displayed its highest selectivity against HTLA 230 in 72 h treatments (110.1), followed by that for MeOV in 48 h treatments (SI = 66.1). As already evinced by IC_50_ values, HaCaT tolerability to all QPSs depended on both tumor cell type and time of exposure, with long-term treatments (72 h) being the most detrimental. Specifically, compound **1** exhibited high selectivity toward all cancer cell lines in 24–48 h treatments, with SI values ranging from 1.4 to 23.3. In contrast, after 72 h exposure, SIs dropped to the range 0.1–0.9 for most cancer cell lines, except for HTLA 230, for which the SI value remained substantially higher than 1 (SI = 3.3). Despite lower SIs being obtained, a similar scenario was observed for compound **3**. This compound, which exhibited SI values > 1 (1.1–4.1) against all cancer cells in 24–48 h treatments, in 72 h treatments displayed SIs values < 1 (0.3–0.7) only for MCM cells, whereas selectivity toward NB cells remained >1 (1.0–1.1). Collectively, the compound that was less selective for cancer cells was **4**, especially towards NB cells. For HTLA 230 and HTLA ER cells, acceptable SIs > 1 were observed only in 24 h treatments (2.5 and 1.7, respectively), whereas in all other conditions, SIs were <1 (0.1–0.4). Higher selectivity was instead observed for MCM cells, for which SIs < 1 (0.1–0.2) were obtained only after long-time exposure (72 h).

Collectively, the newly developed QPSs showed, in several cases, significantly higher selectivity than the previously reported QPS BPPB [19] towards MCM cells, in relation to their cytotoxicity against HaCaT cells. Specifically, selectivity versus MeOV cells was increased 2.5- and 2.9-fold for compound **1** in 24 and 48 h treatments, respectively, and an increase of 1.8-fold was observed only in 24 h treatments for compound **4**. Moreover, the selectivity of all compounds for MeTRAV cells was higher than that of BPPB in the 24 h treatments by 7.9-, 2.4-, and 6.3-fold for compounds **1**, **3**, and **4**, respectively [19]. Although a direct comparison between the selectivity of QPSs for toward NB cells in relation to their cytotoxicity for 3T3 cells and that of BPPB was not possible for lack of data, an indirect comparison could be performed using the available SI values of BPPB obtained on MRC-5 cells [22], which are non-immortalized human lung fibroblasts derived from embryonic tissue as 3T3 cells [82]. In this regard, except for compounds **3** and **4** in 24 h treatments against HTLA 230 NB cells, all compounds were highly more selective toward both NB cell populations than BPPB [22]. Specifically, compound **1** was more selective for cancer cells than BPPB by 1.1-, 4.5-, and 27.1-fold against HTLA 230 cells after 24, 48, and 72 h exposure, respectively, and by 3.7-, 5.0-, and 12.3-fold against HTLA ER cells at the same exposure times of 24, 48, and 72 h. Compounds **3** and **4**, although less selective than BPPB toward HTLA 230 NB cells in 24 h treatments, exhibited higher selectivity in all other experimental conditions. Specifically, by 1.6–2.2-fold (compound **3**) and 3.6–4.6-fold (compound **4**) towards HTLA 230 and by 1.3–7.1-fold (compound **3**) and 1.3–19.1-fold (compound **4**) towards HTLA ER NB cells. Finally, as anticipated above, the SIs of compound **1** versus MCM cells in relation to their toxicity against 3T3 cells in 24 h exposure were higher than those of Sm^III^-EGCG by Li et al. by 1.6- (MeTRAV) and 1.8-fold (MeOV).

#### 2.6.7. Comparison of **1**, **3**, and **4** Biological Effects with Those of BPPB Using PCA

**Figure 18:**
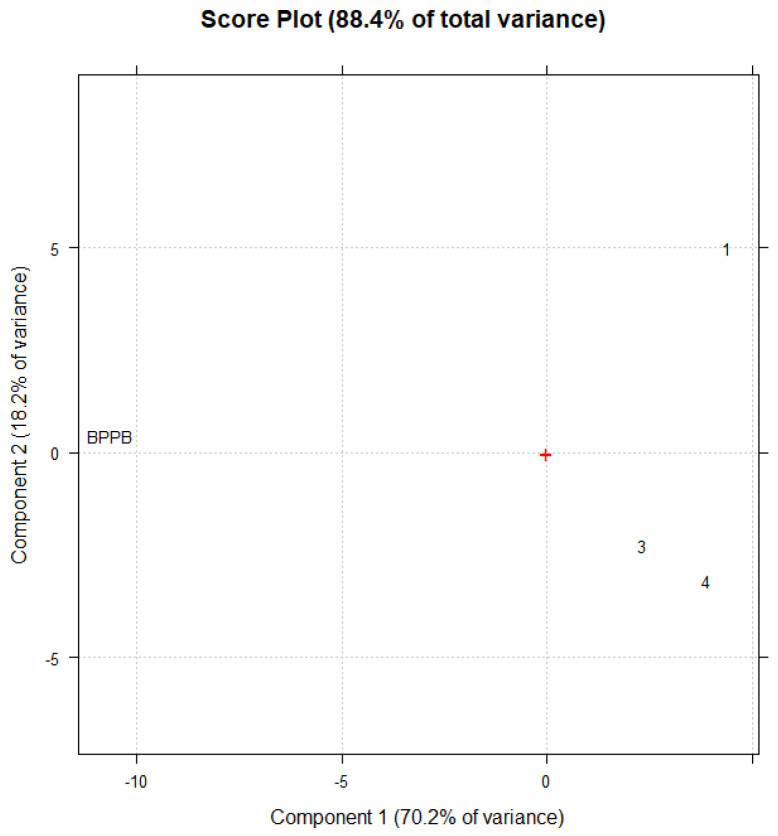
Score plot of PCA results concerning the specific dataset containing the biological findings of compounds **1**, **3**, **4**, and BPPB, represented as their scores in PC1 (70.2% of variance) versus those in PC2 (18.2% of variance). The red cross indicates the intersections at zero scores of PC1 with PC2.

The data matrix comprising 292 variables, constructed as explained in the experimental section, was processed by PCA, as performed previously. The total variance (100%) was explained by three components (PC1 explained 75.20%, PC2 explained 18.17%, and PC3 explained 11.63% of total variance). The results were reported as a score plot of PC1 versus PC2, explaining 88.4% of total variance (Figure 18).

Compounds **1**, **3**, and **4** were clustered at positive PC1 scores (close to +5), clearly separated from BPPB, which was located at negative PC1 scores (>−10), indicating a markedly different biological activity. In fact, although BPPB was significantly more active against both NB and CMM cells, in terms of IC_50_ values, it was also more cytotoxic to non-tumorigenic cells, resulting in lower SI values. In contrast, the new QPSs displayed a safer overall biological profile, supporting their potential suitability for future therapeutic applications. Separation of QPSs on PC1, locating compound **1** at positive scores (+5), well separated by compounds **3** and **4**, which are located both at negative scores (around −2.5), could indicate its high efficiency in treating cancer cells and its high SIs, as frequently observed.

## 3. Materials and Methods

### 3.1. Chemicals and Instruments

All solvents (Merck, Milan, Italy) were dried and purified according to standard procedures prior to use. When required, reactions were performed under an inert atmosphere (Ar) in pre-flamed glassware. Anhydrous Na_2_SO_4_ was used for drying solutions, and the solvents were then routinely removed at ca. 40 °C under reduced pressure (ca 10–20 mmHg), using a rotary evaporator. All reagents employed in the present work were commercially available (Merck, Milan, Italy) and used without further purification. Flash column chromatography (FCC) was performed on silica gel (70–230 and 230–400 mesh, Merck, Darmstadt, Germany). Thin-layer chromatography (TLC) was performed using aluminum-backed silica gel plates (Merck DC-Alufolien Kieselgel 60 F254, Merck, Washington, DC, USA), and detection of spots was made by UV light (254 nm) using a Handheld UV Lamp, LW/SW, 6W, UVGL-58 (Science Company^®^, Lakewood, CO, USA). Attenuated total reflectance (ATR) Fourier-transform infrared (FTIR) analyses were carried out using a Spectrum Two FT-IR Spectrometer (PerkinElmer, Inc., Waltham, MA, USA), as previously reported [83]. ^1^H, ^13^C, and ^31^P NMR spectra were acquired on a Jeol 400 MHz spectrometer (JEOL USA, Inc., Peabody, MA, USA) at 400, 100, and 162 MHz, respectively. Fully decoupled standard ^13^C NMR, ^31^P NMR spectra, and DEPT135 experiments were reported. Chemical shifts were reported in ppm (parts per million) units relative to the internal standard tetramethylsilane (TMS = 0.00 ppm) and coupling constants (*J*) were reported in Hz. In ^31^P NMR spectra, the chemical shift in free PPh_3_ in CDCl_3_ is ∼−5 ppm relative to 85% H_3_PO_4_ (0 ppm). Splitting patterns were described as follows: s (singlet), d (doublet), t (triplet), q (quartet), m (multiplet), and br (broad signal). Gas chromatography–mass spectrometry (GC-MS) spectra were performed on a Varian Saturn 2000 ion trap GC-MS instrument (Artisan Technology Group^®^, Champaign, IL, USA) equipped with a DB-5MS column (30 m, i.d. 0.25 mm) (Agilent, Santa Clara, CA, USA). Melting points are incorrect and were measured using an electric melting point device (Falc Instruments Srl, Treviglio, Bergamo, Italy), having accuracy ±1.0 to 20 °C and ±2.5 to 300 °C. Elemental analyses were carried out using an Elemental Analyzer (Fison Instruments Ltd., Farnborough, Hampshire, UK).

### 3.2. Synthesis of Phosphor-Containing Compounds ***1***–***4***

With the aim of finding novel compounds that are effectively active against difficult-to-treat late-stage cutaneous metastatic melanoma and neuroblastoma due to cell mutations and resistance developed to vemurafenib (PLX) [18] and several anticancer drugs (paclitaxel, doxorubicin, and etoposide) [16], four compounds (**1**–**4**) were synthesized and fully characterized. Three compounds (**1**, **3**, and **4**) were in the form of quaternized phosphonium salts containing the triphenyl benzyl or the diphenyl-benzyl alkyl phosphonium groups, while compound **2** was in the form of a non-quaternized uncharged phosphine derivative, having only two phenyl rings and an 11-undecanol chain directly linked to the phosphorous atom. Compound **2** was necessary to synthesize **3** and **4**.

#### 3.2.1. Benzyl Triphenyl Phosphonium Bromide (**1**) [40]

**Figure 19:**
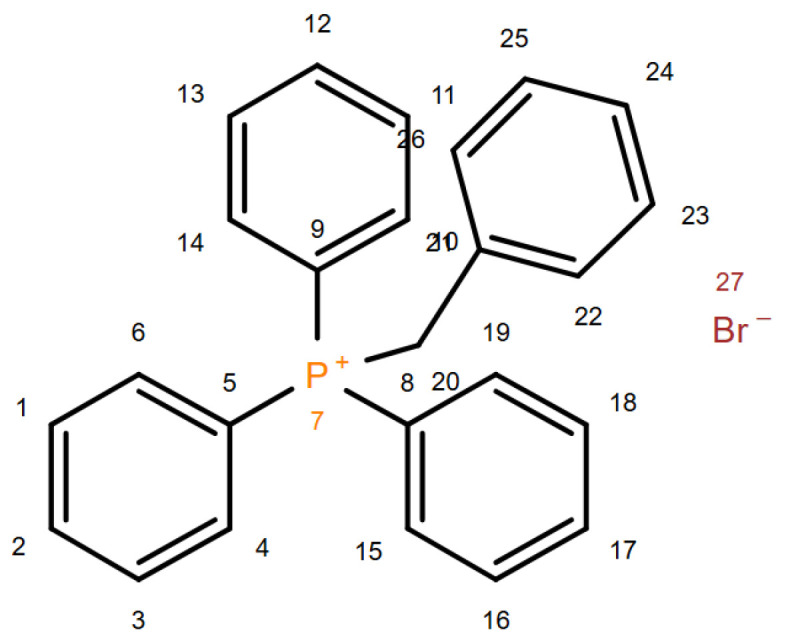
Numbered chemical structure of compound **1**.

Triphenylphosphine (1.35 g, 5.02 mmol) was added to a solution of commercial benzyl bromide (0.85 g, 4.97 mmol, and 0.6 mL) in dichloromethane (DCM) (45 mL). The mixture was heated to reflux for 24 h, left to reach room temperature, and then maintained under stirring for an additional 24 h, during which a precipitate formed. Diethyl ether (Et_2_O) was added to complete precipitation, and the solid was filtered to give the product a 97.3% yield (2.09 g, 4.84 mmol), as white crystals with a high level of purity and no need for further purification. The numbered structure of compound **1** is showed in Figure 19.

M.p.: 293–294 °C; 298 °C (lit) [41], 298–301 °C (lit) [42]. ATR-FTIR (ν, cm^−1^): 3085, 3052, 3010 (=C-H stretching), 2859, 2773 (C-H stretching), 1456, 1436 (C-H banding), 719, 689 (C-P stretching). ^1^H NMR (400 MHz, CDCl_3_, ambient °C): δ (ppm) = 7.71 (s, *J* = 7.7, 7.4, 3.7 Hz, 6 equivalent H), 7.65 (s, *J* = 13.3, 7.7 Hz, 6 equivalent H), 7.45 (t, *J* = 7.4 Hz, 3 equivalent H), 7.26 (s, *J* = 7.6, 7.4 Hz, 2 equivalent H), 7.22 (s, *J* = 7.6, 3.3 Hz, 2 equivalent H), 7.22 (t, *J* = 7.4 Hz, 1H), 5.97 ppm (d, *J* = 15.2 Hz, 2H CH_2_). ^13^C NMR (101 MHz, CDCl_3,_ ambient °C): δ (ppm) = 135.1 (s, 3 equivalent C), 134.3, (d, *J*^2^_C-P_ = 10.20 Hz, 6 equivalent C), 132.2 (d, *J*^3^_C-P_ = 5.26 Hz, 2 equivalent C), 130.2 (d, *J*^3^_C-P_ = 5.26 Hz, 2 equivalent C), 130.2 (d, conjugated *J*^3^_C-P_ = 12.70 Hz, 6 equivalent C), 130.0 (d, *J*^4^_C-P_ = 3.20 Hz, 2 equivalent C), 129.6 (s, 1C), 128.5 (d, not conjugated *J*^2^_C-P_ = 8.68 Hz, 1C), 117.1 (d, aromatic *J*^1^_C-P_ = 86.20 Hz, 3 equivalent C), 28.1 (d, aliphatic *J*^1^_C-P_ = 48.10 Hz, CH_2_). ^31^P NMR (162 MHz, CHCl_3_, ambient °C): δ (ppm) = −5 (PPh_3_), +21.76 (s, 1P). Calcd. for C_25_H_22_BrP, Mol. Wt. (MW): 433.32. Found exact mass: 432.06; (GC/MS) (*m*/*z*): m/e: 432.06 (100.0%), 434.06 (97.3%), 433.07 (28.1%), 435.07 (27.7%), 434.07 (3.8%), 436.07 (3.7%). Anal. Calcd. C, 69.29%; H, 5.12%; Br, 18.44%; P, 7.15%. Found: C, 69.57%; H, 4.98%; Br, 18.61%; P, 6.99%.

#### 3.2.2. 11-(Di-phenyl-phosphine)-1-undecanol **2** [43]

**Figure 20:**
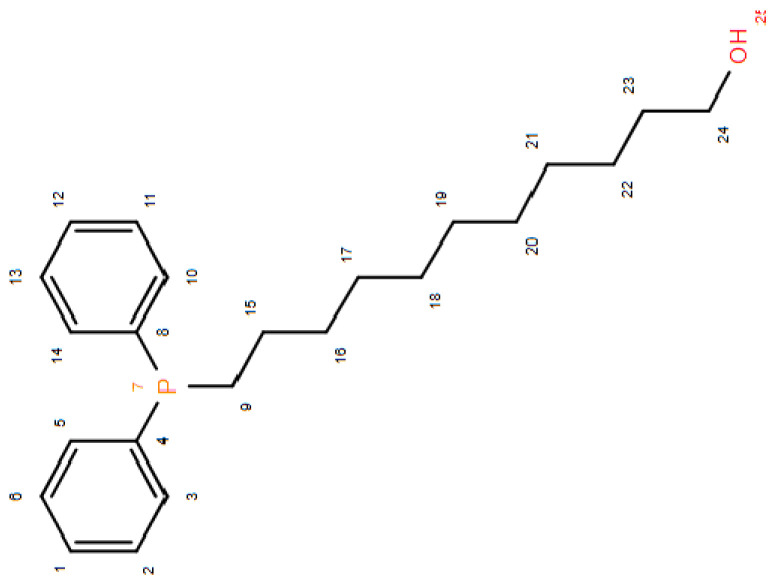
Numbered chemical structure of compound **2**.

The numerated structure of **2** is shown in Figure 20.

In a two-necked flask flamed under a stream of nitrogen, a 2/1 excess of sodium hydride (60% in mineral oil) (1.17 g, 48.68 mmol) was suspended in dimethyl sulfoxide (DMSO, 31 mL) and heated to 70 °C to promote dissolution. Then, triphenyl-phosphonium undecane-11-olo was added [28] (7.36 g, 14.33 mmol), observing an immediate orange coloration. The mixture was then stirred at 50 °C for 1 h until the reagent disappeared (TLC DCM/MeOH 9/1). The reaction was then quenched with NH_4_Cl^−^ and the aqueous phase was extracted with Et_2_O (3 × 30 mL). The organic phases were unified and washed with H_2_O and dried over Na_2_SO_4_ overnight. After removal of the solvent under reduced pressure, a dark oil was obtained (6.32 g), which was subjected to flash column chromatography (FCC) to obtain pure **2**, in the form of orange oil (4.14 g, 11.61 mmol, 81.0% yield). Rf. 0.48 (Et_2_O/hexane 1/1).

Oil. ATR-FTIR (ν, cm^−1^): 3366 (O-H stretching), 3056 (=C-H stretching), 2924, 2852 (C-H stretching), 1484–1437 (C-H banding), 1173, 1120 (C-O stretching), 746, 721, 691 (C-P stretching). ^1^H NMR (400 MHz, CDCl_3_, ambient °C): δ (ppm) = 7.46 (t, *J* = 7.5, 7.0 Hz, 4 equivalent H), 7.05 (s, *J* = 7.5 Hz, 4 equivalent H), 6.97 (s, *J* = 7.5 Hz, 2 equivalent H), 3.57 (td, *J* = 6.4, 5.0 Hz, 2H, CH_2_), 2.29 (dt, *J* = 8.0, 6.6 Hz, 2H, CH_2_), 1.54 (dtt, *J* = 8.8, 7.2, 6.6 Hz, 2H, CH_2_), 1.53 (quin, *J* = 6.9, 6.4 Hz, 2H, CH_2_), 1.37 (s, *J* = 7.7, 6.8 Hz, 2H, CH_2_), 1.33 (s, *J* = 6.9, 6.8 Hz, 2H, CH_2_), 1.29 (s, *J* = 7.5, 7.2, 4.8 Hz, 2H, CH_2_), 1.28 (s, *J* = 7.7 Hz, 2H, CH_2_), 1.28 (s, *J* = 7.7 Hz, 2H, CH_2_), 1.28 (s, *J* = 7.7, 7.5, 6.0 Hz, 2 equivalent H, CH_2_), 1.27 ppm (s, *J* = 7.7 Hz, 2H, CH_2_). ^13^C NMR (101 MHz, CDCl_3,_ ambient °C): δ (ppm) = 132.7 (d, *J*^2^_C-P_ = 17.76 Hz, 4 equivalent C), 128.6 (d, *J*^3^_C-P_ = 6.00 Hz, 4 equivalent C), 128.4 (d, *J*^4^_C-P_ = 7.00 Hz, 2 equivalent C), 62.9 (s, CH_2_OH), 33.0 (s, CH_2_), 29.8 aliphatic *J*^1^_C-P_ = 12.24 Hz CH_2_), 29.7, 29.7, 29.6 (3s, CH_2_), 29.5 (d, aliphatic *J*^5^_C-P_ = 4.97 Hz, CH_2_), 29.3 (d, aliphatic *J*^4^_C-P_ = 5.00 Hz, CH_2_), 29.0 (d, aliphatic *J*^3^_C-P_ = 10.55 Hz, CH_2_), 26.3 (s, CH2), 25.8 (d, aliphatic *J*^2^_C-P_ = 14.84 Hz, CH_2_). ^31^P NMR (162 MHz, CHCl_3_, ambient °C): δ (ppm) = −5 (PPh_3_), −15.40 (s, 1P). Calcd. for C_23_H_33_OP, Mol. Wt.: 356.48. Found exact mass: 356.23. GC/MS (m/e): 356.23 (100.0%), 357.23 (26.1%), 358.23 (3.3%). Calcd.: C, 77.49%; H, 9.33%; O, 4.49%; P, 8.69%. Found: C, 77.16%; H, 9.61%; O, 4.47%; P, 9.00%.

#### 3.2.3. Benzyl-(11-hydroxyundecyl)-diphenyl-phosphonium Bromide (**3**) and Benzyl-diphenyl-undec-10-enyl-phosphonium bromide (**4**)

Commercial benzyl bromide (0.8115 g, 4.74 mmol, and 0.6 mL), oily 11-(di-phenyl-phosphine)-1-undecanol (11-DPPOH, 1.99 g, 5.56 mmol), and DCM (7 mL) were stirred in a 50 mL two-necked round-bottomed flask equipped with a reflux condenser for 24 h. Then, the mixture was left to reach room temperature and was maintained under stirring for an additional 24 h. The solvent was removed under reduced pressure, and the oily residue was treated with diethyl ether (Et_2_O) (80 mL) and stirred overnight. Upon decantation, two phases formed. The supernatant was separated and evaporated in a vacuum, obtaining **4** as a brown oil, which had a negative Schiff test (OH absent) (0.9424 g, 1.84 mmol, 39.02%, and Rf. 0.2581 (MeOH/Petrol 1/1)). The residue was dissolved in acetone, transferred, and after removal of solvent at reduced pressure, **3** was obtained as dark-brown oil, which had a positive Schiff test (OH) (1.01 g, 1.91 mmol, 40.3%, and Rf. 0.1828 (MeOH/Petrol 1/1)).

**Figure 21:**
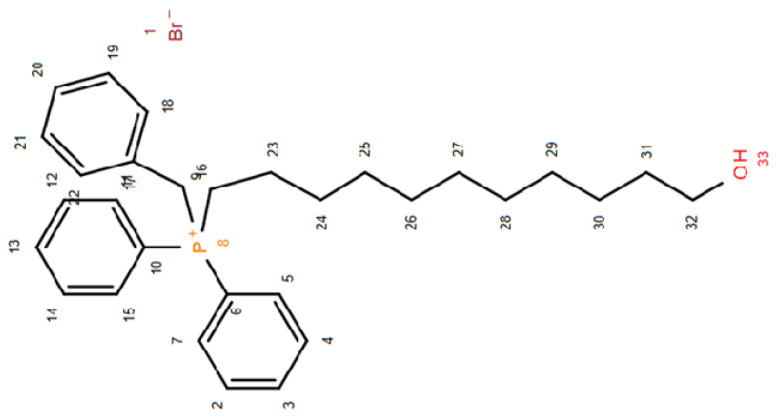
Numbered chemical structure of compound **3**.

The numerated structure of **3** is shown in Figure 21.

Compound **3**. Brown oil. ATR-FTIR (ν, cm^−1^): 3334 (O-H stretching), 3056, 3026 (=C-H stretching), 2924, 2852 (C-H stretching), 1455–1437 (C-H banding), 1160, 1120 (C-O stretching), 743, 720, 694 (C-P stretching). ^1^H NMR (400 MHz, CDCl_3_, ambient °C): δ (ppm) = 8.37 (dd, *J* = 7.7, 5.6 Hz, 4 equivalent H), 7.60 (s, *J* = 7.4 Hz, 2 equivalent H), 7.52 (s, *J* = 7.7, 7.4, 5.0 Hz, 4 equivalent H), 7.45 (dd, *J* = 7.6, 3.3 Hz, 2 equivalent H), 7.28 (t, *J* = 7.4 Hz, 1H), 7.17 (t, *J* = 7.6, 7.4 Hz, 2 equivalent H), 4.27 (d, *J* = 13.9 Hz, 2H, CH_2_), 3.65 (td, *J* = 6.4, 5.0 Hz, 2H, CH_2_), 3.12 (dt, *J* = 11.9, 6.8 Hz, 2H, CH_2_), 1.53 (quin, *J* = 6.9, 6.4 Hz, 2H, CH_2_), 1.47 (s, *J* = 7.9, 7.2, 6.8 Hz, 2H, CH_2_), 1.45 (s, *J* = 7.5, 7.2 Hz, 2H, CH_2_), 1.32 (quin, *J* = 6.9, 6.8 Hz, 2H, CH_2_), 1.23 (s, *J* = 7.7, 6.80 Hz, CH_2_), 1.23 (s, *J* = 7.7, 7.5, 6.0 Hz, 2 equivalent H, CH_2_), 1.21 (s, *J* = 7.7 Hz, 2H, CH_2_), 1.19 (s, *J* = 7.7 Hz, 2H, CH_2_), 1.16 ppm (s, *J* = 7.7 Hz, 2H, CH_2_). ^13^C NMR (101 MHz, CDCl_3_, ambient °C): δ (ppm) = 134.5 (s, 2 equivalent C), 132.5 (d, *J*^2^_C-P_ = 10.38 Hz, 4 equivalent C), 130.96 (d, not conjugated *J*^3^_C-P_ = 5.30 Hz, 2 equivalent C), 130.4 (d, conjugated *J*^3^_C-P_ = 12.60 Hz, 4 equivalent C), 128.9 (d, not conjugated *J*^4^_C-P_ = 3.20 Hz, 2 equivalent C), 128.5 (s, 1C), 128.1 (d, not conjugated *J*^2^_C-P_ = 8.90 Hz 1C), 117.4 (d, aromatic *J*^1^_C-P_ = 83.73 Hz, 2 equivalent C), 62.3 (s, CH_2_OH), 32.4 (s, CH_2_), 30.3 (d, aliphatic *J*^3^_C-P_ = 13.85 Hz CH_2_), 29.8 (d, aliphatic *J*^4^_C-P_ = 5.00 Hz CH_2_), 29.6, 29.6 (2s, 2CH_2_), 29.5 (d, aliphatic *J*^5^_C-P_ = 4.97 Hz CH_2_), 29.6 (d, aliphatic *J*^1^_C-P_ = 47.30 Hz CH_2_), 28.6, 25.8 (2s, CH_2_), 23.2 (d, aliphatic *J*^1^_C-P_ = 45.20 Hz CH_2_), 21.9 (d, aliphatic *J*^2^_C-P_ = 4.05 Hz CH_2_). ^31^P NMR (162 MHz, CHCl_3_, ambient °C): δ (ppm) = −5 (PPh_3_), +23.82 (s, 1P). Calcd. for C_30_H_40_BrOP, Mol. Wt. (MW): 527.52. Found exact mass: 526.20; (GC/MS) (m/e): 526.20 (100.0%), 528.20 (97.5%), 527.20 (33.4%), 529.20 (33.1%), 528.21 (5.6%), 530.20 (5.4%). Anal. Calcd. C, 68.31; H, 7.64; Br, 15.15; O, 3.03; P, 5.87. Found: C, 68.05%; H, 7.88%; Br, 14.93%; P, 5.58%.

**Figure 22:**
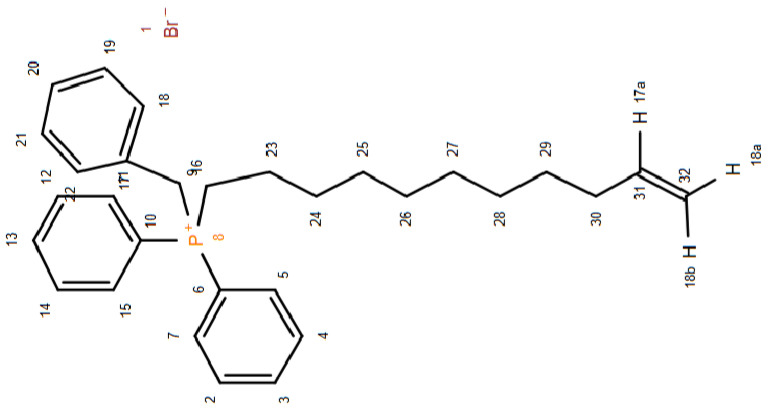
Numbered chemical structure of compound **4**.

The numbered structure of **4** is shown in Figure 22.

Compound **4**. Brown oil. ATR-FTIR (ν, cm^−1^): 3056, 3029 (=C-H stretching), 2925, 2853 (C-H stretching), 1454–1437 (C-H banding), 743, 720, 694 (C-P stretching). ^1^H NMR (400 MHz, CDCl_3_, ambient °C): δ (ppm) = 8.37 (dd, *J* = 7.7, 5.6 Hz, 4 equivalent H), 7.60 (s, *J* = 7.4 Hz, 2 equivalent H), 7.52 (s, *J* = 7.7, 7.4, 5.0 Hz, 1H), 7.45 (dd, *J* = 7.6, 3.3 Hz, 4 equivalent H), 7.45 (dd, *J* = 7.6, 3.3 Hz, 1H), 7.28 (t, *J* = 7.4 Hz, 1H), 7.17 (t, *J* = 7.6, 7.4 Hz, 2 equivalent H), 5.76 (ddt, *J* = 15.3, 12.1, 6.4, 6.3 Hz, 1H, CH=CH_2_), 4.99 (d, *J* = 15.3 Hz, 1Hb, CH=CH_2_), 4.94 (d, *J* = 12.1 Hz, 1Ha, CH=CH_2_), 4.27 (d, *J* = 13.9 Hz, 2H, CH_2_), 3.12 (dt, *J* = 11.9, 6.8 Hz, 2H, CH_2_), 2.07 (s, *J* = 16.7, 6.9, 6.3 Hz, 2 equivalent H, CH_2_), 1.47 (s, *J* = 7.9, 7.2, 6.8 Hz, 2H, CH_2_), 1.45 (s, *J* = 7.5, 7.2 Hz, 2H, CH_2_), 1.37 (quin, *J* = 6.9 Hz, 2H, CH_2_), 1.25 (s, *J* = 7.7, 7.5, 6.0 Hz, 2 equivalent H, CH_2_), 1.24 (s, *J* = 7.5, 6.9, 6.0 Hz, 2 equivalent H, CH_2_), 1.20 (s, *J* = 7.7 Hz, 2H, CH_2_), 1.19 ppm (s, *J* = 7.7, 7.5 Hz, 2H, CH_2_). ^13^C NMR (101 MHz, CDCl_3,_ ambient °C): δ (ppm) = 138.4 (s, 1C, CH=CH_2_), 134.5 (s, 2 equivalent C), 132.5 (d, *J*^2^_C-P_ = 10.38 Hz, 4 equivalent C), 130.96 (d, 3*J*^2^_C-P_ = 5.30 Hz, 2 equivalent C), 130.4 (d, conjugated *J*^3^_C-P_ = 12.60 Hz, 4 equivalent C), 128.9 (d, not conjugated *J*^4^_C-P_ = 3.20 Hz, 2 equivalent C), 128.5 (s, 1C), 128.1 (d, not conjugated *J*^2^_C-P_ = 8.90 Hz 1C), 117.4 (d, *J*^1^_C-P_ = 83.73 Hz, 2 equivalent C), 114.4 (s, 1C, CH=CH_2_), 33.6 (s, CH_2_), 30.3 (d, aliphatic *J*^3^_C-P_ = 13.85 Hz CH_2_), 29.7 (d, aliphatic *J*^4^_C-P_ = 5.00 Hz CH_2_), 29.6 (d, aliphatic *J*^1^_C-P_ = 47.30 Hz CH_2_), 29.3 (d, aliphatic *J*^5^_C-P_ = 4.97 Hz CH_2_), 29.03, 28.97, 28.31 (3s, CH_2_), 23.2 (d, aliphatic *J*^1^_C-P_ = 45.20 Hz CH_2_), 21.9 (d, aliphatic *J*^2^_C-P_ = 4.05 Hz, CH_2_). ^31^P NMR (162 MHz, CHCl_3_, ambient °C): δ (ppm) = −5 (PPh_3_), +23.82 (s, 1P). Calcd. for C_30_H_38_BrP, Mol. Wt. (MW): 509.50. Found exact mass: 508.19; (GC/MS) (m/e): 508.19 (100.0%), 510.19 (97.3%), 509.19 (33.4%), 511.19 (33.0%), 510.20 (5.6%), 512.19 (5.2%). Anal. Calcd. C, C, 70.72; H, 7.52; Br, 15.68; P, 6.08. Found: C, 70.91%; H, 7.94%; Br, 15.99%; P, 5.95%.

### 3.3. ATR-FTIR Spectroscopy of Compounds ***1***–***4***

ATR-FTIR spectra of compounds **1**–**4** were recorded in triplicate directly on the solid samples in attenuated total reflection (ATR) mode. Acquisitions were made from 4000 to 600 cm^−1^, with 1 cm^−1^ spectral resolution, co-adding 32 interferograms, with a measurement accuracy in the frequency data at each measured point of 0.01 cm^−1^, due to the laser internal reference of the instrument. The frequency of each band was obtained automatically by using the “find peaks” command in the instrument’s software.

### 3.4. UV-Vis Analyses

Compounds **1**–**4** were dissolved in MeOH, and solutions were properly diluted. The UV-Vis spectrum of all samples was acquired at room temperature, using an Agilent Cary 100 UV/Vis Spectrophotometer (Agilent Technologies Italia S.p.A., Milan, Italy). Analyses were performed in triplicate, and the images reported in the Results and Discussion section are representative of the acquisitions obtained for each sample.

### 3.5. Multivariate Analysis of ATR-FTIR, UV-Vis, and NMR Spectral Data

#### 3.5.1. ATR-FTIR and UV-Vis Spectral Data

ATR-FTIR data (transmittance, %) of all acquired spectra were arranged in a matrix of 3401 (wavenumbers cm^−1^) × 4 (compounds) = 13,604 measurable variables. Then, spectral data of previously reported triphenyl phosphonium salt (BPPB) were added to this matrix [29], obtaining a second dataset of 3401 (wavenumbers cm^−1^) × 5 (compounds) = 17,005 measurable variables. For each sample, the variables consisted of the values of transmittance (%) associated with the wavenumbers (3401) in the range 4000–600 cm^−1^. UV-Vis data (absorbance, %) of all acquired spectra were arranged in a matrix of 91 (wavelength nm) × 4 (compounds) = 364 measurable variables. Then, UV-Vis data of previously reported triphenyl phosphonium salt (BPPB) were added to this matrix [29], obtaining a second dataset of 91 (wavelength nm) × 5 (compounds) = 455 measurable variables. For each sample, the variables consisted of the values of absorbance associated with the wavelength (91) in the range 340–250 nm. Finally, a matrix 3492 × 5 (17,460 measurable variables) containing both the ATR-FTIR data and the UV-Vis data of compounds **1**–**4** and BPPB was constructed. The systems were simplified by exploiting the multivariate analysis, named principal component analysis (PCA), processing each matrix of spectral data using CAT (Chemometric Agile Tool, which is freely available online at https://www.gruppochemiometria.it/index.php/software/19-download-the-r-based-chemometric-software, accessed on 23 November 2025). Before PCA, ATR-FTIR spectral data were scaled and centered, while UV-Vis data were first standardized by the standard normal variate (SNV) method and then also scaled and centered. The results were reported as score plots of PC1 vs. PC2 and discussed in Section 3.

#### 3.5.2. NMR Spectral Data

The values of δ (ppm) of all peaks present in the ^1^H, ^13^C, and ^31^P NMR spectra of the sample, which showed the highest number of peaks, represented the first row of a matrix of four total rows, one for compounds **1**–**4**. Then, in rows 2–4, the values of δ (ppm) of peaks present in the spectra of remaining compounds were inserted, taking the first row as reference. Where certain peaks were missing, zero was inserted. A matrix of 41 (δ, ppm) × 4 (compounds) = 164 measurable variables was obtained. Then, the values of δ (ppm) of all peaks present in the ^1^H, ^13^C, and ^31^P NMR spectra of previously reported triphenyl phosphonium salts, namely BPPB [29] and TPPOH [28], were added to the first dataset, using the same method, obtaining a second larger matrix of 41 (δ, ppm) × 6 (compounds) = 246 measurable variables. For each sample, the variables consisted of the values of δ (ppm) of all peaks present in their NMR spectra. The systems were simplified by exploiting PCA, as detailed in the previous section. Before PCA, ^13^C NMR spectral data were scaled and centered. The results were reported as a score plot of PC1 vs. PC2 and PC1 vs. PC3 and discussed in Section 3.

#### 3.5.3. Biological Findings

The most significant biological data obtained for compounds 1, 3, and 4 in terms of HC_50_, IC_50_, and SI values in this study, together with those previously reported for BPPB were arranged in a data matrix consisting of four rows (compounds) and 73 columns (variables), for a total of 292 variables, and processed by PCA, as done previously. The total variance (100%) was explained by three components (PC1 explained 75.20%, PC2 explained 18.17%, and PC3 explained 11.63% of total variance). Before PCA, the data were scaled and centered. The results were reported as a score plot of PC1 vs. PC2 and discussed in Section 3.

### 3.6. Optical Microscopy Analyses

Methanol (MeOH) and water solutions/suspensions of compounds **1**–**4** were investigated via optical microscopy (OM) analysis to assess their possible capability to give vesicles in solvents. In the performed experiments, 30.1 mg of solid **1** and 53.2, 37.1, and 47.0 mg of oily compounds **2**, **3**, and **4**, respectively, were dissolved in 0.5 mL MeOH under vigorous shaking until complete dissolution. The obtained solutions of **1**, **2**, **3**, and **4** had concentrations of 60.2, 106.4, 74.2, and 94 mg/mL (138.9, 298.5, 140.7, and 184.5 mM), respectively. Then, 7.5 mg of solid **1** and 12.7, 12.3, and 18.8 mg of oily compounds **2**, **3**, and **4**, respectively, were dissolved in 0.65 mL or 1.0 mL of water and under sonication, until a partial suspension was obtained. Final concentrations were 11.5, 12.7. 12.3, and 18.8 mg/mL (26.5, 35.6, 23.3, and 36.9 mM). The prepared samples were then observed using a Leica DM750 optical microscope (Leica Italy, Milan, Italy) equipped with 40× and 100× objectives. The camera used for image capture was a Leica ICC50W (Leica Italy, Milan, Italy). All images were processed using LAS EZ 3.4.0. software (Leica Italy, Milan, Italy).

### 3.7. Cytotoxicity Experiments Using Compounds ***1***, ***3***, and ***4*** on PLX-Resistant MCM (MeOV and MeTRAV) Cells, as Well as on ETO-Sensitive (HTLA 230) and MDR (HTLA ER) NB Cells

#### 3.7.1. Cell Lines and Culture Conditions

PLX4032-resistant (PLX-R) MeOV and MeTRAV cells were selected and characterized as previously reported [18,21]. HTLA-230 human stage-IV NB cells were kindly provided by the G. Gaslini Institute (Genoa, Italy). HTLA-ER cells were generated as previously reported [22]. All cell lines were maintained in RPMI 1640 medium (Euroclone Spa, Pavia, Italy) supplemented with 10% Fetal Bovine Serum (FBS, Euroclone Spa, Pavia, Italy), 1% L-Glutamine (Euroclone Spa, Pavia, Italy), and 1% Penicillin/Streptomicin (Euroclone Spa, Pavia, Italy) and grown in standard conditions (37 °C humidified incubator with 5% CO_2_).

#### 3.7.2. Treatments

PLX-R MeOV and MeTRAV cell lines, as well as HTLA 230 and HTLA ER ones, were treated for 24, 48, and 72 h with increasing concentration (1–100.0 µM) of compounds **1**, **3**, and **4**. Such a range of concentrations (three orders of magnitude lower than those used to acquire optical images) ensured that all samples were administered to cells in non-aggregated forms. The stock solutions of these compounds were prepared in 40,000-fold diluted DMSO, and preliminary experiments demonstrated that the final DMSO concentrations did not change any of the cell responses analyzed. Cell cultures were carefully monitored before and during the experiments to ensure optimal cell density. Notably, samples were discarded if the cell confluence reached >90%.

#### 3.7.3. Cell Viability Assay

Cell viability was determined by using the CellTiter 96^®^ AQueous One Solution Cell Proliferation Assay (Promega, Madison, WI, USA), as previously described [84,85]. Briefly, cells (10,000 cells/well) were seeded into 96-well plates (Corning Incorporated, Corning, NY, USA) and then treated. Next, cells were incubated with CellTiter, and the absorbance at 490 nm was recorded using a microplate reader (EL-808, BIO-TEK Instruments Inc., Winooski, VT, USA). The cell survival rate, expressed as cell viability percentage (%), was evaluated based on the experimental outputs of treated groups vs. the untreated groups (CTR) and was calculated as follows: cell viability (%) = (OD treated cells − OD blank)/(OD untreated cells − OD blank) × 100%. Related bar graphs of cell viability vs. increasing concentrations of **1**, **3**, and **4** were provided by GraphPad Prism 8.0.1 Software (GraphPad Software, Boston, MA, USA). The same software was used to calculate IC_50_ values. Particularly, the bar graphs were converted into the corresponding dispersion graphs. Then, upon conversion of µM concentrations (x) in Log_10_ (x), and using a nonlinear model that considered the Log _10_ (**1**, **3**, and **4** concentrations) vs. the normalized response (variable or not variable slope), the IC_50_ values of all compounds for both cell populations at 24, 48, and 72 h of treatment were derived.

### 3.8. In Vitro Hemolytic Toxicity of Samples ***1***, ***3***, and ***4*** Using Red Blood Cells (RBCs)

Hemolytic ratio was evaluated, as recently reported with slight changes [73], in EDTA-blood samples from six healthy donors from the San Martino Hospital Transfusion Centre. In detail, red blood cells (RBCs) were isolated by diluting 0.2 mL of blood with 0.4 mL of phosphate-buffered saline (PBS) and centrifuging for 5 min at 10,000 g. The pellet, consisting of RBCs, was washed 5 times with 1.0 mL PBS and finally resuspended in 2.0 mL PBS. The assay was carried out on 0.1 mL of resuspended RBCs added to 0.1 mL of H_2_O (positive control), 0.1 mL of PBS (negative control), or 0.1 mL of the different concentrations (0.1–100 µM) of the compounds to be tested. Such a range of concentrations (three orders of magnitude lower than those used to acquire optical images) ensured that all samples were administered to cells in non-aggregated forms. The samples were incubated for 60 min at 37 °C, and at the end, they were centrifuged for 5 min at 10,000 rpm. Finally, 0.1 mL of supernatant was transferred to a transparent 96-well plate and measured by a spectrophotometer (Dynex Technologies; Chantilly, VA, USA) at 595 nm. The hemolytic ratio was calculated using the following formula:

Haemolytic ratio (%) = (OD_TEST_ − OD_NEGATIVE CONTROL_)/(OD_POSITIVE CONTROL_ − OD_NEGATIVE CONTROL_) × 100

Related bar and dispersion graphs of hemolytic ratio percentage (%) and of cell viability vs. increasing concentrations of **1**, **3**, and **4** were provided by GraphPad Prism 8.0.1 Software (GraphPad Software, Boston, MA, USA). The same software was used to calculate HC_50_ values of **1**, **3**, and **4**, as previously described.

### 3.9. Evaluation of Cytotoxicity of Samples ***1***, ***3***, and ***4*** on Human Keratinocytes (HaCaT) and Murine Fibroblasts (3T3)

#### 3.9.1. Cell Culture

Human skin keratinocyte cells (HaCaT) were obtained from the IRCCS Ospedale Policlinico San Martino, Proteomics and Mass Spectrometry Unit (Genoa, Italy), while 3T3 cells were purchased from Sigma Aldrich (Milan, Italy). Both cell lines were maintained in DMEM medium (Euroclone Spa, Pavia, Italy) supplemented with 10% Fetal Bovine Serum (FBS, Euroclone Spa, Pavia, Italy), 1% L-Glutamine (Euroclone Spa, Pavia, Italy), and 1% Penicillin/Streptomicin (Euroclone Spa, Pavia, Italy) and grown in standard conditions (37 °C humidified incubator with 5% CO_2_).

#### 3.9.2. Treatments

HaCaT and 3T3 cells were treated for 24, 48, and 72 h with increasing concentrations (1–100.0 µM) of **1**, **3**, and **4**. Such a range of concentrations (three orders of magnitude lower than those used to acquire optical images) ensured that all samples were administered to cells in non-aggregated forms. Cell cultures were carefully monitored before and during the experiments to ensure optimal cell density. Notably, samples were discarded if the cell confluence reached >90%.

#### 3.9.3. Viability Assay

Viability assay on HaCaT and 3T3 cells was performed as described in Section 3.7.3 for tumour cells.

Related bar and dispersion graphs of cell (HaCaT and 3T3) viability (%) vs. increasing concentrations of **1**, **3**, and **4** were provided by GraphPad Prism 8.0.1 Software (GraphPad Software, Boston, MA, USA). The same software was used to calculate all IC_50_ values of **1**, **3**, and **4**, as previously described.

### 3.10. Comparison Between the Biological Effects of ***1***, ***3***, and ***4*** and Those of BPPB by PCA

A matrix 73 × 4 (292 measurable variables) containing all biological data acquired for compounds **1**, **3**, **4**, and BPPB was constructed. The systems were simplified by exploiting multivariate analysis, named principal component analysis (PCA), processing it using CAT (Chemometric Agile Tool, which is freely available online at https://www.gruppochemiometria.it/index.php/software/19-download-the-r-based-chemometric-software, accessed on 23 November 2025). Before PCA, the data were scaled and centered. The results were reported as score plots of PC1 vs. PC2 and discussed in Section 2.

### 3.11. Statistical Analyses

Results have been expressed as means ± S.D. of four independent experiments in which different wells were analyzed every time for each experimental condition. In the analysis of cell viability, the condition of untreated cells was set as 100% ± S.D. Statistical significance of differences was determined by one-way analysis of variance (ANOVA) followed by Dunnett’s multiple comparison test correction using GraphPad Prism 8.0.1 (GraphPad Software, San Diego, CA, USA). Asterisks indicate the following *p*-value ranges: *: *p* < 0.05, **: *p* < 0.01, ***: *p* < 0.001, and ****: *p* < 0.0001. *p* > 0.05 was not considered statistically significant, and no symbol was used in the images.

## 4. Conclusions

Systematic studies on genetic alterations in human malignancies have supported the development of genotype-driven targeted drugs for several types of cancers, including metastatic cutaneous melanoma (MCM). Nonetheless, the occurrence of acquired resistance remains a major limitation in the treatment of MCM and other aggressive tumors, including late-stage high-risk neuroblastoma (HR-NB). In this context, the identification of novel alternative therapeutic strategies able to overcome chemoresistance represents a pressing need. Quaternized phosphonium salt (QPSs), including triphenyl phosphonium (TPP) QPS derivatives, known for their membrane-disrupting properties and their ability to selectively accumulate in mitochondria, constitute a promising alternative approach. In this study, three novel QPS-type cationic vesicles were designed, synthesized, and biologically characterized. All compounds showed significant cytotoxicity against PLX-resistant MCM cells, as well as ETO-sensitive and MDR HR-NB cells, with effective responses already after short-time exposure, depending on cancer cell type. Compared with previously reported structurally similar systems, including BPPB, these new QPSs showed reduced hemolytic activity and lower/no toxicity toward non-tumorigenic cells, while maintaining a marked anticancer effect. Selectivity index values and PCA further confirmed that these new QPSs display a biological profile clearly distinct from that of BPPB, characterized by a more favourable safety–efficacy balance, particularly for compounds **1** and **4**. From a translational perspective, compound **1** emerges as a promising platform for intermediate-time treatment for PLX-R MeOV cells, while **4** appears particularly suitable for short-to-intermediate-term (24–48 h) treatments for PLX-R MeTRAV cells. Moreover, all three compounds demonstrated relevant activity against both ETO-sensitive and MDR late-stage HR-NB cells, outperforming ETO by 1.2, 2.0, and 1.3 times (HTLA 230) and 3.2, 4.7, and 3.2 times (HTLA ER) in 24 h treatments. Notably, most of our QPSs also outperformed PLX in the 72 h treatment of PLX-resistant MCM cells, depending on MCM subtypes. Beyond their potential as standalone agents, these compounds may represent valuable candidates for incorporation into combination regimens aimed at overcoming resistance in PLX-resistant MCM and MDR HR-NB. Within this translational framework, the biological data provide additional mechanistic insight. The biphasic response observed in PLX-R MeTRAV cells, together with the differential sensitivity detected in non-tumoral HaCaT and NIH/3T3 fibroblasts, indicates that the activity of these QPSs is strongly context-dependent. The marked proliferative stimulation at sub-cytotoxic concentrations, in PLX-R MeTRAV cells under prolonged treatments, likely reflects the activation of adaptive or compensatory survival pathways, which are typically enhanced in non-BRAF^V600E^ mutant resistant tumor phenotypes, such as MeTRAV used in this study (BRAF^V600D^), with respect to the utilized BRAF^V600E^ MeOV phenotype. At higher concentrations, however, these adaptive mechanisms appear to be overcome, resulting in overt cytotoxicity. In parallel, the greater susceptibility of HaCaT keratinocytes compared with the highly refractory 3T3 fibroblasts further supports the notion that these compounds do not exert indiscriminate toxicity but rather act in a cell type- and molecular context-dependent manner. Differences in proliferative rate, mitochondrial activity, metabolic profile, and stress-response signaling may account for this selective sensitivity. Importantly, these observations collectively strengthen the concept of a concentration- and time-dependent therapeutic window, within which resistant tumor cells are preferentially affected while non-tumoral cells—particularly fibroblastic cells—remain relatively preserved. The existence of such a window is especially relevant in the context of refractory malignancies, where partial pathway modulation may transiently activate adaptive responses, whereas adequate dosing can overcome these mechanisms and restore cytotoxic efficacy. Although further mechanistic studies and validation in 3D models and in vivo systems are required, the present findings provide a solid preclinical rationale for continued development of QPSs-based strategies, including potential topical applications for skin melanoma lesions and systemic approaches for MDR HR-NB. Finally, the integration of chemical characterization with multivariate statistical analysis represents an additional strength of this study. The use of PCA to jointly interpret chemical and biological findings enhances the robustness of the conclusions and confirms that these newly developed QPSs are mechanistically and biologically distinct from previously reported ones, displaying an improved safety–efficacy profile supported by comprehensive and visually integrative analytical tools.

## Figures and Tables

**Table 1:** IC_50_ values of compounds **1**, **3**, and **4** (24, 48, and 72 h exposure) and PLX (72 h exposure), against PLX-R MeOV and MeTRAV cells. The calculation was made using the cytotoxicity data.

Compound	Exposure Time (h)	PLX-R IC_50_ MeOV (µM)	PLX-R IC_50_ MeTRAV (µM)
**1**	24	51.23 ± 42.33 *	59.72 ± 27.23 *
	48	**6.37 ± 3.02**	72.93 ± 37.22 *
	72	**14.89 ± 5.25**	134.0 ± 40.25 *
**3**	24	62.05 ± 14.00 *	59.06 ± 11.03 *
	48	23.73 ± 3.22	87.11 ± 27.62 *
	72	39.45 ± 6.71	99.64 ± V.W. *
**4**	24	38.86 ± 7.13	**41.62 ± 6.24**
	48	25.40 ± 6.90	**38.26 ± 7.00**
	72	36.35 ± 8.13	67.40 ± 10.16 *
Vemurafenib (PLX)	72	**16.86 ± 3.48**	**53.43 ± 20.91** *

V.W. = very wide; * extrapolation provided by the nonlinear model mentioned in the main text. The most interesting results are bolded and in red.

**Table 2:** Comparison of IC_50_ values of compounds **1**, **3**, **4**, and those of etoposide (ETO) in HTLA 230 and MDR HTLA ER neuroblastoma.

Compound	Exposure Time (h)	IC_50_ HTLA 230 (µM)	IC_50_ MDR HATLA ER (µM)
**1**	24	117.70 ± 71.64 *	164.90 ± 38.30 *
	48	**25.02 ± 4.73**	105.60 ± 14.58 *
	72	**4.03 ± 0.91**	**32.71 ± 8.79**
**3**	24	73.47 ± 7.89 *	114.30 ± 12.50 *
	48	57.92 ± 8.95 *	62.03 ± 15.24 *
	72	**24.67 ± 3.29**	**27.76 ± 3.56**
**4**	24	110.30 ± 9.95 *	164.00 ± 56.45 *
	48	97.40 ± 12.72 *	110.20 ± 12.20 *
	72	**47.36 ± 5.81**	56.88 ± 3.09 *
**ETO**	**24**	**145.60 ± 35.40** *	**531.20 ± 147.35** *

* Extrapolation provided by the nonlinear model mentioned in the main text; more interesting results are in red and bold; results from administering ETO for 24 h are in black and bold.

**Table 3:** HC_50_ values of all samples vs. RBCs and their IC_50_ values vs. all cancer cells.

Cells	Sample 1	Sample 3	Sample 4
RBCs °	52.97 ± 10.93 *	33.12 ± 6.91	31.15 ± 4.13
MeOV 24 h	51.23 ± 42.33 *	62.05 ± 14.00 *	38.86 ± 7.13
MeTRAV 24 h	59.72 ± 27.23 *	59.06 ± 11.03 *	41.62 ± 6.24
HTLA 230 24 h	117.70 ± 71.64 *	73.47 ± 7.98 *	110.30 ± 9.55 *
HTLA ER 24 h	164.90 ± 38.30 *	114.30 ± 12.50 *	164.00 ± 56.45 *
MeOV 48 h	6.37 ± 3.02	23.73 ± 3.22	25.40 ± 6.90
MeTRAV 48 h	72.93 ± 37.22 *	87.11 ± 27.62 *	38.26 ± 7.00
HTLA 230 48 h	25.02 ± 4.73	57.92 ± 8.95 *	97.40 ± 12.72 *
HTLA ER 48 h	105.60 ± 15.58 *	62.03 ± 15.24 *	110.20 ± 12.10 *
MeOV 72 h	14.89 ± 5.25	39.45 ± 6.71	36.35 ± 8.13
MeTRAV 72 h	134.0 ± 40.25 *	99.64 ± V.W. *	67.40 ± 10.16 *
HTLA 230 72 h	4.03. ± 0.91	24.67 ± 3.29	46.36 ± 5.81
HTLA ER 72 h	32.71 ± 8.79	27.26 ± 3.56	56.88 ± 3.09 *

° Time of experiment according to the protocol detailed in the experimental Section. V.W. = very wide; * extrapolation.

## Data Availability

The original contributions presented in this study are included in the article/Supplementary Material. Further inquiries can be directed to the corresponding author.

## Supplementary Materials

The following supporting information can be downloaded at: https://www.mdpi.com/article/10.3390/ijms27073170/s1.
