# Supplementary material for: Quaternary Phosphonium Salts Outperformed Vemurafenib (PLX) and Etoposide Against BRAFV600D,V600E PLX-Resistant Melanoma and MDR Neuroblastoma, Exhibiting No/Low Toxicity on 3T3/HaCaT Cells
Source: Int J Mol Sci. 2026 Mar 31;27(7):3170. doi: 10.3390/ijms27073170 (PMC13072898; doi:10.3390/ijms27073170)
Supplement: Supplementary file 1 [file ijms-27-03170-s001.zip › ijms-4166388_SM_R1.pdf]

Supplementary Materials

Quaternary Phosphonium Salts Outperformed Vemurafenib (PLX) and Etoposide Against BRAF$^{V600D, V600E}$ PLX-Resistant, melanoma and MDR Neuroblastoma, exhibiting No/Low Toxicity on 3T3/HaCaT Cells

Silvana Alfei [1,*], Maria Grazia Signorello [1,2], Sara Tirendi [3,4], Elaheh Khaledizadeh[3], Paolo Giordani [1], Caterina Reggio [5], Barbara Marengo [3,6]*, and Cinzia Domenicotti [3,6]

[1] Department of Pharmacy, University of Genoa, Viale Cembrano, 16148 Genoa, Italy; paolo.giordani@unige.it (P.G.)

[2] Department of Pharmacy, Biochemistry Laboratory, University of Genoa, Viale Benedetto XV 3, I-16132 Genova, Italy; mariagrazia.signorello@unige.it (M.G.S.)

[3] Department of Experimental Medicine (DIMES), University of Genova, Via Alberti L.B., 16132 Genoa, Italy; cinzia.domenicotti@unige.it (C.D.), sara.tirendi@edu.unige.it (S.T.)

[4] Inter-University Center for the Promotion of the 3Rs Principles in Teaching & Research (Centro 3R), 56122 Pisa, Italy; Elaheh.khaledizadeh@edu.unige.it (E.K.)

[5] Laboratory of Experimental Therapies in Oncology, IRCCS Istituto Giannina Gaslini, Via G. Gaslini 5, 16147 Genoa, Italy; caterinareggio@gaslini.org (C.R.)

[6] IRCCS Azienda Ospedaliera Metropolitana, Genova, Italia

* Correspondence: alfei@difar.unige.it (S.A.); Tel.: +39 010 355 2296; barbara.marengo@unige.it (B.M.)

Section S1. Copies of ^{1}H, ^{13}C NMR and ^{31}P NMR spectra of Most Significant Compounds of This Study.

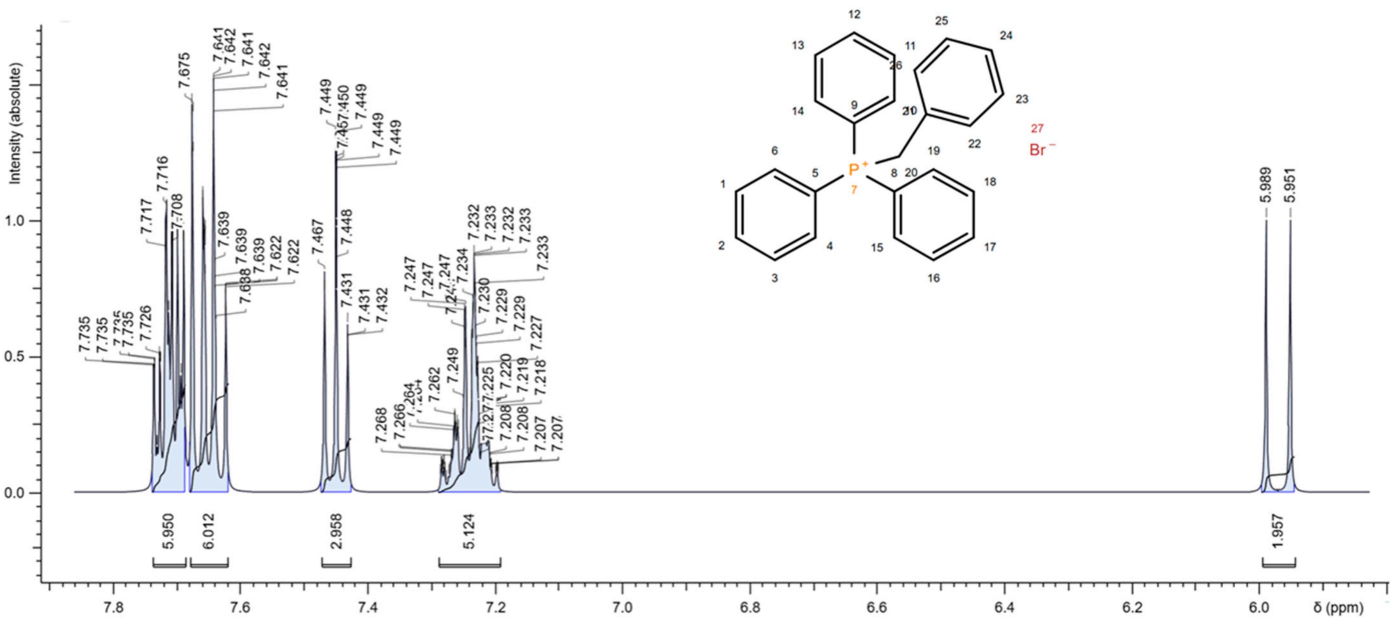

Figure S1.1. ^{1}H NMR spectrum (400 MHz, $CHCl_3$) of compound **1**.

In the ^{1}H NMR spectrum of **1**, two multiplets and a triplet were found in the ranges 7.71-7.65, 7.26-7.22 ppm and at 7.45 ppm. The multiplet at high values comprehended a signal at 7.71 ppm related to six equivalent proton atoms in the TPP group. This signal was attributed to the couples of H-1,3, H-11,13 and H-16,18 according to numbering observable in the structure in Figure S1.1. Each of them presented a J^{o} = 7.38 Hz with H-2, H-12 and H-17 and a J^{o} = 7.73 Hz with the couples H-4,6, H-10,14 and H-15,19, whose signal was detected at 7.65 ppm. An additional $J^{4}_{H\text{-}P}$ of 3.70 Hz was measured. Proton atoms H-4,6, H-10,14 and H-19,15 showed a J^{o} = 7.73 Hz with previously described H-1,3, H-16,18 and H-11,13 and a $J^{4}_{H\text{-}P}$ of 13.30 Hz with phosphor atom. The triplet at 7.45 ppm was attributed at H-2, H-12 and H-17 which were coupled with the H-1,3, H-11,13 and H-16,18 with J^{o} = 7.38 Hz. The multiplet in the region 7.26-7.22 ppm contained a signal at 7.26 ppm assigned to H-23 and H-25. Each of them presented a J^{o} = 7.40 Hz with H-24 and a J^{o} = 7.61 Hz with H-22 and H-26, respectively. Another signal detected at 7.22 was assigned to equivalent H-22 and H-26, which were coupled with J^{o} = 7.61 Hz with equivalent H-23 and H-25 respectively, and with a $J^{4}_{H\text{-}P}$ = 3.30 Hz with phosphor atom. H-24 gave a signal at 7.22 ppm, which was coupled with a J^{o} = 7.40 Hz with H-23 and H-25. The methylene group of benzyl group gave a signal at 5.97 ppm having a $J^{2}_{H\text{-}P}$ of 15.19 Hz.

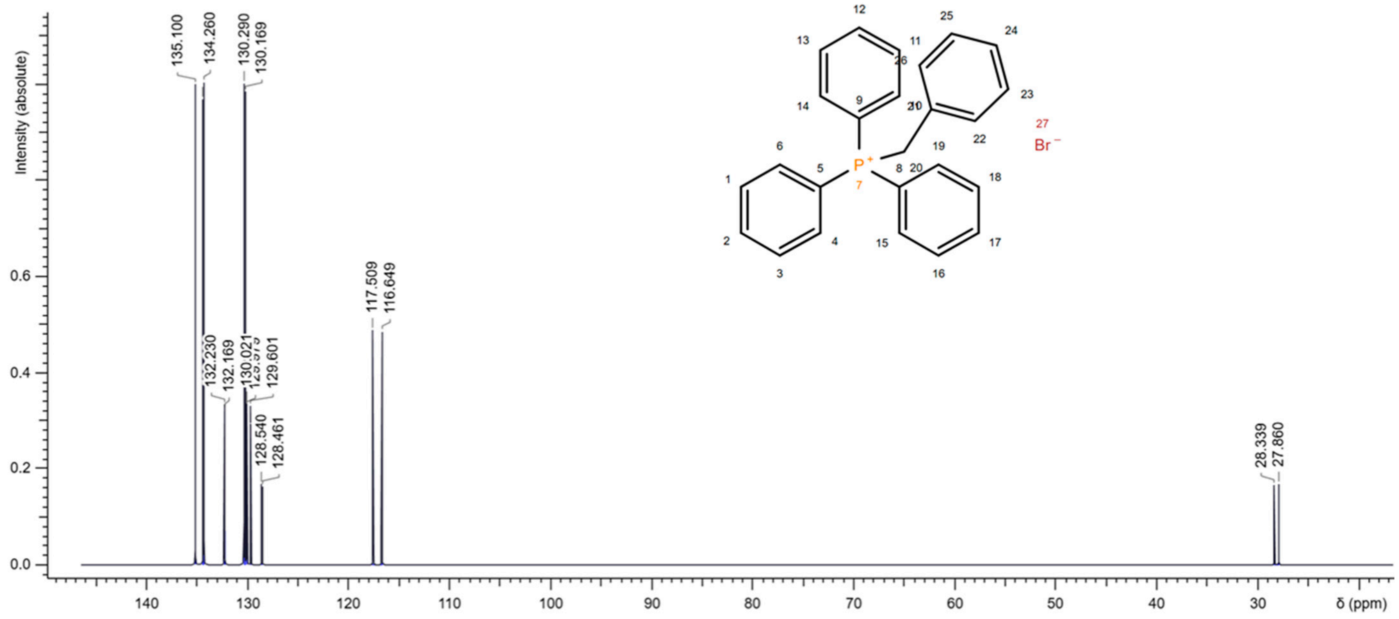

Figure S1.2. ^{13}C NMR spectrum (100 MHz, $CHCl_3$) of compound **1**.

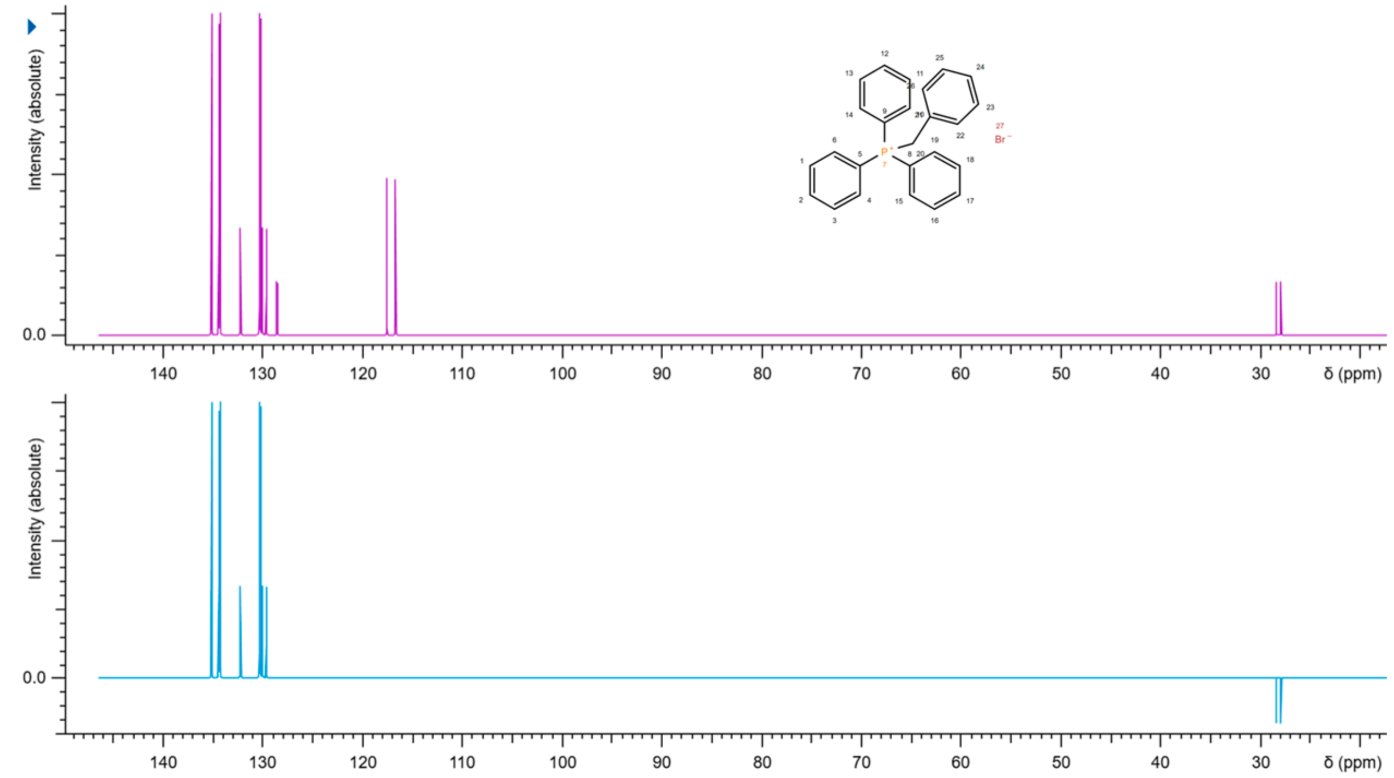

Figure S1.3. ^{13}C NMR spectrum (600 MHz, $CHCl_3$) and DEPT135 experiment of compound **1**.

The ^{13}C NMR spectrum of **1** showed a signal at 135.10 ppm, which was attributed to equivalents C-2, C-12 and C-17, and a signal at 134.31 ppm, which was assigned to the equivalent couples of carbon atoms C-4,6, C-10,14 and C-15,19. These carbons were coupled with phosphorous atom (PA) with a $J^2_{C\text{-}P}$ of 10.20 Hz. The signal at 132.20 ppm was assigned to equivalents C-22 and C-26, which were coupled with PA with a $J^3_{C\text{-}P}$ of 5.39 Hz, while the signal at 130.23 was attributed to the couples of C-1,3, C-13,11 and 18,16, which were coupled with a $J^3_{C\text{-}P}$ of 12.70 Hz with PA. At 130.00 ppm, the signal of the two equivalents C-23 and C-25 coupled with PA with a $J^4_{C\text{-}P}$ of 3.20 Hz was observed. C-24 gave a signal at 129.60 ppm, quaternary C-21 gave a signal at 128.50 ppm, which disappeared in the DEPT135 experiment, coupled with PA with a $J^2_{C\text{-}P}$ of 8.68 Hz. Equivalents quaternary C-5, C-8 and C-9 gave a signal at 117.08 ppm, which disappeared in the DEPT135 experiment, coupled with PA atom with a $J^1_{C\text{-}P}$ of 86.20 Hz. Finally, the C-20 methylene

group gave a signal at 28.10 ppm, which appears upside down in the DEPT135 experiment, coupled with PA with a $J^{1}_{C\text{-}P}$ of 48.10 Hz.

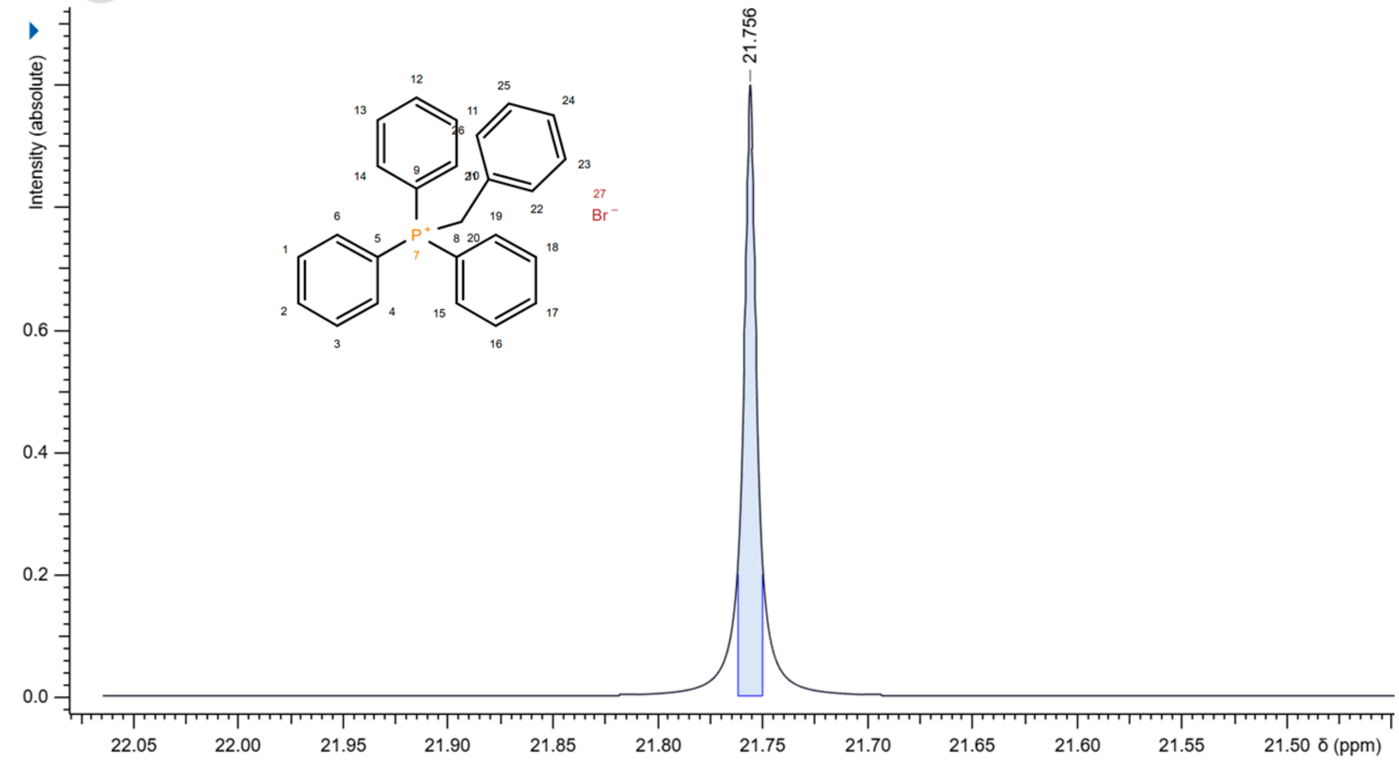

Figure S1.4. ^{31}P NMR spectrum (161 MHz, $CHCl_3$) of compound **1**.

^{31}P NMR spectrum of **1** presented a single peak at +21.76 ppm (s, P) for the phosphonium.

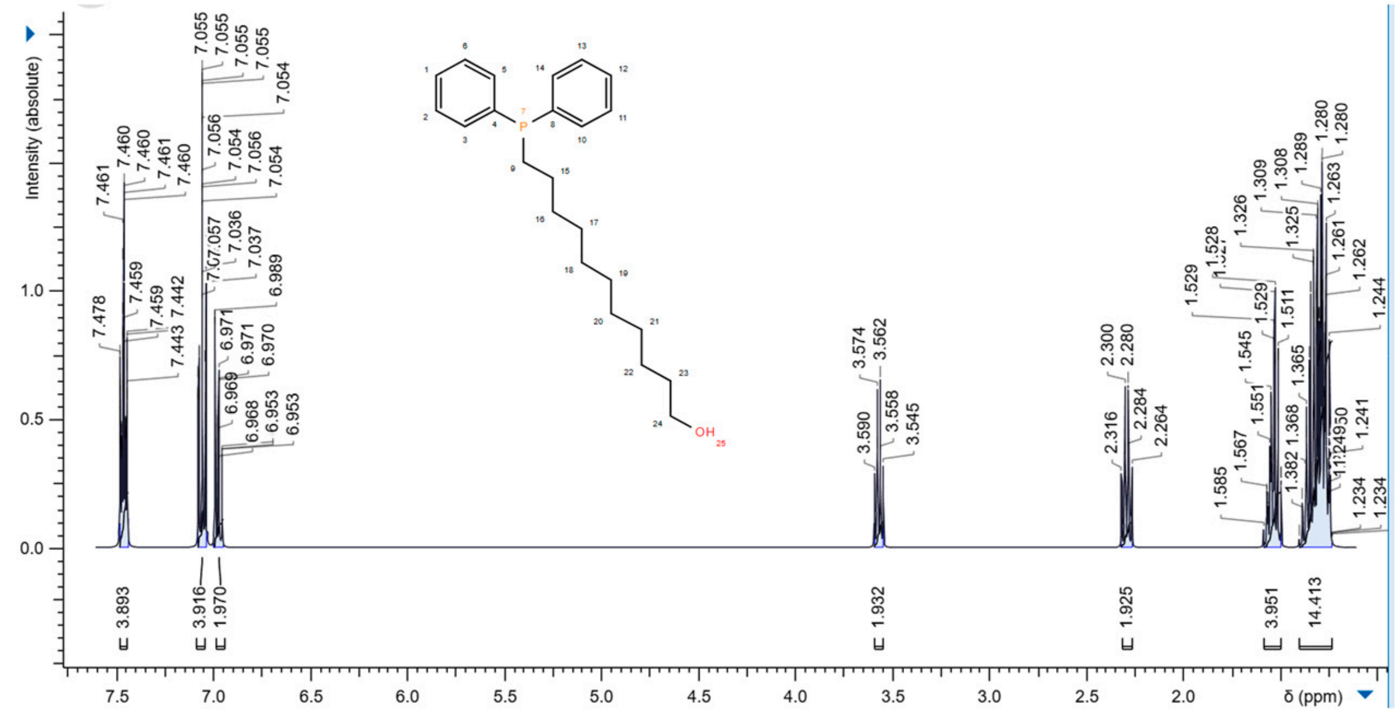

Figure S1.5. ^{1}H NMR spectrum (400 MHz, $CHCl_3$) of compound **2**.

Particularly, in the ^{1}H NMR spectrum of **2**, a signal at 7.46 ppm was observed, belonging to four equivalent proton atoms of the diphenyl system linked to the phosphorous atom (PA). Specifically, this signal was attributed to the couples of H-3,5 and H-10,14 according to numbering observable in the structure in Figure S1.4. Each couple showed a $J^{3}_{H\text{-}P}$ =

7.00 Hz with PA and a J^{o} = 7.50 Hz with H-2,6 and H-11,13, respectively, whose signal was found at 7.05 ppm. These proton atoms in addition to be coupled with protons H-3,5 and H-10,14 were coupled with a lower J^{o} = 7.46 Hz with proton atoms H-1 and H-12, whose signal was detected at 6.97 ppm. Proton atoms H-24 linked to the hydroxyl group (CH_2OH), which were chemically, but not magnetically equivalents, gave a triple doublet signal at 3.57 ppm and showed both a J^{gem} = 5.04 Hz and a $J^{vic.}$ = 6.43 Hz with proton atoms H-23. The complex signal (quintet) of H-23 (1.53 ppm) showed two coupling constants ($J^{vic.}$ of 6.43 and 6.86 Hz), with proton atoms H-24 and H-22, respectively. The signal of proton atoms H-22 was instead found at 1.33 ppm, showing couplings with proton atoms H-23 ($J^{vic.}$ = 6.86 Hz) and H-21 ($J^{vic.}$= 6.80 Hz), whose signal was found at 1.37 ppm. These proton atoms (H-21), in addition to being coupled with proton atom H-22, presented a coupling ($J^{vic.}$ of 7.70 Hz) with proton atom H-20, having signal at 1.28 ppm and in turn coupled with proton atom H-19 (1.27 ppm) with the same $J^{vic.}$ of 7.70 Hz. The same coupling constant ($J^{vic.}$ of 7.70 Hz) was also observed between proton atoms H-19 and H-18 (1.28 ppm) and H-18 and H-17 (1.28 ppm). Proton atoms H-17 were chemically but not magnetically equivalents, thus showing also a J^{gem} = 6.00 Hz and a J^{vic} = 7.54 Hz with protons H-16. These protons were not magnetically equivalents, thus showing a J^{gem} = 4.79 Hz coupling with proton atoms H-15 (J^{vic} = 7.23 Hz), whose signal was found at 1.54 ppm. In addition to being coupled with protons H-16, atoms H-15 showed to be coupled both with PA (J^{3}_{H-P} = 8.82 Hz) and with atoms H-9 (J^{vic} = 6.56 Hz), whose signal was detected at 2.29 ppm and was coupled also with PA with a (J^{2}_{H-P} = 8.00 Hz).

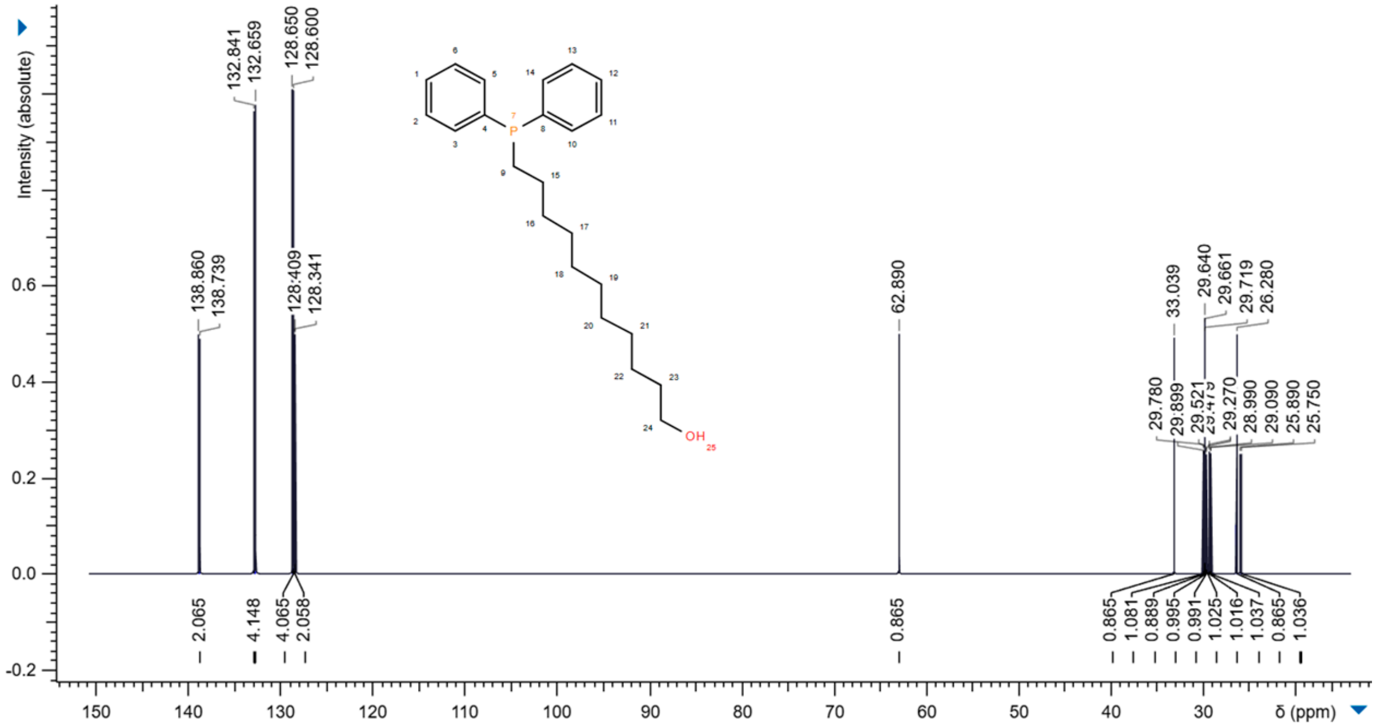

Figure S1.6. ^{13}C NMR spectrum (100 MHz, $CHCl_3$) of compound **2**.

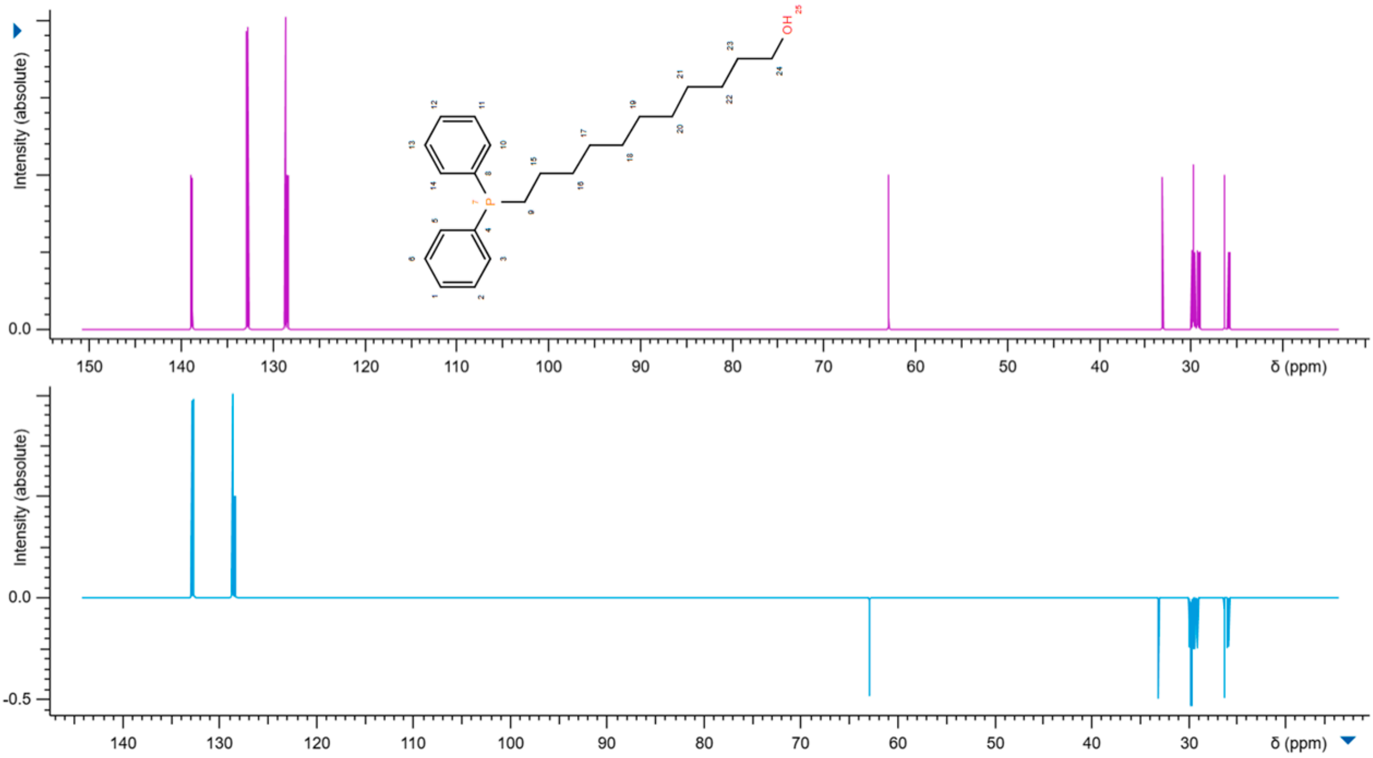

Figure S1.7. ^{13}C NMR spectrum (600 MHz, $CHCl_3$) and DEPT135 experiment of compound **2**.

The ^{13}C NMR spectrum of **2** showed a signal at 138.80 ppm belonging to the equivalents quaternary C-4 and C-8 of diphenyl system, which disappeared in the DEPT135 experiment. The signal was coupled with PA with a $J^{1}_{C\text{-}P}$ = 12.17 Hz. Conversely, the signal at 123.75 ppm was assigned to the equivalents carbon atoms C-3,5 and C-10,14, which resulted coupled with PA with a $J^{2}_{C\text{-}P}$ = 17.76 Hz. The equivalents carbon atoms C-2,6 and C-11,13 gave a signal at 128.62 ppm coupled with PA with a $J^{3}_{C\text{-}P}$ = 6.00 Hz. The last signal of the diphenyl system was detected at 128.38 ppm, which was assigned to the equivalents carbon atoms C-1,12, which presented a $J^{4}_{C\text{-}P}$ coupling constant of 7.00 Hz. The remaining signals were attributed to the carbon atoms of the alkyl chain. The signal of C-24 linked to the hydroxyl group was found at 62.89 ppm. Signals not presenting coupling constants were found at 33.04 (C-23), 29.72 (C-19), 29.66 (C-20, 29.64 (C-21) and 26.28 (C-22) ppm, while signals coupled with PA were found at 29.84 (C-9, $J^{1}_{C\text{-}P}$ = 12.24 Hz), 29.50 (C-18, $J^{5}_{C\text{-}P}$ = 4.97 Hz), 29.25 (C-17, $J^{4}_{C\text{-}P}$ = 5.00 Hz), 29.04 (C-16, $J^{3}_{C\text{-}P}$ = 10.55 Hz) and 25.82 (C-15, $J^{2}_{C\text{-}P}$ = 14.84 Hz) ppm. The signals of all CH_2 groups appeared upside down in DEPT135 experiments.

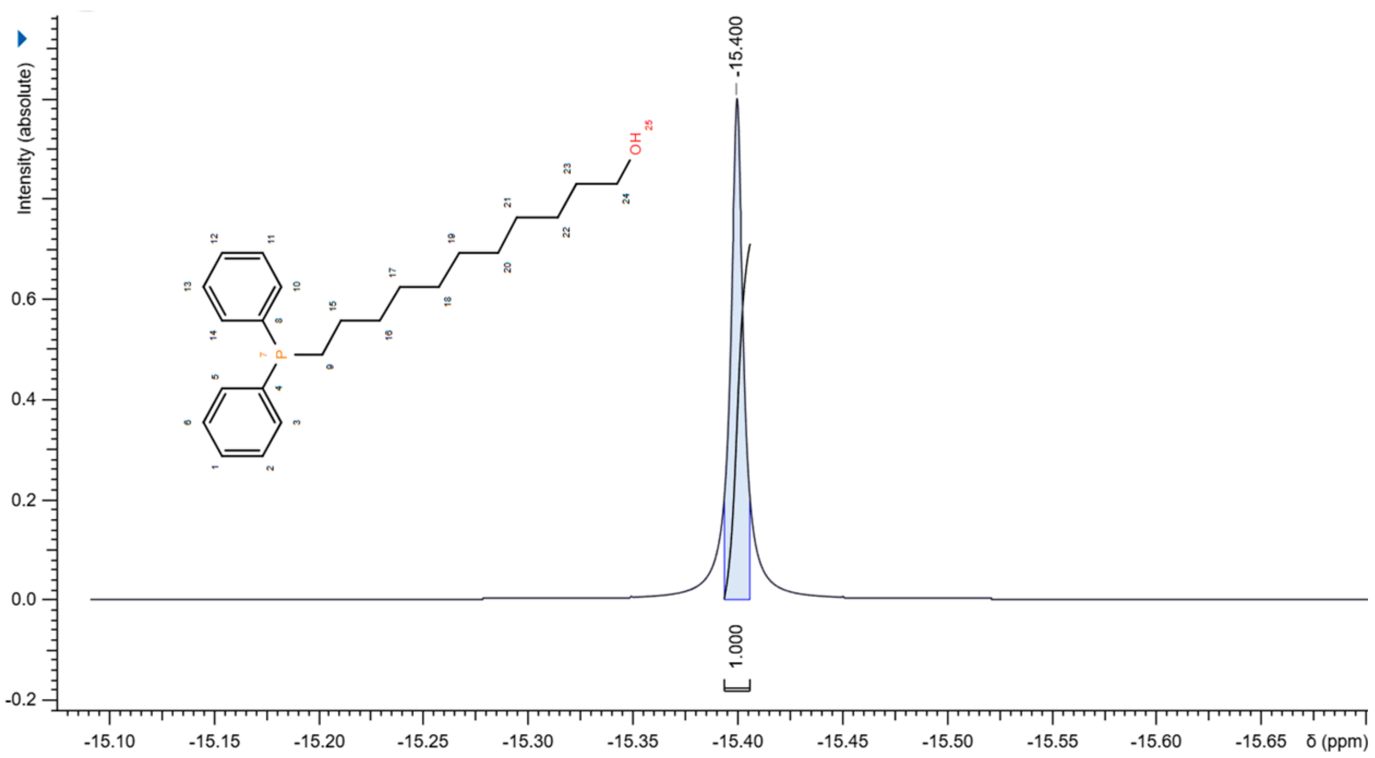

Figure S1.8. ^{31}P NMR spectrum (161 MHz, $CHCl_3$) of compound **2**.

In the ^{31}P NMR spectrum of **2** a single singlet was detected at - 15.40 ppm (s, 1P), according to the common negative signals of substituted PPh_3.

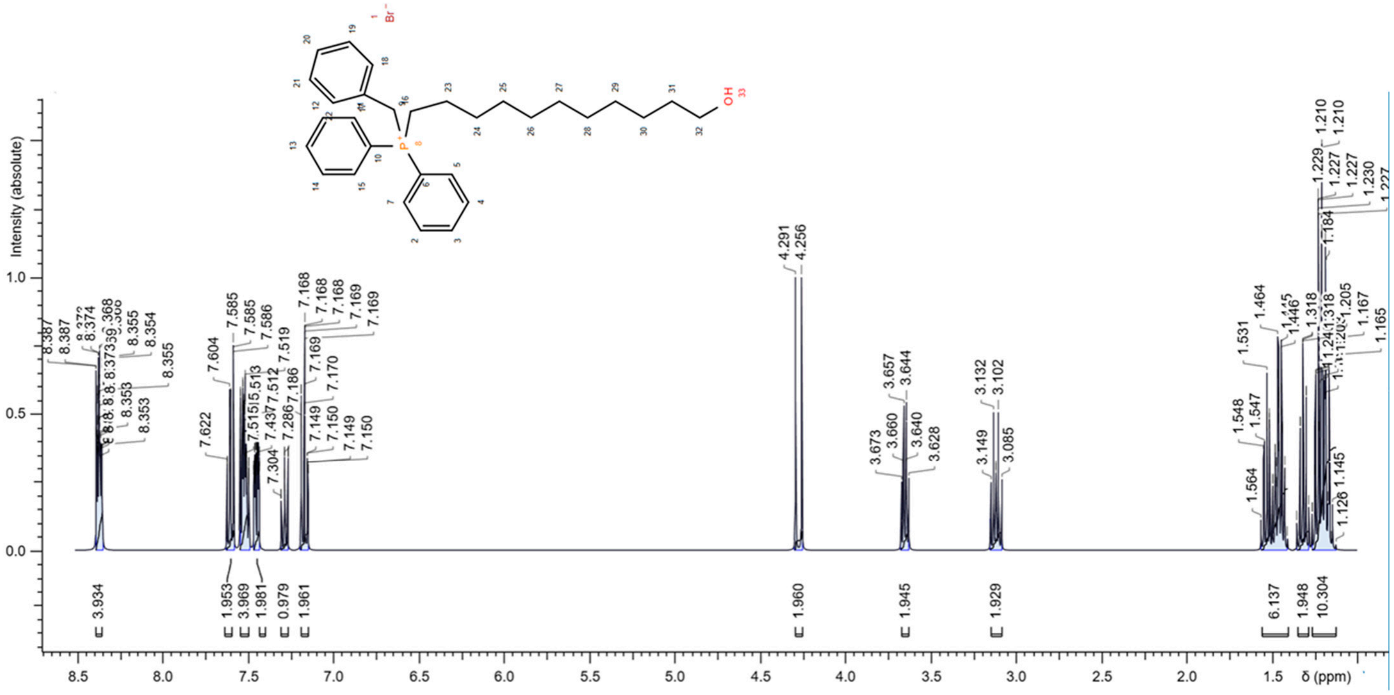

Figure S1.9. ^{1}H NMR spectrum (400 MHz, $CHCl_3$) of compound **3**.

In the ^{1}H NMR spectrum of **3**, a signal at 8.37 ppm was observed, belonging to four equivalent proton atoms of the diphenyl system, linked to the PA. Specifically, this signal was attributed to the couples of H-5,7 and H-11,15 according to numbering observable in the structure in Figure S1.9. Each couple showed a J^o = 7.73 Hz with H-2,4 and H-12,14,

respectively and a $J^{3}_{H\text{-}P}$ = 5.55 Hz with PA. At 7.60 ppm, the signal related to the equivalents H-3 and H-13 was observed. These proton atoms were coupled with H-2,4 and H-12,14, whose signal was detected at 7.52 ppm, with a J^{o} = 7.38 Hz. In addition to the coupling with H-3 and H-13, these proton atoms were coupled with equivalents H-5,7 and H-11,15 (J^{o} = 7.73 Hz) and with PA with a $J^{4}_{H\text{-}P}$ = 5.04 Hz. The signal of H-18 and H-22 of benzyl group was observed at 7.45 ppm, showing couplings with H-19 and H-21 (J^{o} = 7.60 Hz) and with PA ($J^{4}_{H\text{-}P}$ = 3.33 Hz). At 7.28 ppm the triplet of H-20 was observed, which was coupled with H-19 and H-21 (J^{o} = 7.40 Hz), whose signal was instead observed at 7.17 ppm. In addition to the coupling with H-20, these proton atoms showed to be coupled with H-18 and H-22, respectively (J^{o} = 7.60 Hz). The proton atoms of methylene of benzyl group gave a signal at 4.27 ppm, which was coupled with PA with a $J^{1}_{H\text{-}P}$ = 13.90 Hz. The remaining signals were all the methylene groups of the alkyl chain. Specifically, the triple doublet signal at 3.65 ppm was assigned to methylene 32 linked to the hydroxyl (CH_2OH), whose protons were chemically, but not magnetically equivalents, thus showing both a J^{gem} = 5.00 Hz and a $J^{vic.}$ = 6.40 Hz with proton atoms H-31. The complex signal (quintet) of H-31 showed two coupling constants ($J^{vic.}$ of 6.40 and 6.87 Hz), with proton atoms H-32 and H-30, respectively. The quintet signal of proton atoms H-30 was instead found at 1.32 ppm, showing couplings with proton atoms H-31 ($J^{vic.}$ = 6.87 Hz) and H-29 ($J^{vic.}$= 6.80 Hz), whose signal was found at 1.23 ppm. These proton atoms, in addition to being coupled with proton atoms H-30, presented a coupling ($J^{vic.}$ of 7.70 Hz) with proton atoms H-28 with signal at 1.16 ppm. The signals of subsequent proton atoms H-27, 26, 25, 24 and 23 were observed at 1.21, 1.19, 1.23, 1.45 and 1.47 ppm, respectively. Proton atoms H-25, 26, 27 and 28 were coupled with each other with the same $J^{vic.}$ = 7.70 Hz, while not magnetically equivalent protons H-25 showed an additional geminal coupling constant of 6.0 Hz and a new $J^{vic.}$ = 7.50 Hz with H-24, in turn coupled with H- 23 with a $J^{vic.}$ = 7.23 Hz. Proton atoms H-23 presented additional coupling constants with those of methylene H-9 ($J^{vic.}$ = 6.80 Hz) and with PA ($J^{3}_{H\text{-}P}$ = 7.90 Hz).

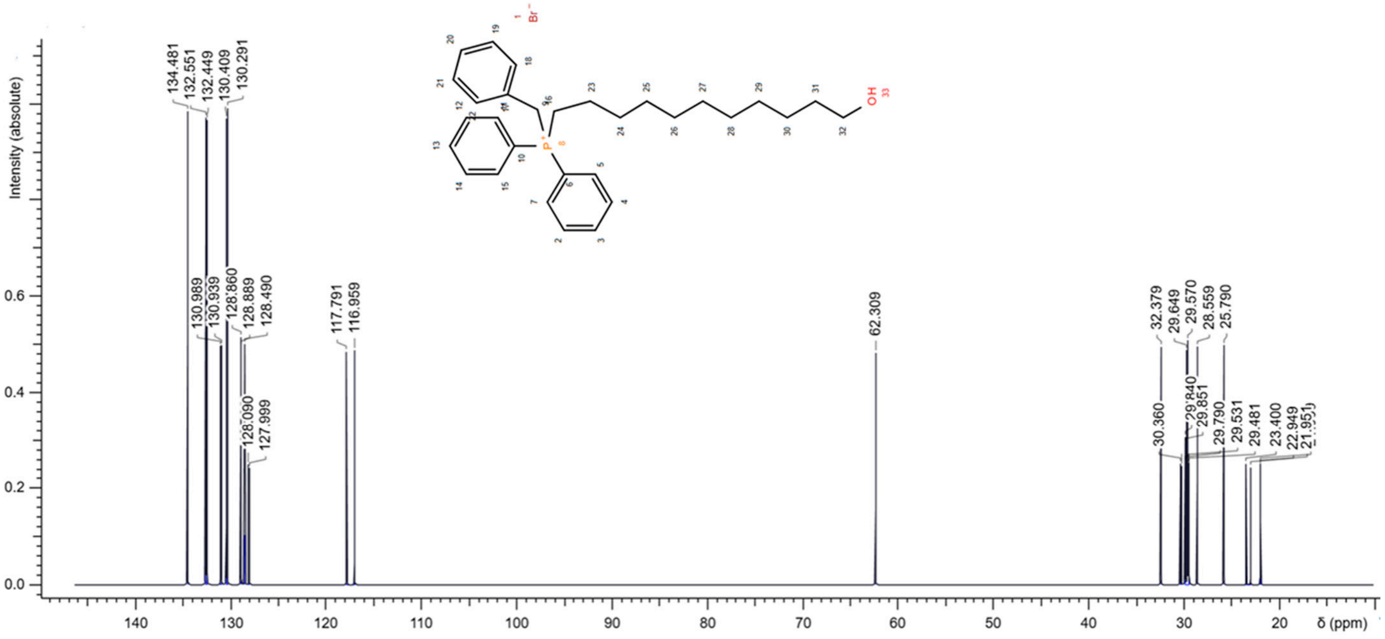

Figure S1.10. ^{13}C NMR spectrum (100 MHz, $CHCl_3$) of compound **3**.

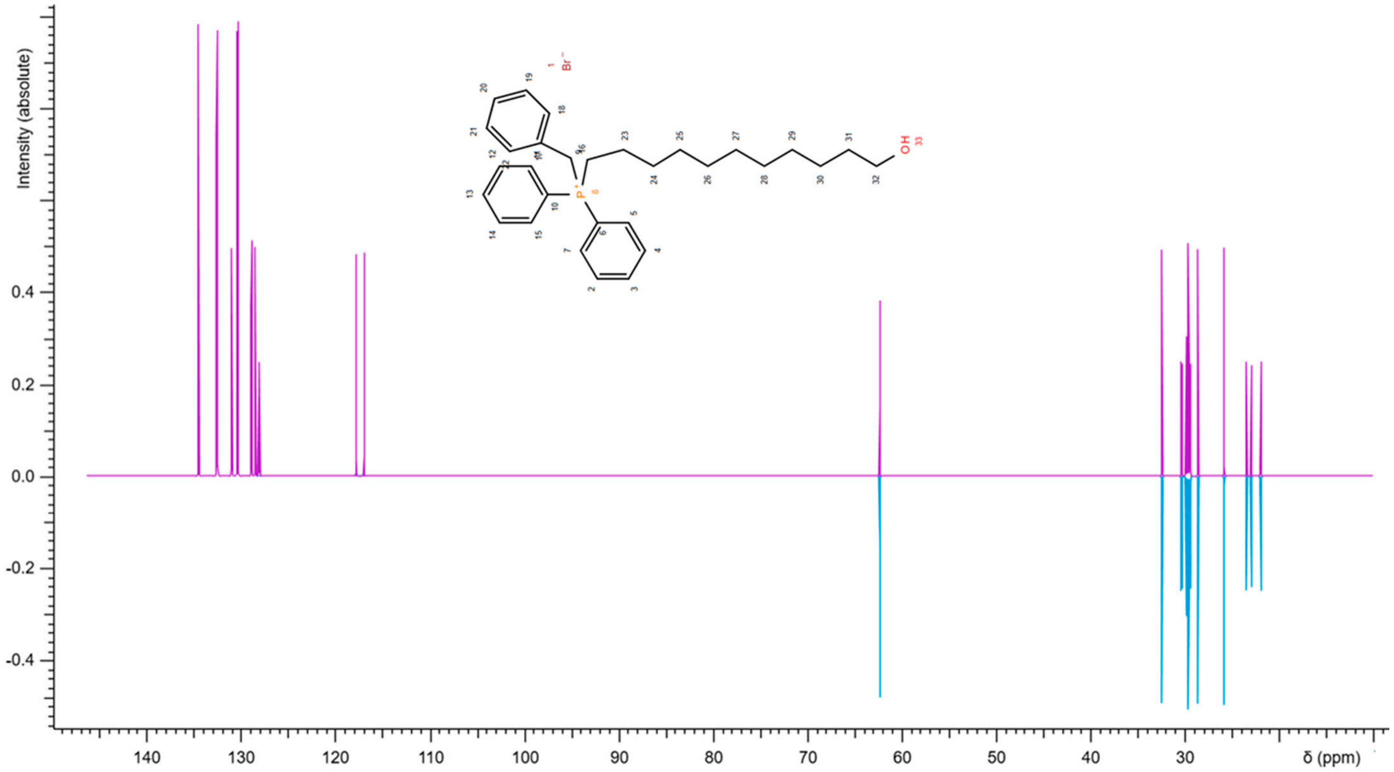

Figure S1.11. ^{13}C NMR spectrum (600 MHz, $CHCl_3$) and DEPT135 experiment of compound **3**.

The ^{13}C NMR spectrum of **3** showed a signal at 134.5 ppm belonging to the equivalents C-3 and C-13 of diphenyl system. The signal coupled with PA (J^2_{C-P} = 10.38 Hz) at 132.5 ppm was assigned to equivalents C-5,7 and C-11,15, while that at 130.96 ppm, coupled with PA too (J^3_{C-P} = 5.30 Hz), was assigned at equivalents C-18 and C-22. Equivalent carbon atoms C-2,4 and C-12,14 gave a signal at 130.4 and were coupled with PA with a J^3_{C-P} = 12.60 Hz. The signal at 128.9 ppm was attributed to equivalent carbon atoms C-19 and C-21, which were coupled with PA with a J^4_{C-P} = 3.20 Hz. The singlet at 128.5 ppm belonged to C-20, while the signal at 128.1 ppm, coupled with PA (J^2_{C-P} = 8.90 Hz), was attributed to the quaternary C-17, which disappeared in the DEPT135 experiment, like the signal of equivalents quaternary C-6 and C-10 observed at 117.4 ppm, which were coupled with PA with a J^1_{C-P} = 83.70 Hz. Singlet signals for carbon atoms of methylene groups in the alkyl chain numbered as C-32, C-31, C-28, C-29, C-27 and C-30 were observed at 62.30 (CH_2OH), 32.40, 29.70, 29.60, 28.60 and 26.80 ppm, respectively. Conversely, the signals of carbon atoms C-24, C-25, C-26, C-16, C-9 and C-23, of methylene groups coupled with PA were detected at 30.30 (J^3_{C-P} = 13.85 Hz), 29.8 (J^4_{C-P} = 5.00 Hz), 29.5 (J^5_{C-P} = 4.97 Hz), 29.6 (J^1_{C-P} = 47.30 Hz), 23.2 (J^1_{C-P} = 45.20 Hz) and 21.9 (J^2_{C-P} = 4.05 Hz). The signals of all CH_2 groups appeared upside down in DEPT135 experiments.

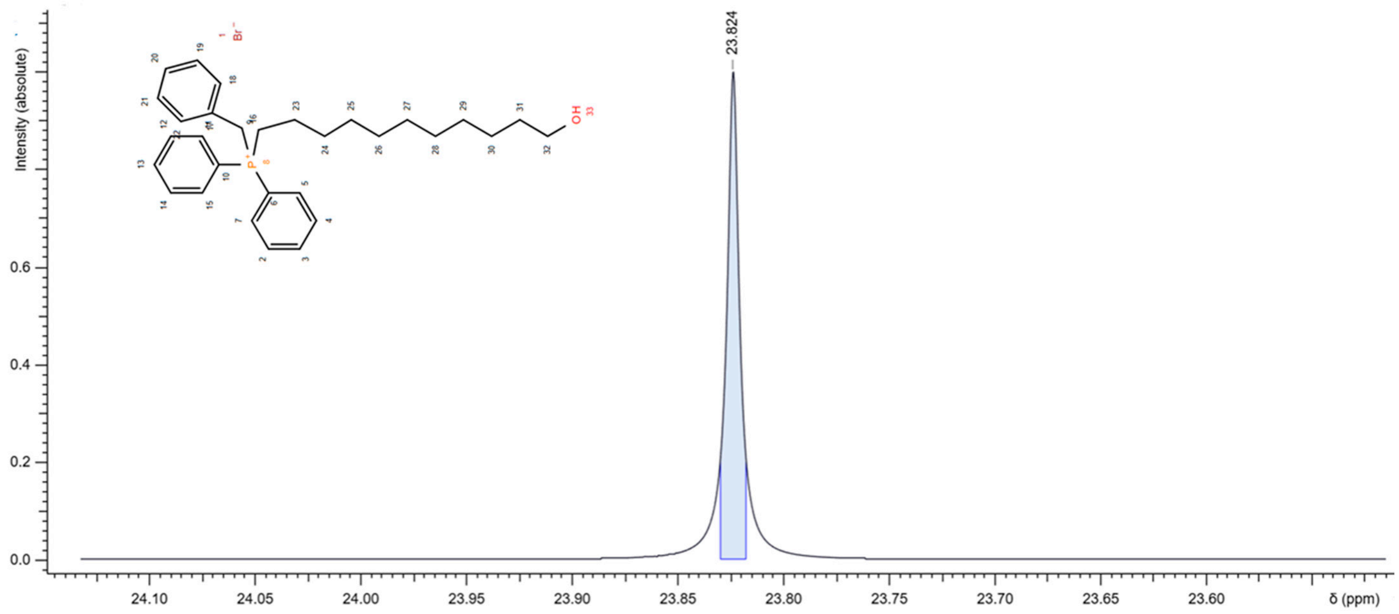

Figure S1.12. ^{31}P NMR spectrum (161 MHz, $CHCl_3$) of compound **3**.

In the ^{31}P NMR spectrum of **3** a single singlet was detected at +23.82 ppm (s, 1P, phosphonium).

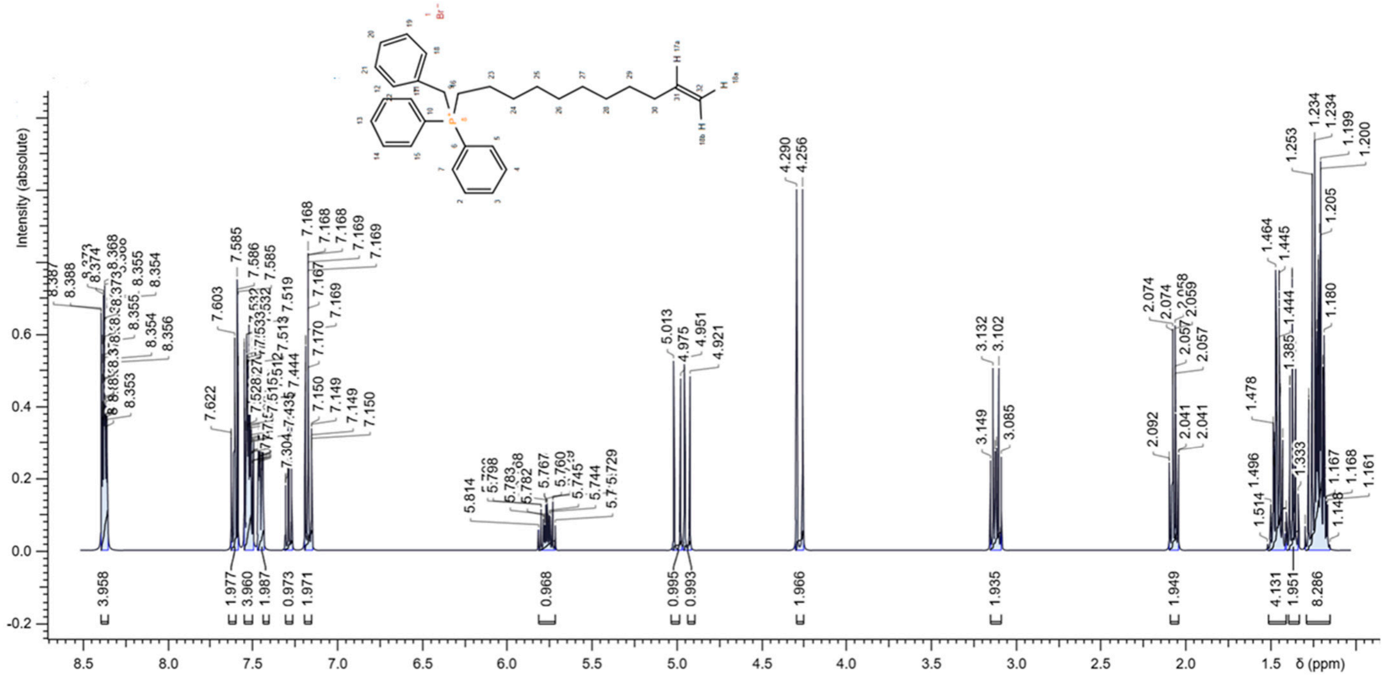

Figure S1.13. ^{1}H NMR spectrum (400 MHz, $CHCl_3$) of compound **4**.

In the ^{1}H NMR spectrum of **4**, the same signals observed for compound **3** at 8.37, 7.60, 7.52, 7.45, 7.28, 7.17 and 4.27 ppm were observed for the same proton atoms, with the same multiplicity and coupling constants. New signals, peculiar to vinyl system, were observed at 5.76, 4.99 and 4.94 ppm. Particularly, the double-double triplet signal at 5.76 ppm was assigned to the proton atom of CH=CH_2 system, numbered as 17a, which was coupled with proton atoms Ha and Hb, numbered as 18a and 18b (CH=CH_2, 4.99, 4.94 ppm), with a J^{cis} = 12.08 Hz and J^{trans} = 15.30 Hz, respectively. Proton 17a was also coupled with not magnetically equivalent proton atoms H-30 with different J^{vic} = 6.43 and 6.30 Hz. Moreover, the proton atoms 18a and 18b were in turn coupled with H-17a with J^{cia} = 12.08 Hz and J^{trans} = 15.30 Hz. Proton atoms H-30 gave their signal at 2.07 ppm and were also coupled with proton atoms H-29 with a J^{vic} = 6.93 Hz. Additionally,

they showed a large geminal coupling constant of 16.70 Hz, while proton atoms H-29 were also coupled with H-28 (J^{vic} = 6.88 Hz). In addition to this coupling, proton atoms H-28, which gave their signal at 1.24 ppm, since not magnetically equivalents, showed a small geminal coupling constant of 6.00 Hz, and a vicinal coupling constant of 7.54 Hz with H-27 (1.19 ppm), in turn coupled with proton atoms H-26 (J^{vic} = 7.70 Hz). The signal of proton atoms H-26 was observed at 1.20 ppm and was coupled both with proton atoms H-27 and H-25 with the same J^{vic} = 7.70 Hz. Like proton atoms H-28, H-25 ones (1.25 ppm) were not magnetically equivalent, thus showing also a geminal coupling of 6.00 Hz and a J^{vic} = 7.54 Hz with proton atoms H-24 (1.45 ppm), which were in turn coupled with protons H-23 (1.47 ppm) with a J^{vic} = 7.23 Hz. In addition to this coupling constant, H-23 showed a J^{vic} = 6.80 Hz with protons H-9 and a $J^{3}_{H\text{-}P}$ = 7.90 Hz with PA. Finally, proton atoms H-9 at 3.12 ppm were also coupled with PA ($J^{3}_{H\text{-}P}$ = 7.90 Hz).

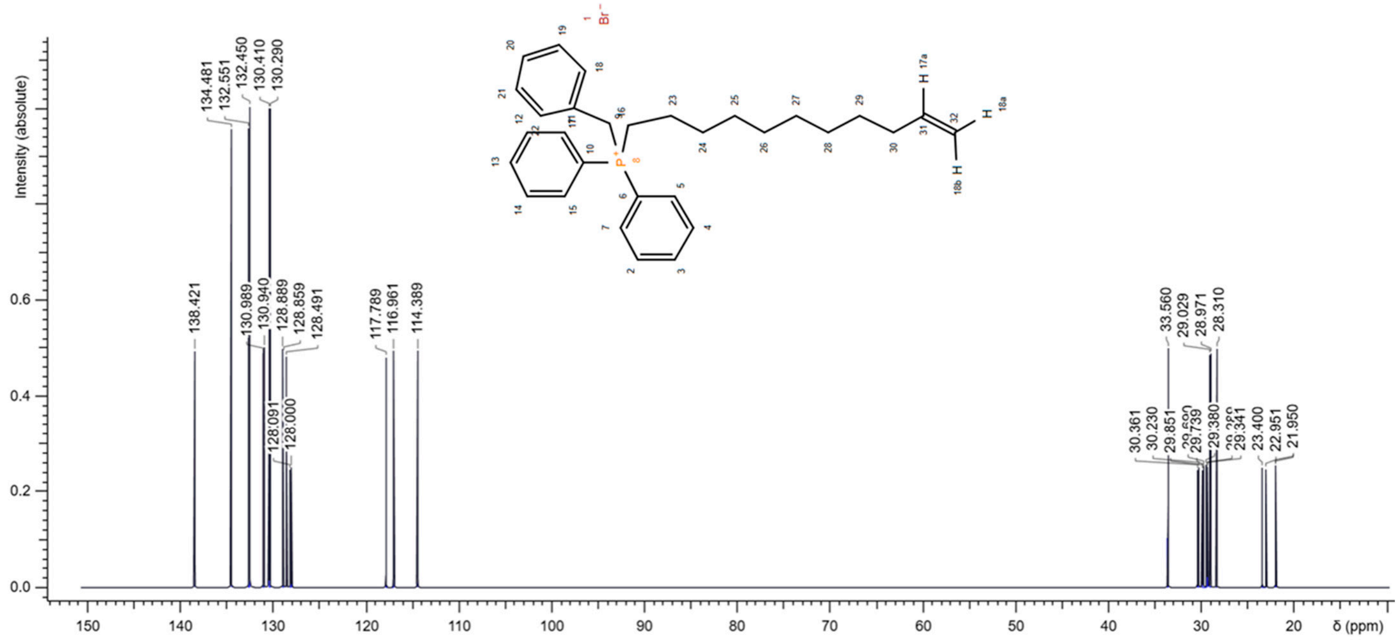

Figure S1.14. ^{13}C NMR spectrum (100 MHz, $CHCl_3$) of compound **4**.

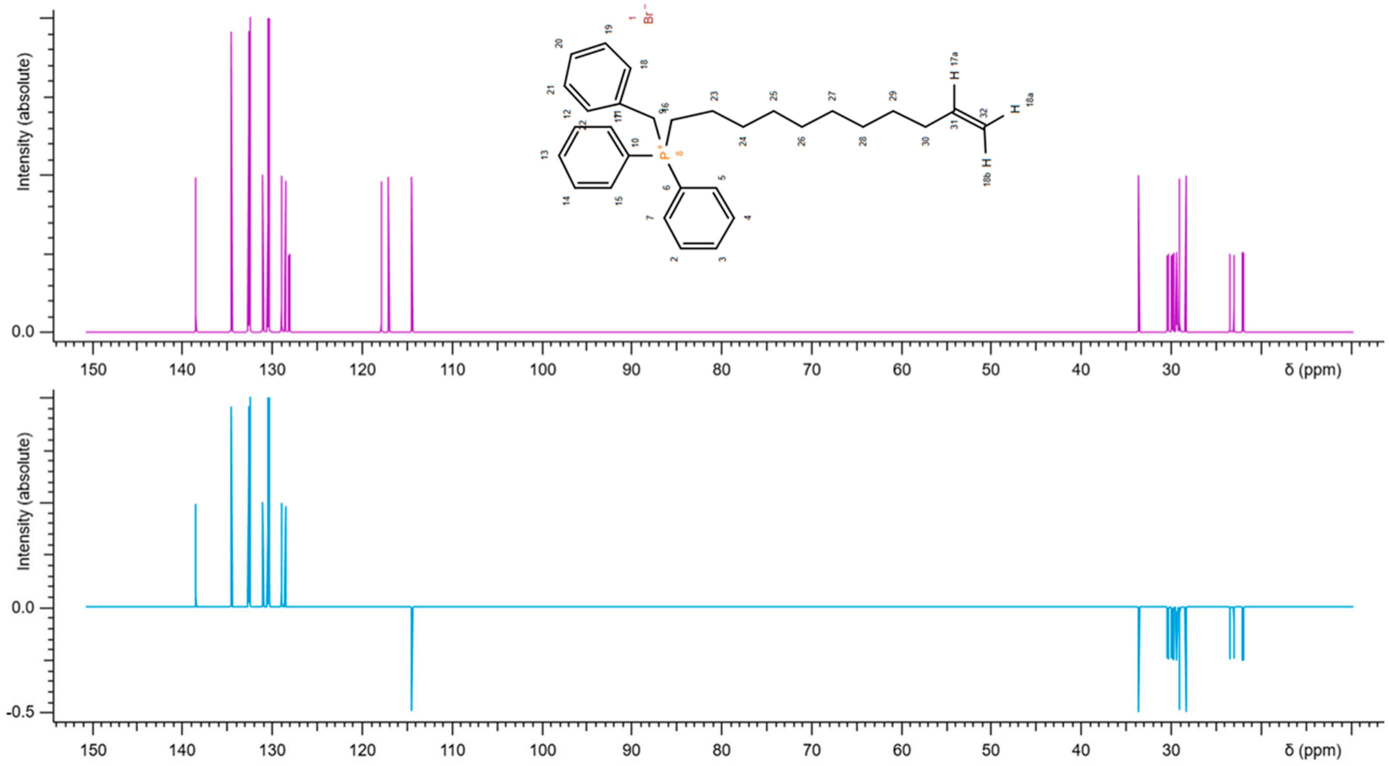

Figure S1.15. ^{13}C NMR spectrum (600 MHz, $CHCl_3$) and DEPT135 experiment of compound **4**.

As for the ^{1}H NMR spectrum, also the ^{13}C NMR spectrum of **4** showed the same signals of **3** for the same carbon atoms and with the same coupling constants with PA (134.5, 132.5, 130.96, 130.4, 128.9, 128.5, 128.1, and 117.4 ppm). Signals of quaternary C- 6, C-10 and C-17 disappeared in the DEPT135 experiment. New singlet signals were observed for the vinyl system at 138.4 and 114.4 ppm, which were attributed at C-31 (CH=CH_2) and C-32 (CH=CH_2), respectively. Singlet signals were also observed for carbon atom C30, C-29, C-28 and C-27 at 33.6, 29.0, 29.0, and 28.3 ppm, since they were not coupled with PA. Conversely carbon atoms C-24 (30.3 ppm), C-25 (29.7 ppm), C-16 (29.6 ppm), C-26 (29.3 ppm), C-9 (23.2 ppm) and C-23 (21.9 ppm) were coupled with PA, thus showing $J^3_{C\text{-}P}$ = 13.85 Hz, $J^4_{C\text{-}P}$ = 5.00 Hz, $J^1_{C\text{-}P}$ = 47.30 Hz, $J^5_{C\text{-}P}$ = 4.97 Hz, $J^1_{C\text{-}P}$ = 45.20 Hz and $J^2_{C\text{-}P}$ = 4.05 Hz. The signals of all CH_2 groups appeared upside down in DEPT135 experiments.

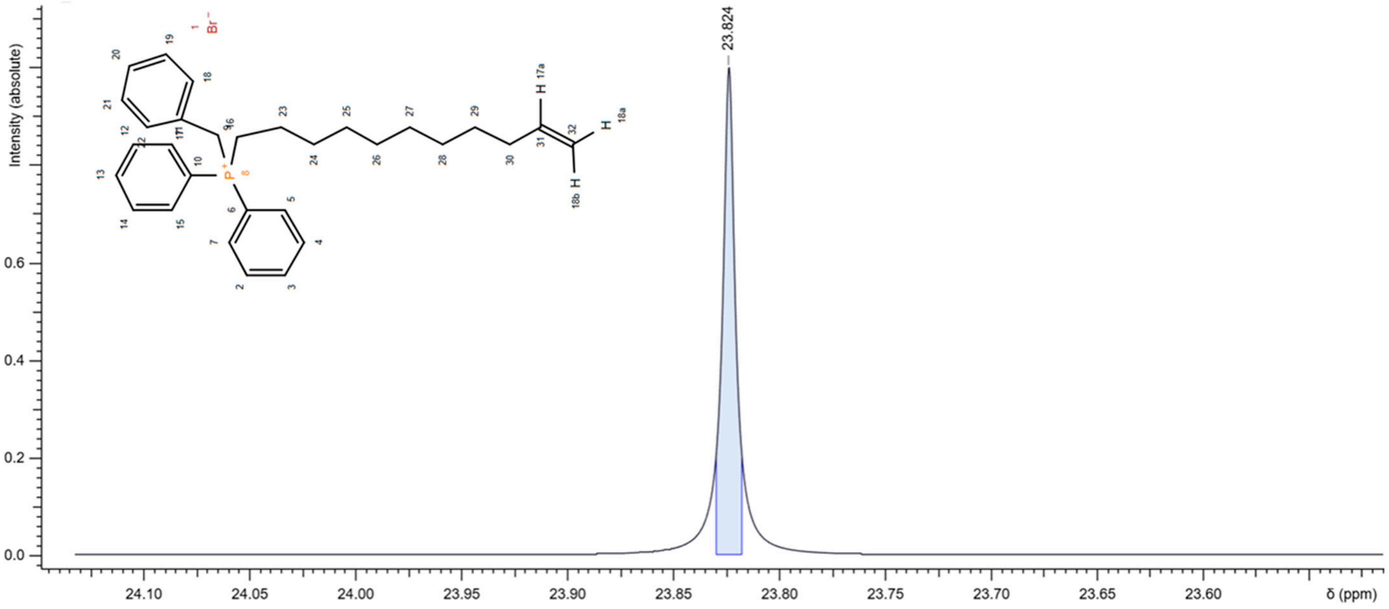

Figure S1.16. ^{31}P NMR spectrum (161 MHz, $CHCl_3$) of compound **4**.

In the ^{31}P NMR spectrum of **4** a single singlet was detected at +23.82 (s, 1P, phosphonium).

Section S2. ATR-FTIR of Compounds **1-4**.

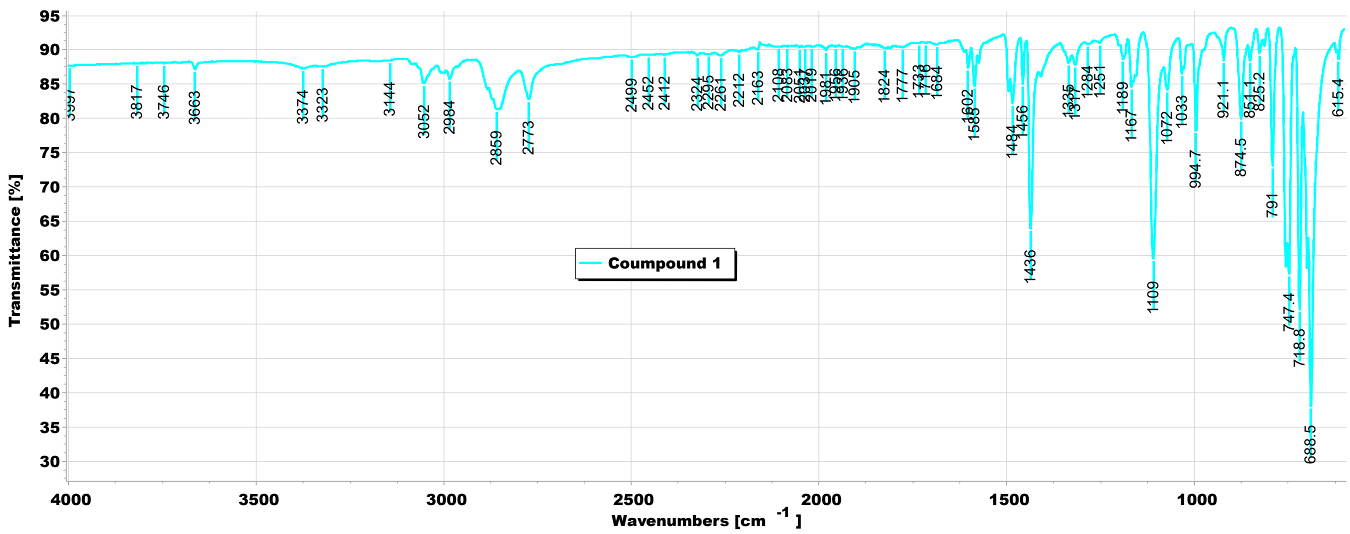

Figure S2.1. ATR-FTIR spectrum of **1**.

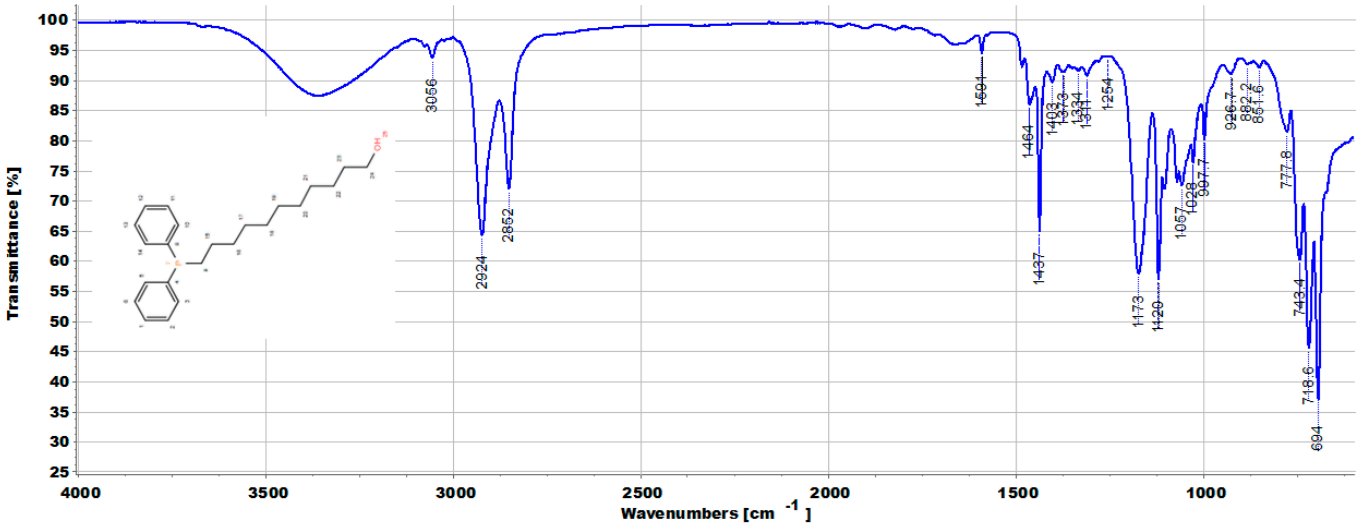

Figure S2.2. ATR-FTIR spectrum of **2**.

The spectrum of **2** showed the large band of the hydrogen bonded O-H stretching at > 3300 cm^{-1} while the =C-H stretching gave bands at 3077, 3056, 3026 and 3012 cm^{-1}. No redshift was observed, probably due to the absence of TPP group and traditional bands at 2924 and 2852 cm^{-1} were found for the symmetric and antisymmetric C-H stretching of methylene groups. The C-H banding gave bands at 1474 and 1437 cm^{-1} while the C-O stretching bands were observed at 1173 and 1120 cm^{-1}. Finally, the bands at 743, 719 and 694 cm^{-1} were attributed to the C-P stretching vibration.

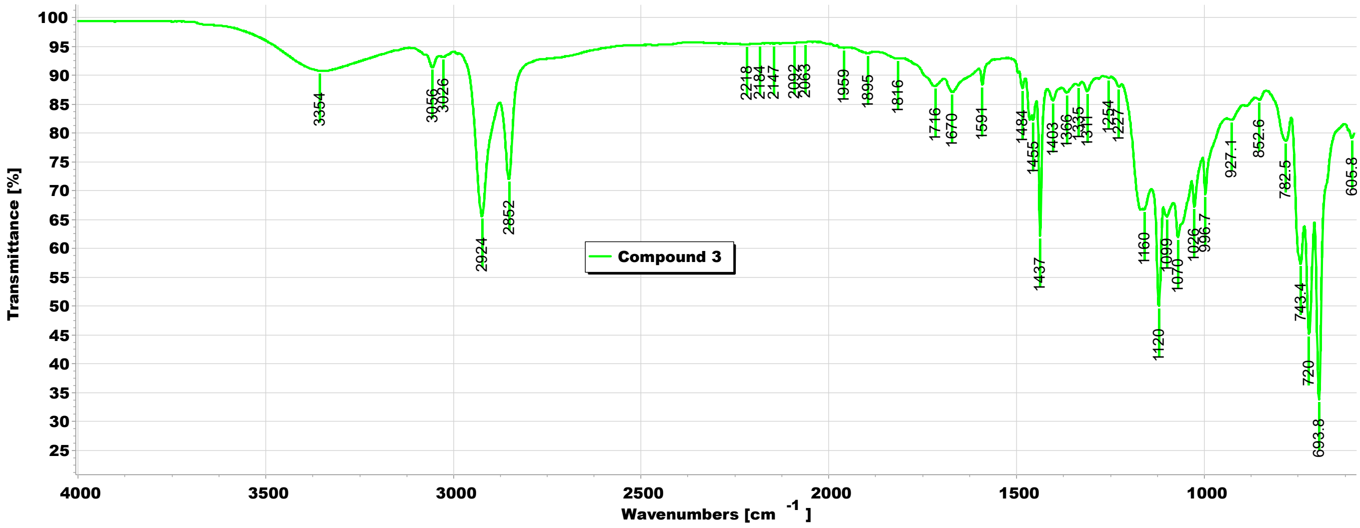

Figure S2.3. ATR-FTIR spectrum of **3**.

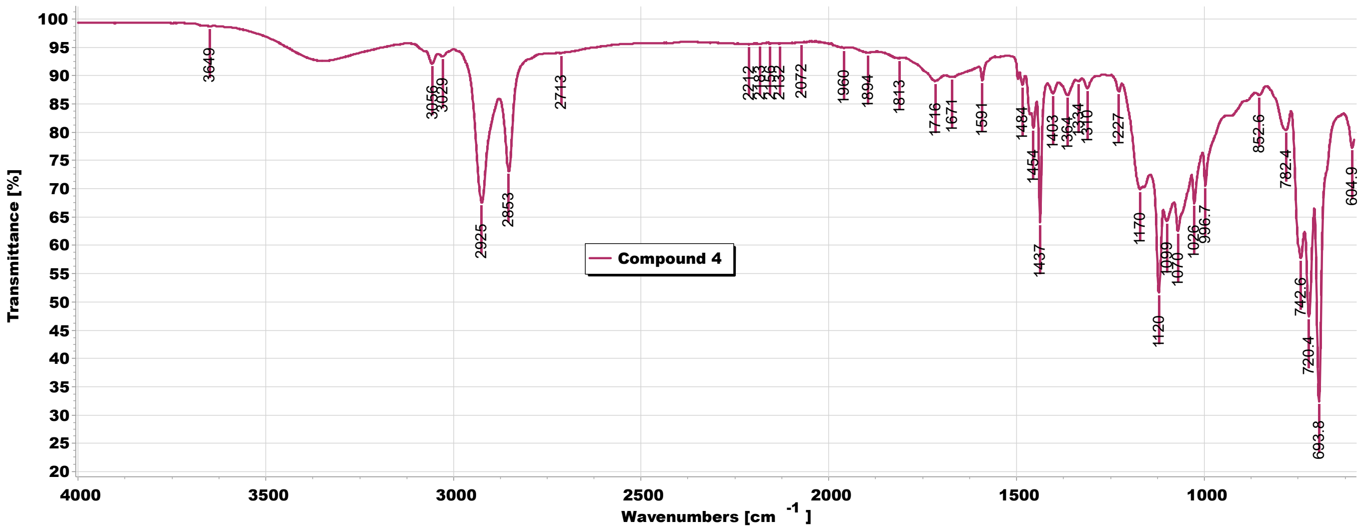

Figure S2.4. ATR-FTIR spectrum of **4**.

As for **2**, compounds **3** and **4** not having the TPP group did not exhibit red-shifted bands for the C-H stretching but traditional bands at 2924/25 and 2852/53 cm^{-1}. While **4** did not give band related to the presence of a hydroxyl, **3** provided a large band at 3354 cm^{-1} (hydrogen bonded O-H stretching) and bands at 1168, 1160 and 110 cm^{-1} (C-O stretching). Both compounds gave bands of the C-H banding at 1454/55 and 1437 cm^{-1}, which the C-P stretching vibration was observed at 743, 720 and 693/94 cm^{-1}.

Section S3. UV-Vis Spectra of Compounds **1-4**

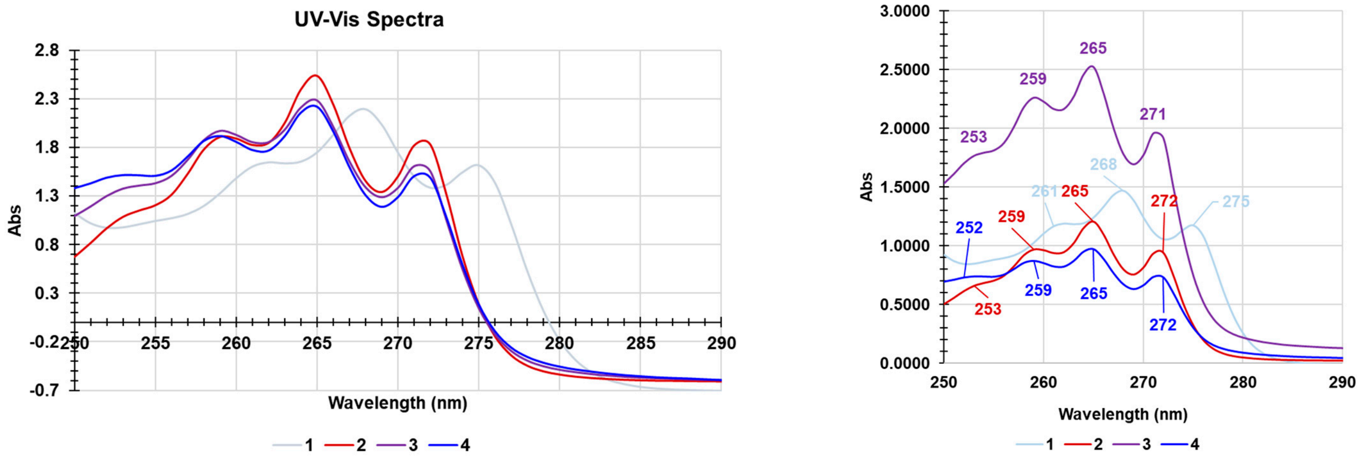

(**a**) (**b**)

Figure S3.1. UV-Vis spectra of compounds **1-4** achieved reporting in graph the standardized csv data provided by the spectrometer using Microsoft 365 Excel software (a) and the not standardized one with peak labels added (b).

Section S4. PCA Results

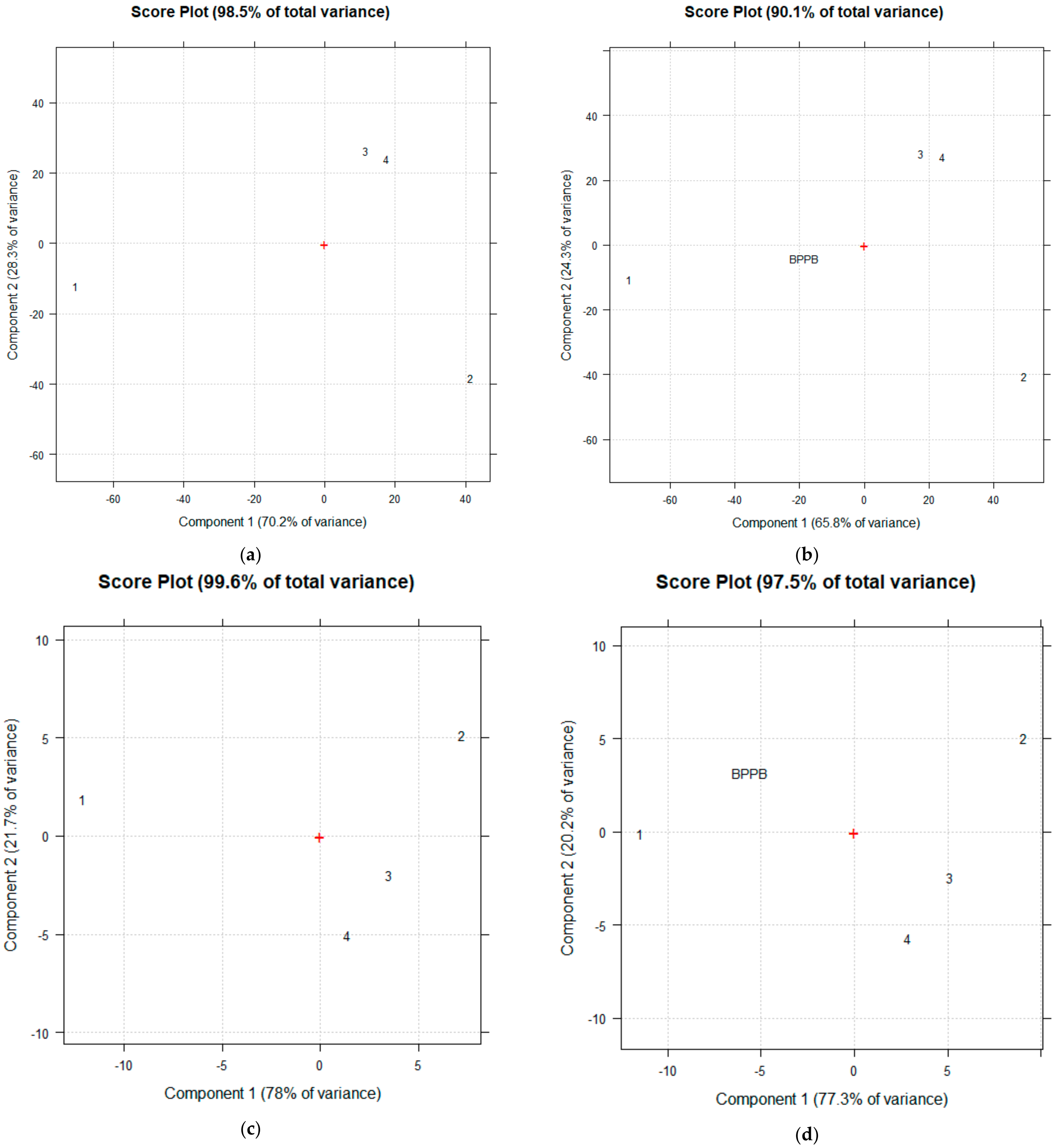

Figure S4.1. Score plot of PC1 vs. PC2 obtained processing matrix A (a), matrix B (b), matrix C (c) and matrix D (d) by PCA.

Section S5. Optical images

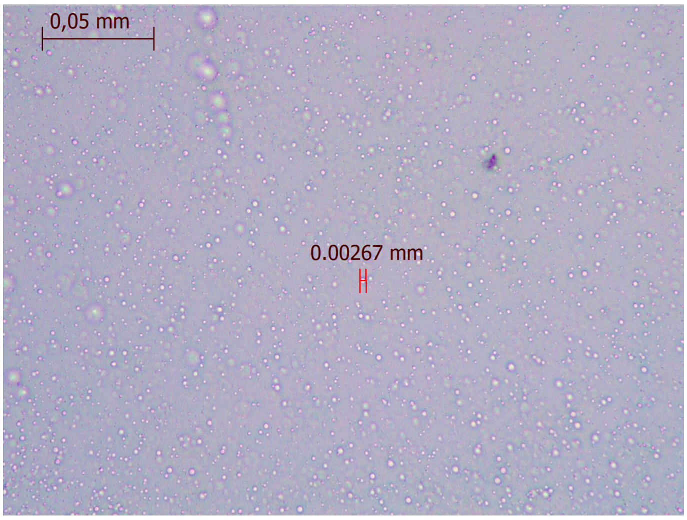

(A)

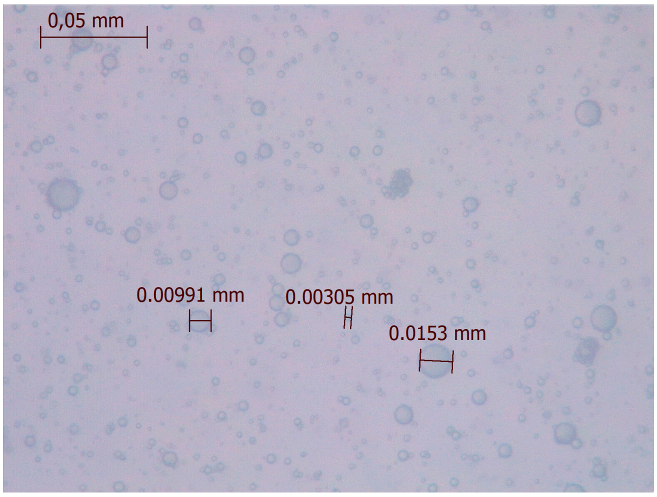

(B)

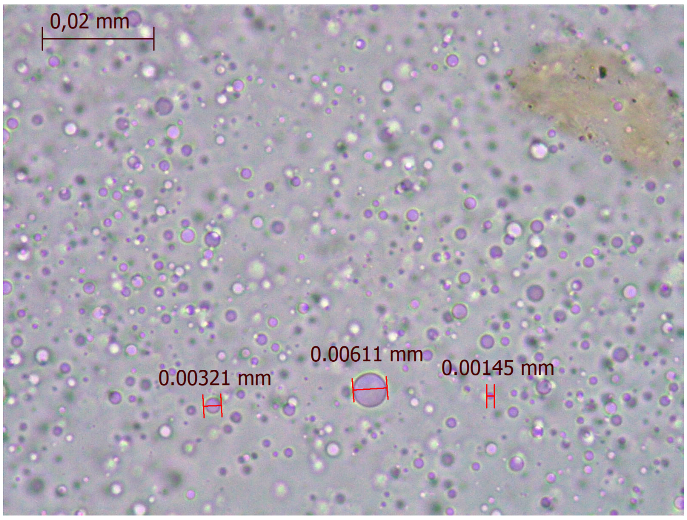

(C)

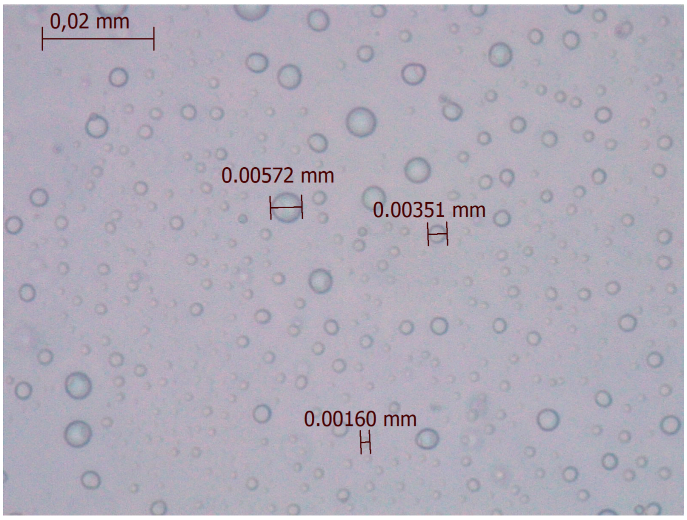

(D)

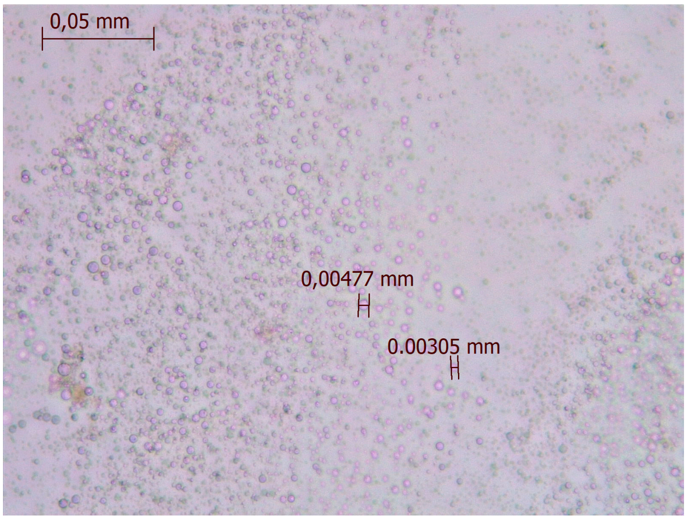

(E)

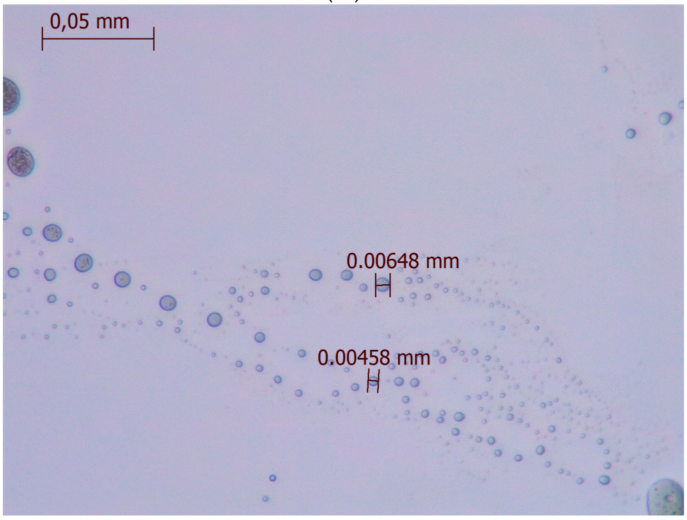

(F)

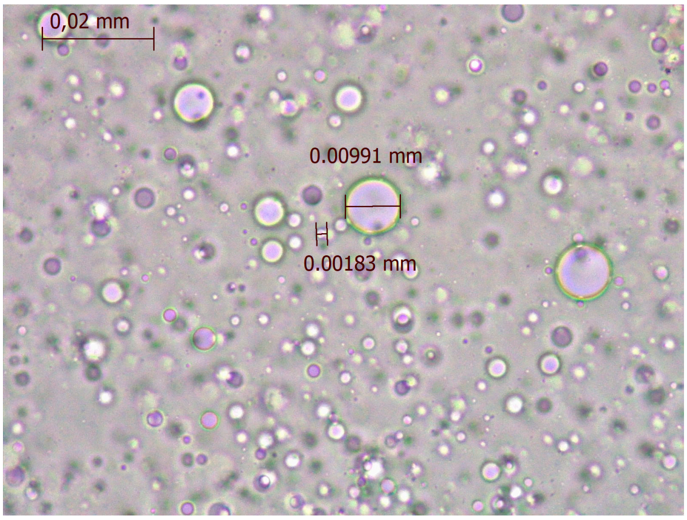
0,02 mm
0.00991 mm
0.00183 mm

(G)

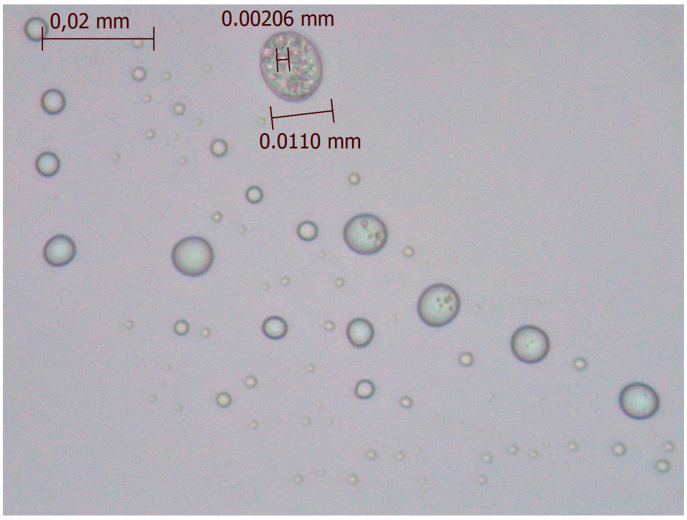
0,02 mm
0.00206 mm
0.0110 mm

(H)

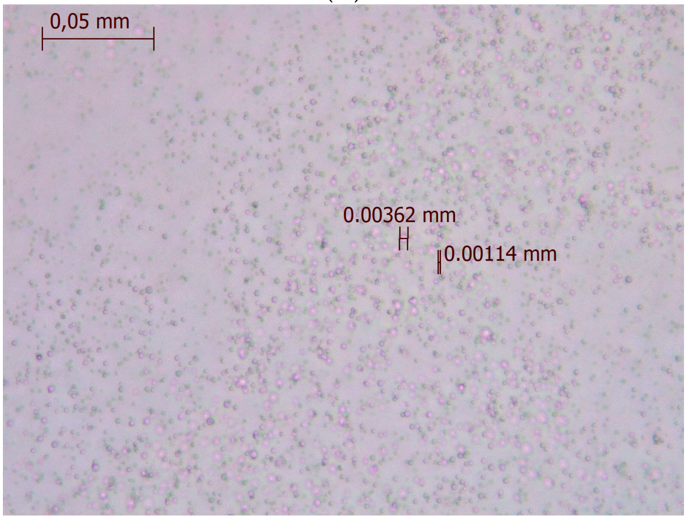
0,05 mm
0.00362 mm
0.00114 mm

(I)

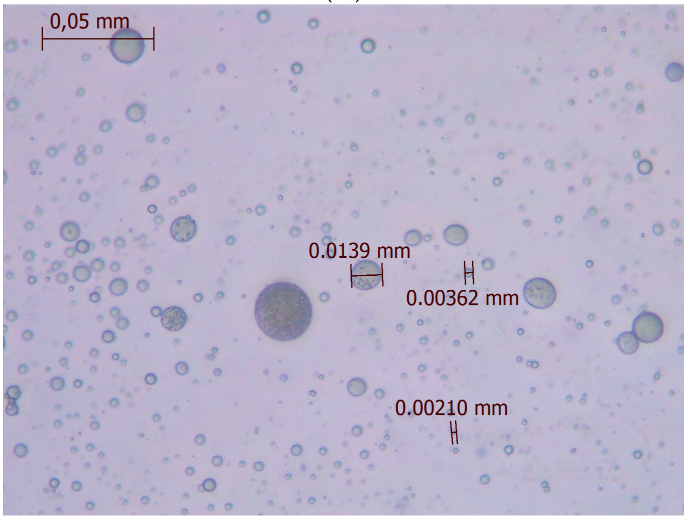
0,05 mm
0.0139 mm
0.00362 mm
0.00210 mm

(J)

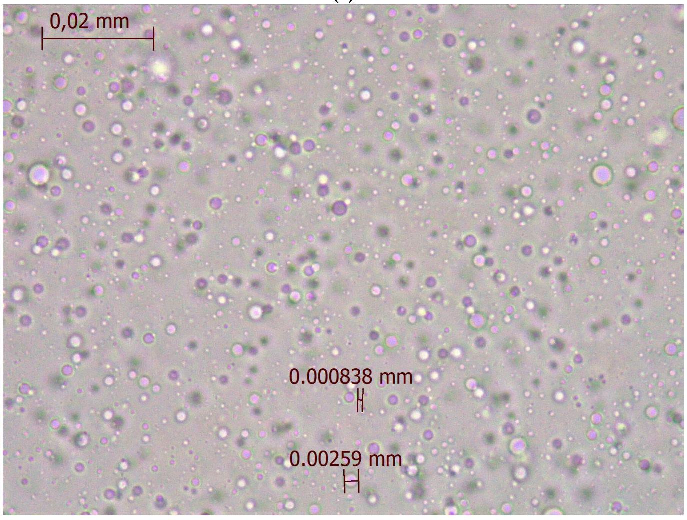
0,02 mm
0.000838 mm
0.00259 mm

(K)

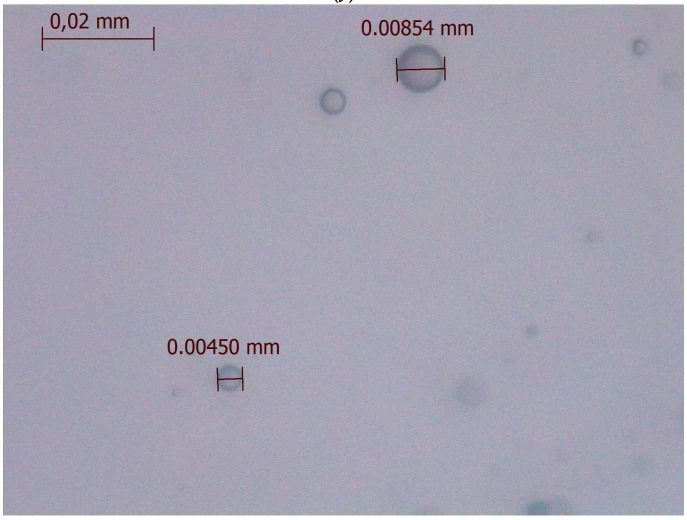
0,02 mm
0.00854 mm
0.00450 mm

(L)

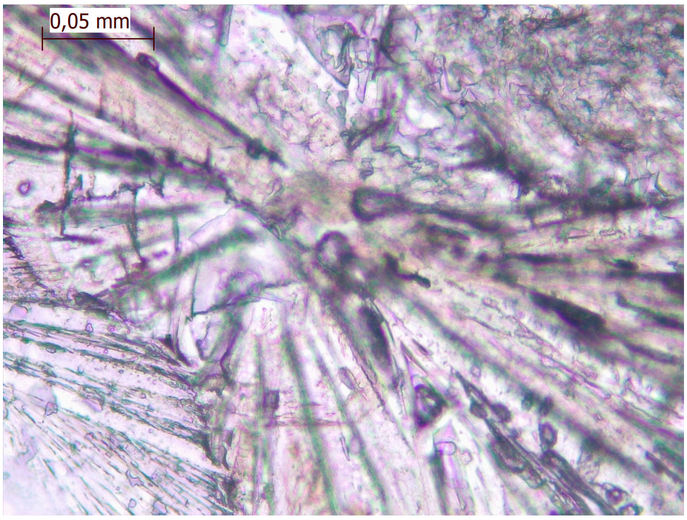

(M)

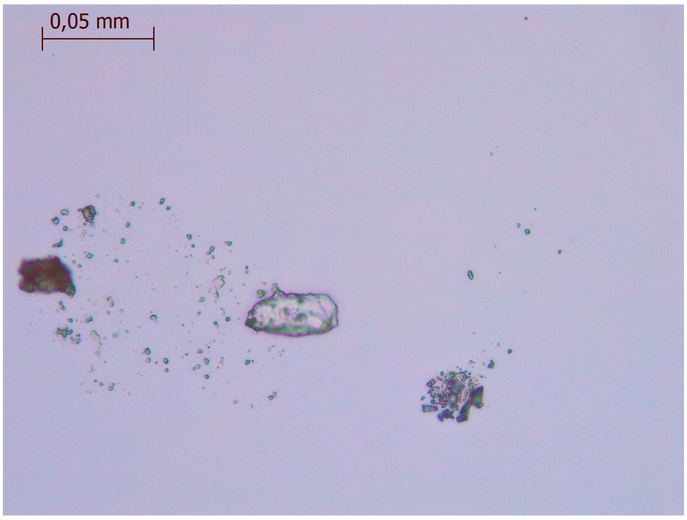

(N)

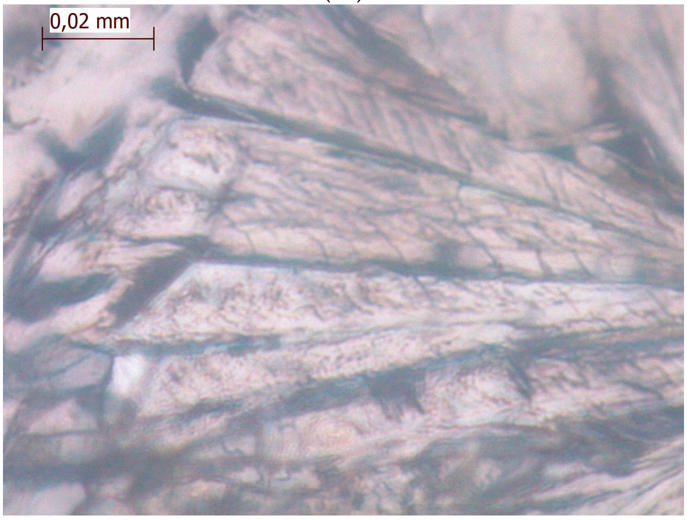

(O)

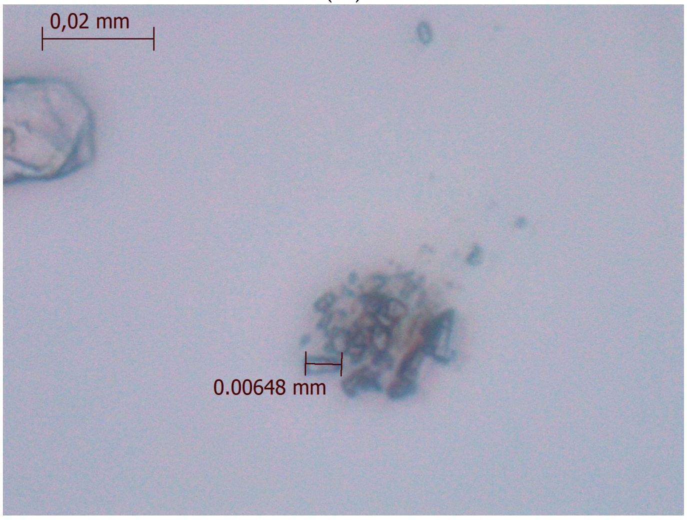

(P)

Figure S5.1. Vesicular aggregates of **2** (A, B, C, D), **3** (E, F, G, H) and **4** (I, J, K,L), as well as crystals of **1** (M, N, O, P) in MeOH (A, C, E, G, I, K, M, O) and in water solution (B, D, F, H, J, L, N,P) observed with a 40× objective (A, B, E, F, I, J, M, N,). Smaller spherical vesicles of same compound were better evidenced using the 100× objective (C, D, G, H, K, L, O, P).

Disclaimer/Publisher's Note: The statements, opinions and data contained in all publications are solely those of the individual author(s) and contributor(s) and not of MDPI and/or the editor(s). MDPI and/or the editor(s) disclaim responsibility for any injury to people or property resulting from any ideas, methods, instructions or products referred to in the content.
